# Sequence-Level Mechanisms of Human Epigenome Evolution

**DOI:** 10.1093/gbe/evu142

**Published:** 2014-06-24

**Authors:** James G.D. Prendergast, Emily V. Chambers, Colin A.M. Semple

**Affiliations:** ^1^The Roslin Institute, The University of Edinburgh, Midlothian, United Kingdom; ^2^MRC Human Genetics Unit, MRC Institute of Genetics and Molecular Medicine, University of Edinburgh, Western General Hospital, United Kingdom

**Keywords:** epigenome, methylation, chromatin, duplication, paralogous, evolution

## Abstract

DNA methylation and chromatin states play key roles in development and disease. However, the extent of recent evolutionary divergence in the human epigenome and the influential factors that have shaped it are poorly understood. To determine the links between genome sequence and human epigenome evolution, we examined the divergence of DNA methylation and chromatin states following segmental duplication events in the human lineage. Chromatin and DNA methylation states were found to have been generally well conserved following a duplication event, with the evolution of the epigenome largely uncoupled from the total number of genetic changes in the surrounding DNA sequence. However, the epigenome at tissue-specific, distal regulatory regions was observed to be unusually prone to diverge following duplication, with particular sequence differences, altering known sequence motifs, found to be associated with divergence in patterns of DNA methylation and chromatin. Alu elements were found to have played a particularly prominent role in shaping human epigenome evolution, and we show that human-specific AluY insertion events are strongly linked to the evolution of the DNA methylation landscape and gene expression levels, including at key neurological genes in the human brain. Studying paralogous regions within the same sample enables the study of the links between genome and epigenome evolution while controlling for biological and technical variation. We show DNA methylation and chromatin divergence between duplicated regions are linked to the divergence of particular genetic motifs, with Alu elements having played a disproportionate role in the evolution of the epigenome in the human lineage.

## Background

Epigenomic features, such as DNA methylation and histone modifications, are involved in a number of key cellular processes ranging from the regulation of gene expression (Li and Reinberg 2011), to splicing (Shukla et al. 2011) and the repression of transposable elements (Miura et al. 2001). Inactivation of the genes controlling DNA methylation in mice has been shown to be lethal during early development (Okano et al. 1999) and in humans, aberrant DNA methylation and chromatin patterns have been linked to a number of human diseases including cancer and various neurodevelopment disorders (Urdinguio et al. 2009; Campbell and Turner 2013).

Despite the clear importance of DNA methylation and other chromatin features to development and disease, the extent of recent human epigenome evolution and the phenomena driving such changes remain poorly understood (Bird 2011; Su et al. 2011). Genome-wide interspecies comparisons of DNA methylation and chromatin states, now possible with the advent of high-throughput sequencing technologies, have provided glimpses of the extent and nature of epigenetic divergence between species (Shibata et al. 2012; Zeng et al. 2012). However, it is still largely unclear what drives this divergence. Heritable spontaneous gains or losses of DNA methylation have been identified in plants that occur independently of genetic mutations (Schmitz et al. 2011), suggesting that DNA methylation divergence can occur independently of the underlying genomic sequence. However, particular genetic variants have also been observed to be linked to changes in DNA methylation levels within both populations (Gibbs et al. 2010) and individuals (Prendergast et al. 2012). Similarly, sites of DNA independent DNA methylation variation have been shown to be affected by rearrangements in neighboring regions (Foerster et al. 2011). Beyond the DNA sequence itself, it has been proposed that the broad epigenomic context and chromosomal location of a region may also play a role in determining DNA methylation states (Lienert et al. 2011).

Studies investigating the links between underlying genetic sequence and the divergence of DNA methylation and chromatin have predominantly examined changes between individuals or species. However, as the activity of determinants of methylation and chromatin states (such as methyltransferases) will differ between samples, these studies are confounded by natural biological variation. Likewise, the comparison of different samples will also often lead to the introduction of technical variation that can be difficult to control for. To circumvent these problems, we have investigated how patterns of DNA methylation and chromatin variation, and their link to underlying DNA sequence divergence, can be studied within a single sample. Human segmental duplications, often defined as pairs of DNA sequences greater than 1 kb in length which align with more than 90% identity (She et al. 2004a), comprise approximately 5% (∼150 Mb) of the human genome (Marques-Bonet et al. 2009). A previous study has suggested that certain histone modifications may vary between duplicated regions (Zheng 2008), but we lack a comprehensive view of epigenomic divergence between duplicons. In particular, the extent of divergence in DNA methylation state between duplicons and its relationship to changes in the underlying sequence remains unknown.

The study of divergence in the epigenome between paralogous regions has the potential to not only uncover the links between the evolution of DNA sequence, DNA methylation, and chromatin state but also allow us to investigate how duplications have potentially contributed to species evolution. Segmental duplication events have been an important mechanism by which new genes are created and current gene families expanded, providing a key mechanism for species evolution (De Grassi et al. 2008). Whether functional regulatory modules are also maintained and evolve following a duplication event has yet to be examined genome wide. If DNA methylation and chromatin states are broadly maintained following the duplication of a region then this is likely to have implications for the expression of genes in close proximity to the new duplicon. Alternatively, divergence in chromatin states following duplication potentially provides another mechanism for the evolution of a locus and the neo or subfunctionalization of a genomic region, beyond simply the evolution of the underlying genome sequence. DNA sequence is thought to evolve in a relatively clock-like fashion across the genome, with the number of changes between duplicons expected to increase with increasing time since the duplication event. Whether the epigenome evolves in the same way, and at similar rates, remains largely unknown.

In this study, we examined DNA methylation and chromatin divergence at the tens of thousands of loci that have been duplicated across the human genome to investigate the links between the evolution of the genome and epigenome in unprecedented detail. Human embryonic stem cells were used as the primary model in this study, but we validate the results in other cell types and extend our observations to examine the evolution of the human brain epigenome since divergence from chimpanzee. This study provides the first comprehensive analysis of DNA sequence, DNA methylation, and chromatin divergence across paralogous sites in the human genome.

## Results and Discussion

### Widespread Conservation of DNA Methylation and Chromatin States Following Duplication

**Fig. 1.—:**
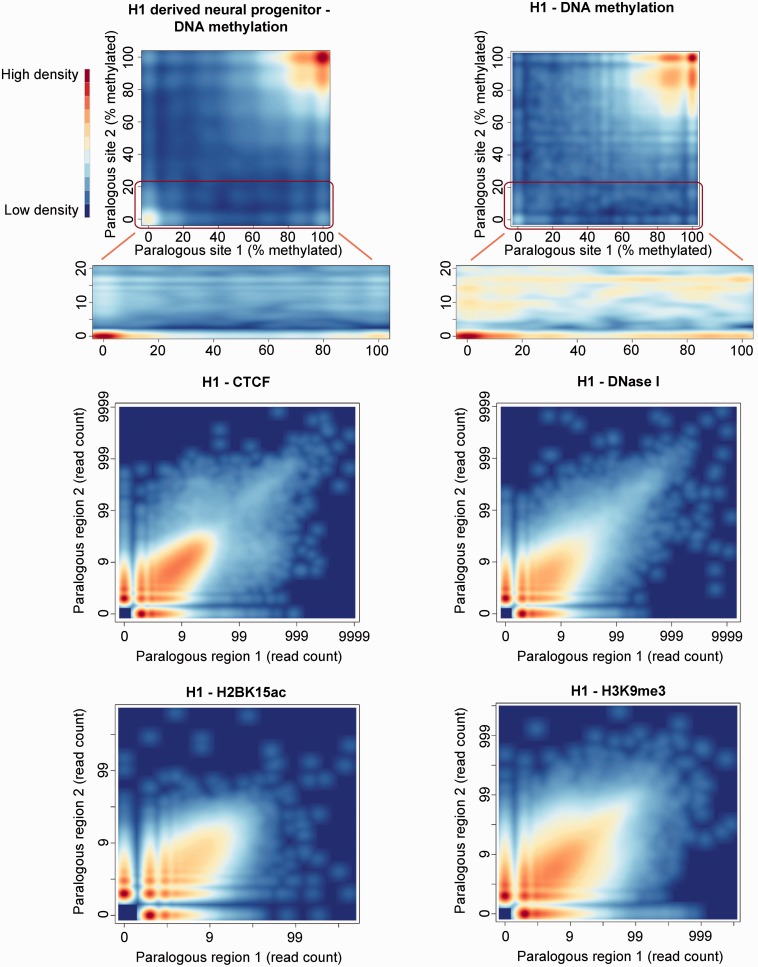
Strong conservation of DNA methylation and other chromatin features following a segmental duplication event. Comparative methylation levels at paralogous CpG sites in the H1 and H1-derived neural progenitor cell lines are shown in the top two panels (corresponding plot for IMR90 shown in supplementary fig. S1, Supplementary Material online). Intensity of color corresponds to the density of paralogous pairs of CpG sites with the corresponding methylation levels. Densities are rescaled for the subplots displaying the lower areas of the graphs in more detail. The lower four panels show the read counts from three ChIP-seq and one DNase-seq experiment found at each pair of 500-bp paralogous regions in the H1 cell line. Paralogous pairs of windows with no reads mapping to either region are excluded from these plots. The corresponding plots for all 23 histone modifications examined can be found in supplementary figure S2, Supplementary Material online.

**Fig. 2.—:**
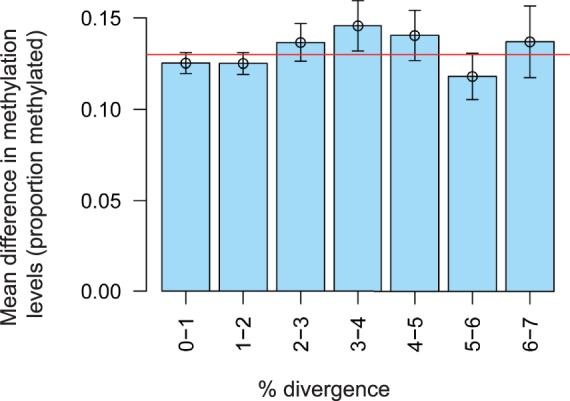
DNA methylation divergence is independent of levels of surrounding DNA sequence divergence. Mean difference in methylation levels between paralogous CpG sites associated with different levels of flanking sequence divergence. The number of single-base substitutions was measured in the DNA sequence 500 bp either side of the corresponding CpG site (or to the end of the duplicated region if closer); 95% confidence intervals are shown along with the genome-wide mean difference in methylation levels between paralogous CpG sites (red horizontal line). No significant difference was observed in the average difference in methylation levels between paralogous CpG sites of different levels of flanking sequence divergence (*P* = 0.17, Kruskal–Wallis test).

We first examined how methylation states have evolved following a segmental duplication event in the human lineage (>1 kb in length and >90% identity). Examination of paralogous CpG sites in the human genome illustrates that DNA methylation levels have been strikingly well conserved following a duplication event and the insertion of a homologous sequence into a new genomic location. As shown in figure 1 both unmethylated and methylated sites overwhelmingly maintain their approximate methylation levels at both paralogous copies of a duplicated region. Of 82,692 paralogous pairs of CpG sites examined in H1 embryonic stem (ES) cells, 78.4% displayed an absolute difference of 20% or less in methylation levels (permutation *P* value < 0.01; Spearman’s rank correlation of 0.23, *P* < 2.2 × 10^−^^16^). High levels of conservation in methylation levels were also observed in the H1-derived neural progenitor and IMR90 cell lines (permutation *P* values for both <0.01; Spearman’s rank correlations of 0.37 and 0.48, respectively, both *P* < 2.2 × 10^−^^16^; fig. 1 and supplementary fig. S1, Supplementary Material online). Both methylated and unmethylated sites were observed to generally display high levels of conservation (fig. 1). No consistent difference was observed between the methylation state of the ancestral and derived copies of paralogous CpG sites in these three cell types, with the derived copy of interchromosomal duplicated CpG sites observed to be as likely as the ancestral copy to have a low (<50%), putatively functional, methylation state (supplementary fig. S2, Supplementary Material online). Perhaps surprisingly DNA methylation divergence was observed to be largely uncoupled from the mean sequence-level divergence in the surrounding region. Although methylated CpGs predominate in the human genome, this remained the case when only examining paralogous CpG sites with at least one lowly methylated locus (<50% methylation, Kruskal–Wallis test of association between methylation and average sequence divergence as in fig. 2, *P* = 0.31). We could also find no evidence that substitutions within close proximity of the CpG site were more likely to be associated with divergence in methylation levels than those further away.

This conservation of DNA methylation levels is matched by conservation of a wide variety of chromatin features at duplicated loci. The location of various histone modifications, as well as CTCF binding (a chromatin regulator [McDaniell et al. 2010]) and DNase I hypersensitivity (a marker of functional regulatory regions [McDaniell et al. 2010]), was all well conserved between paralogous regions (fig. 1 and supplementary fig. S3, Supplementary Material online). Thus, broad chromatin states, reflected in many chromatin features, have generally been well conserved following the insertion of a DNA sequence into a new genomic location and higher order chromatin environment.

### DNA Methylation and Chromatin Divergence Are Linked to Divergence at Specific Local Sequence Motifs

**Fig. 3.—:**
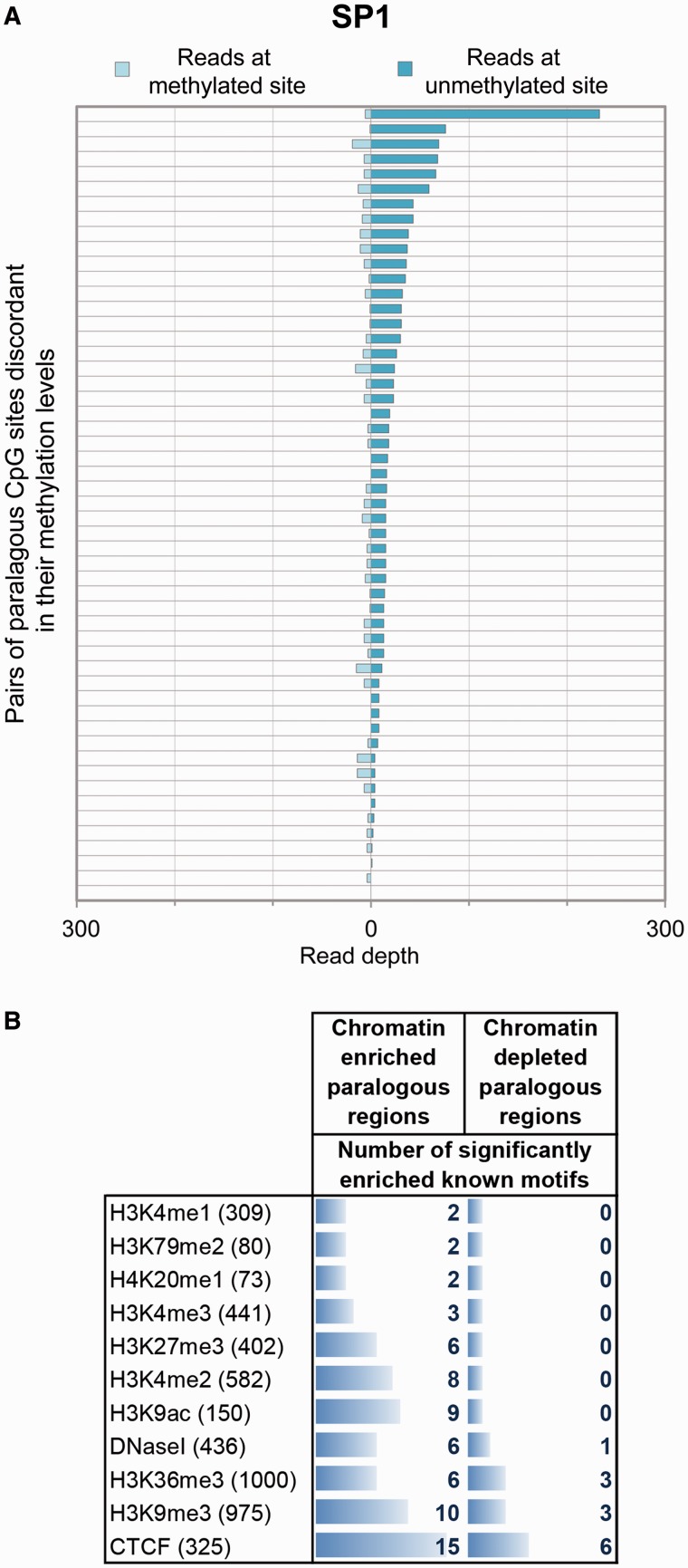
DNA methylation and chromatin divergence are associated with variation in known sequence motifs. (*A*) Loss of putative SP1-binding site at methylated copies of discordant paralogous CpG sites is associated with a corresponding lack of SP1 binding at these loci. SP1 ChIP-seq read depths 500 bp either side of the corresponding methylated and unmethylated copies of discordant paralogous CpG sites are shown. (*B*) Divergence in chromatin patterns between paralogous regions is consistently associated with the loss of transcription factor-binding sites. Only the 11 chromatin marks with at least 70 discordant pairs of regions were analyzed, with the number of discordant pairs of regions shown in brackets following the modification name. A corresponding list of each of the known sequence motifs enriched at either the chromatin enriched or depleted copies of paralogous regions can be found in supplementary table S1, Supplementary Material online.

We next investigated whether where divergence in DNA methylation and chromatin states has occurred it is related to particular changes in the underlying DNA sequence. If the evolution of the epigenome is entirely uncoupled from the evolution of the DNA sequence then no particular DNA motifs would be expected to show enrichment around either the methylated or unmethylated copies of discordantly methylated, paralogous CpG sites. However, as shown in table 1, particular motifs were observed to be linked to methylation divergence. This included a motif matching the known chromatin regulator SP1 (*q* = 0.001). Further analysis highlighted that the loss of these putative SP1-binding sites is also associated with detectable falls in the observed levels of SP1 binding at these regions (fig. 3*A*).

**Table 1:** Motifs Enriched around the Hypomethylated Copies of the Significantly Discordant (*P* < 5 × 10^−7^) Paralogous CpG Sites in Various Tissues

Motif	*P*	Top Match	Match Score	*q*	Program	Notes
H1 cell line (32 pairs of sites)					
YCCCSCCKCCTCMKCCTCCC	1.80E−25	SP1		0.001	MEME	
KKGSKGKGRRYRSGG	1.80E−04	Zfp281		0.0026	MEME	SP1 *q* = 0.0026
STYTTYTTTTYYTTTTTTTT	1.40E−29	MTF1		0.086	MEME	
SRSGSSYSAGSCMCCGYSSC	1.60E−06				MEME	
TTARDACWGT	1.00E−12				Homer	
H1-derived neural progenitor cell line (46 pairs of sites)					
TTTYTTWTTYTTTTTYTTTT	2.50E−43				MEME	
YYCWCCYKCCYCWSYCYCCC	6.10E−35	SP1		0.043	MEME	Zfp281 *q* = 0.043
SCRGGCTGGRGTSSRRKGGM	3.20E−15				MEME	
YTCYCRAAKTGYTKGKATTA	1.70E−12				MEME	
WAWWTTTKTWTKTTTADKWG	3.80E−11				MEME	
GYGAGSCASCGCSCCYGGCC	2.00E−09				MEME	SP1 *q* = 0.14
NFY (RGCCAATSRG)	NA	NFY		0.094	Homer	Known motif enrichment result
Prefrontal cortex primary tissue (22 pairs of sites)					
MWKCYYCYCCYYMMSCCYCC	8.60E−06	Zfp281		9.7 × 10^−4^	MEME	SP1 *q* = 0.053
WTYWTTKTMTTKYTTTYTW	2.10E−05				MEME	
Sites not in a CpG island (13 pairs from each cell type)					
DGGAGCGCWK	1.00E−12	MED-1	0.73		Homer	
GGCCCCCA	1.00E−12	Zfp281	0.76		Homer	

Note.—To ensure each region only appeared once in this analysis, where more than one pair of discordant CpG sites was within 1 kb only one was kept (arbitrarily chosen).

As well as the SP1 binding site, a number of other motifs were found to be enriched around the hypomethylated copies of discordant CpG sites (table 1). These include a motif matching the multiple start element downstream-1 (MED-1) sequence that was found to be associated with divergent CpG sites found outside CpG islands across cell types. This motif was found around 35.9% of the hypomethylated copies of these discordant CpG sites but at only 5.04% of the matching hypermethylated copies (*P* = 1 × 10^−^^12^). The MED-1 sequence is a downstream protein-binding element previously linked to TATA-less promoters with multiple distinct start sites (Butler and Kadonaga 2002). Although, as far as we are aware, not previously linked to DNA methylation divergence, mutations at this element within the P-glycoprotein promoter have been shown to lead to a reduction in transcription of the gene through selectively decreasing the use of alternative transcriptional start sites (TSSs) (Ince and Scotto 1995). Together these results suggest that mutations at this element lead to changes in methylation levels linked to altered transcription levels.

The divergence of many chromatin features (various histone modification levels, CTCF binding, and DNase I hypersensitivity) between paralogous regions was also found to be linked to the divergence in particular transcription factor-binding sites (supplementary table S1, Supplementary Material online). The “chromatin depleted” copies (those duplicons with relatively less of a given chromatin feature) of discordant paralogous pairs being consistently associated with a relative lack of known protein-binding motifs (fig. 3*B*). These included motifs known to be associated with particular chromatin states. For example, the known CTCF-binding site was found to be substantially depleted from the copies of paralogous regions lacking CTCF binding (supplementary table S1, Supplementary Material online), validating this approach for detecting sequence motifs linked to chromatin states; however, various other transcription factor motifs were observed to be linked to the divergence in particular chromatin marks. The full list of motifs linked to the divergence of each chromatin state can be seen in supplementary table S1, Supplementary Material online. The general link observed between transcription factor binding and chromatin divergence supports the concept of pioneer transcription factors (Zaret and Carroll 2011), whose initial binding at a region enables subsequent chromatin remodeling and the recruitment of histone modification enzymes. The approach presented here provides an indication of which transcription factors are most strongly linked to variation in particular chromatin features and might therefore act as pioneers in the H1 cell type.

### Distal Regulatory Regions Have Been Foci for DNA Methylation and Chromatin Divergence

Although DNA methylation levels have previously been shown to be correlated with local CpG content (Gaidatzis et al. 2014), strong conservation of DNA methylation levels was observed following a duplication event irrespective of local CpG density (supplementary fig. S4, Supplementary Material online). However, the subset of CpG sites in the H1 cell line most discordant in their methylation levels with respect to their paralogous copy (654 sites with a methylation difference >80%) were found to generally locate to CpG islands (8% of sites) or CpG island “shores” (51% of sites, defined here as less than 2 kb from the neighboring CpG island), regions important in gene regulation, and disease (Doi et al. 2009; Irizarry et al. 2009). Thus, methylation divergence is seen at regions where methylation levels are known to be particularly important to regulatory function and is not simply restricted to CpG poor, putatively nonfunctional regions of the genome. This is in contrast to sequence divergence, where elevated rates of divergence are often seen at nonfunctional regions of the genome lacking selective constraint.

**Fig. 4.—:**
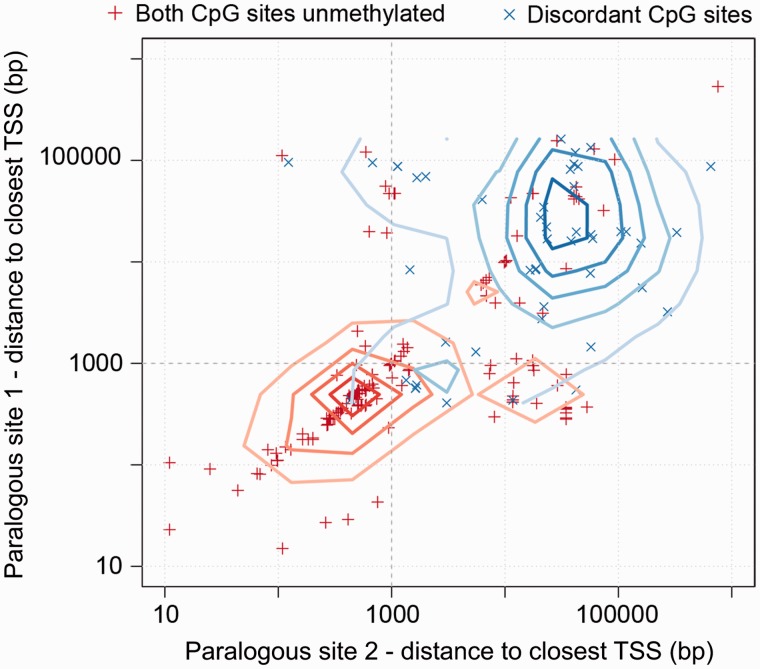
Elevated rates of divergence in methylation levels at pairs of paralogous CpG sites distal to TSSs. Distance to nearest TSSs of paralogous pairs of CpG sites completely unmethylated on both copies (red) and pairs of paralogous CpG sites significantly different in their methylation levels (blue). Contour lines correspond to a 2D kernel density estimate for each group of points highlighting the separate clustering of pairs of discordant paralogous CpG sites and paralogous sites unmethylated on both copies. Discordant pairs of CpG sites are generally greater than 1 kb from the nearest TSS.

CpG islands are often associated with gene promoters; however, examination of the proximity to promoter regions of the most significantly discordant CpG sites in the H1 cell line (53 pairs of sites with *P* < 5 × 10^−^^7^, Fisher’s exact test; supplementary table S2, Supplementary Material online) revealed that they were found almost exclusively distal to known TSSs (fig. 4). In stark contrast, methylation levels at CpG sites within 1 kb of promoter regions are strongly conserved following a duplication event (fig. 4). The specific conservation of methylation levels at proximal promoter regions is consistent with a more focused study of methylation levels at ten mouse promoter regions (Lienert et al. 2011). Thus, although DNA methylation divergence is linked to CpG islands, those CpG sites close to promoters are generally well conserved, it is the sites at more distal CpG dense regions showing the highest levels of divergence.

**Fig. 5.—:**
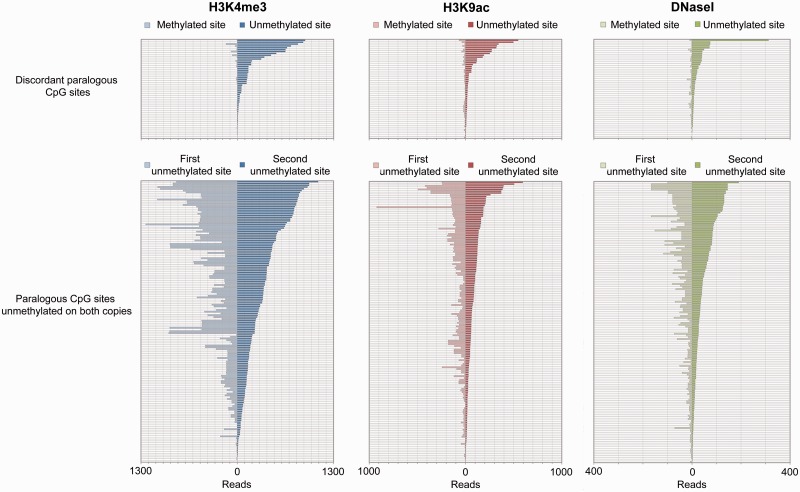
Divergence of methylation state at distal regions is associated with divergence in regulatory chromatin features. The top three panels show the total ChIP-seq/DNase-seq reads for three chromatin marks found 500 bp either side of the methylated and unmethylated copies of discordant pairs of CpG sites. Read counts are significantly higher around the unmethylated sites (corresponding plots for all 25 chromatin features examined with associated *P* values shown in supplementary fig. S5 and table S3, Supplementary Material online). Corresponding plots with the window size increased to 500 kb either of the CpG sites, illustrating this is a local effect and not a broader feature of the genomic regions, are shown in supplementary figure S11, Supplementary Material online. The bottom three panels display the read depths for the same chromatin marks at nondiscordant paralogous CpG sites completely unmethylated on both copies (CpG site labeled as “second” site being randomly chosen). Pairs of sites in each panel are sorted separately according to read counts around the unmethylated/second CpG sites.

To investigate the regulatory potential of these distal regions showing methylation divergence, we analyzed the occurrence of 23 histone modifications around these 53 most discordant CpG sites. Those associated with active regulatory regions, including H3K4me2, H3K4me3, and H3K9ac, were indeed significantly enriched around the unmethylated copies of discordant paralogous CpG sites, and depleted around the corresponding methylated copies (fig. 5 and supplementary fig. S5 and table S3, Supplementary Material online). These patterns are observed in spite of these chromatin features being generally well conserved following a duplication event (fig. 1 and supplementary fig. S2, Supplementary Material online). Other histone modifications, including H3K9me3 and H3K36me3, that are not preferentially found at regulatory regions, displayed no relative enrichment around methylated or unmethylated copies of discordant CpG pairs (supplementary fig. S5 and table S3, Supplementary Material online). In addition, the unmethylated copies of discordant CpG sites were substantially enriched for CTCF binding and DNase I hypersensitivity, general markers of functional regulatory regions (fig. 5 and supplementary fig. S5 and table S3, Supplementary Material online). We conclude that the divergence of DNA methylation levels between duplicons is associated with the evolution of other chromatin features, consistent with the emergence or destruction of distal regulatory regions in the human genome. It is consequently the small fraction of the epigenome at functional distal regulatory regions that appear to have evolved most rapidly in the human lineage.

### Sites Differentially Methylated during Differentiation Are Particularly Prone to Methylation Divergence during Evolution

**Fig. 6.—:**
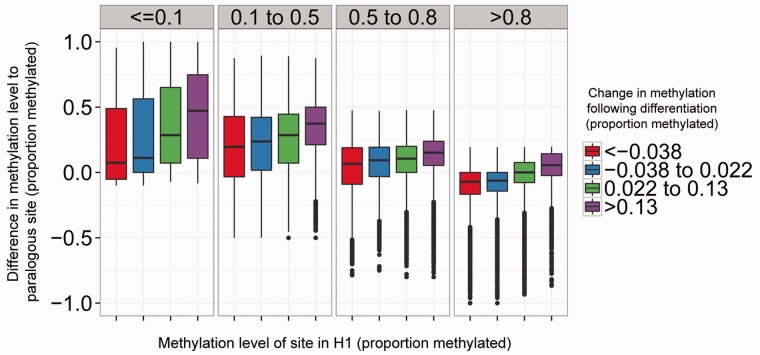
Sites of cell type-specific methylation are particularly prone to divergence following duplication. The observed difference in methylation level to their paralog of CpG sites within the H1 cell line (*y* axis). Sites are grouped by their observed methylation level in H1 (*x* axis) and their observed change in methylation following differentiation (colored bins). The cutoffs for the bins were selected, so that each category contained approximately the same number of CpG sites.

Many functional sites in the genome undergo transitions in DNA methylation during cellular differentiation and are thought to modulate regulatory interactions and transcription (Mohn and Schübeler 2009; Ong and Corces 2011). How are these sites, implicated in development and cancer (Jones 2012), related to sites showing evolutionary divergence in DNA methylation? To test this, we examined whether the difference in methylation levels between the 82,692 pairs of paralogous CpG sites in H1 ES cells was correlated to the observed change in methylation levels of the same sites between H1 and the H1-derived neural progenitor cell types. As can be seen in figure 6, in general, the larger the observed change in methylation of a CpG site following differentiation (i.e., between cell types), the larger the difference in methylation levels between the same CpG site and its paralogous site (i.e., within the same cell type). This suggests that sites showing regulated alterations in methylation during differentiation are also particularly prone to diverge following duplication in embryonic stem cells. The direction of change in methylation levels is generally the same between duplicated copies as between cell types (fig. 6) highlighting that these sites do not simply show higher variability in their methylation levels.

The extent of divergence in methylation levels following differentiation and between paralogous sites was found to be largest at sites unmethylated and lowly methylated in the H1 stem cell line (<50% methylated, fig. 6). A simple linear model incorporating the methylation level of each individual CpG site and its observed change in methylation levels following differentiation was found to be sufficient to explain a substantial proportion of the variation in the observed differences in methylation levels between paralogous CpG sites (*R*^2^: 0.33, *P* < 2.2 × 10^−^^16^). Consequently, the methylation levels of sites of cell-type-specific methylation are particularly prone to diverge following a segmental duplication event and subsequent DNA sequence divergence.

### Alu Elements Are Associated with DNA Methylation Divergence at Flanking Sites

Despite the observed links with DNA sequence motifs, one of the strongest correlates to DNA methylation divergence between paralogous CpG sites was found to be discordance in the distance to the nearest Alu element. The hypermethylated copy of the 53 discordant pairs of paralogous CpG sites in the H1 cell line was found to in general be significantly closer to an Alu element than their corresponding hypomethylated copy (supplementary fig. S6, Supplementary Material online). The other major repeat classes displayed no similar enrichment around either the methylated or unmethylated copies of discordant paralogous CpG pairs (LINE *P* = 0.85, LTR *P* = 0.74, simple repeat *P* = 0.69—paired Mann–Whitney *U* tests) suggesting that methylated sites in discordant pairs are not simply associated with regions densely populated by repeat elements, which might be expected at regions simply under less evolutionary constraint, but are specifically associated with Alu element insertion events. Alu elements have been implicated in the creation of segmental duplication events and are often found at the junctions of duplicated regions (Bailey et al. 2003). However, we could find no evidence that discordant CpG pairs were simply closer to junctions than nondiscordant CpG sites (discordant CpG sites median distance to junction: 364 bp and nondiscordant CpG sites median distance to junction: 341 bp; Mann–Whitney *U* test *P* = 0.13). These data are consistent with previous proposals that certain transposable elements may play functional roles in regulation as a result of their general high levels of methylation affecting the methylation state of nearby CpG sites (Wang et al. 2011). The results presented here suggest Alu elements may have played a substantial role in the evolution of the epigenome in the human lineage. Of the 32 pairs of paralogous regions containing CpG sites discordant in their methylation levels (i.e., the 32 pairs of regions containing the 53 significantly discordant pairs of CpG sites), the methylated copies in each pair were closer to an Alu element in 22 cases (with four pairs showing no difference in the distance to an Alu element). Of the six remaining pairs of regions, the unmethylated copy was substantially closer (>15 bp) to an Alu element in only two cases. Consequently, the methylated copies of CpG sites that have diverged following duplication are highly enriched for proximity to Alu elements.

The loss of SP1 binding and the close proximity of an Alu element were observed to often co-occur at regions divergent in their methylation levels. Of the 22 discordant pairs of CpG sites where the methylated copy was closer to an Alu element, the corresponding SP1 ChIP-seq read count was also lower at the methylated copy in 19. Although methylation levels at TSSs were observed to be generally relatively stable, one of the few sites of divergence in methylation levels at a TSS (a duplicated CpG island at the 5′-end of the TPTE and LOC400927 genes) is linked to divergence in both the proximity to the closest Alu element as well as SP1 binding (supplementary fig. S7, Supplementary Material online). A corresponding large change in expression is observed between these genes, with LOC400927 being expressed at approximately 30 times the level of TPTE in the H1 cell line (LOC400927 reads per kilobase per million reads [RPKM]: 1.79 and TPTE RPKM: 0.061). It may be that methylation levels are generally well conserved following a duplication event because such “multiple hits” are required to substantially remodel the methylation levels at a region.

### Alu Element Insertions Are Linked to the Remodeling of Methylation Patterns in the Human Brain

**Fig. 7.—:**
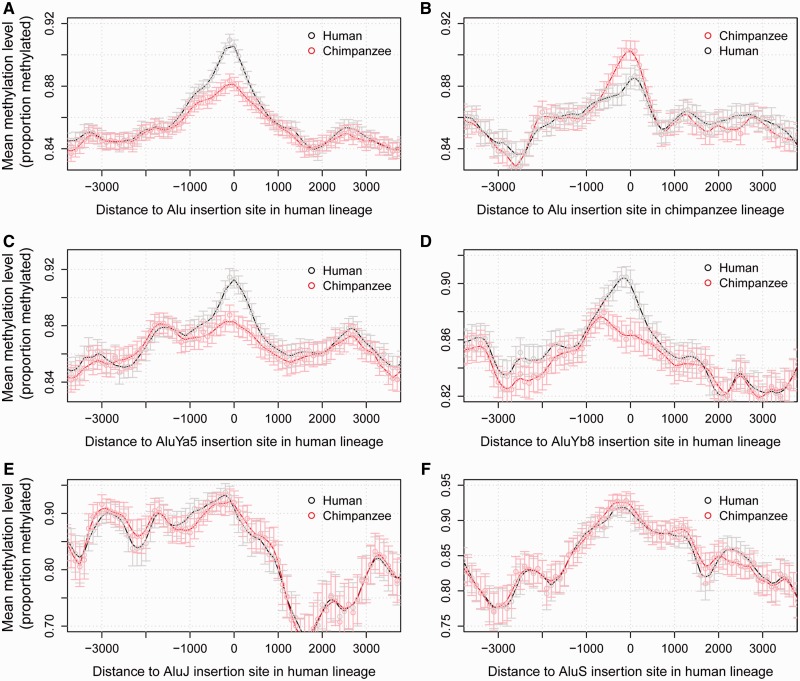
Alu elements are linked to DNA methylation divergence at flanking sites. Elevation in prefrontal cortex methylation levels at CpG sites in close proximity to Alu insertion events. Only orthologous CpG sites present in both species were retained. Sites were grouped into 500 bp windows with a 100-bp offset. Mean methylation levels for each window and corresponding 95% confidence intervals are shown. Panels correspond to methylation levels around sites of (*A*) Alu insertions in the human lineage (paired *t*-test comparing corresponding methylation levels within 1 kb of insertions site in human and chimpanzee: *P* < 2.2 × 10^−16^). (*B*) Alu insertions in the chimpanzee lineage (*P* = 7.5 × 10^−5^). (*C*) AluYa5 insertions in the human lineage (*P* = 1.5 × 10^−15^). (*D*) AluYb8 insertions in the human lineage (*P* = 8.5 × 10^−15^). (*E*) AluJ insertions in the human lineage (*P* = 0.57). (*F*) AluS insertions in the human lineage (*P* = 0.25).

To investigate further how Alu elements have potentially shaped key phenotypes in humans through affecting the evolution of the human epigenome, we looked at how these findings from paralogous regions translated to the whole genome and key methylation differences between humans and chimpanzees by characterizing the location of human-specific Alu insertions and their link to methylation divergence in primary human brain tissue. In total, we identified 4,435 Alu elements present in the human genome but absent from the corresponding orthologous regions of the chimpanzee and orangutan genomes. Average methylation levels at conserved orthologous CpG sites flanking these human Alu element insertion sites were found to be significantly higher in human prefrontal cortex samples than in matched chimpanzee samples (fig. 7*A*). This elevation in human methylation levels at sites flanking human-specific Alu insertion events was observed for sites both methylated and unmethylated in the chimpanzee genome. Examination of chimpanzee-specific insertions highlighted that these are also associated with increases in flanking methylation levels in the chimpanzee genome (fig. 7*B*). Subdivision of the human Alu insertions into families highlighted that the increase in flanking methylation levels is predominantly related to AluY insertions, the most active family in recent primate history (Chimpanzee Sequencing and Analysis Consortium 2005), with no observable changes in flanking DNA methylation levels linked to the less common AluJ and AluS insertions (fig. 7*C*–*F*). We conclude that not only at paralogous regions but also across the genome AluY element insertions in the human lineage have been linked to the remodeling of local methylation patterns, including in the human brain.

**Fig. 8.—:**
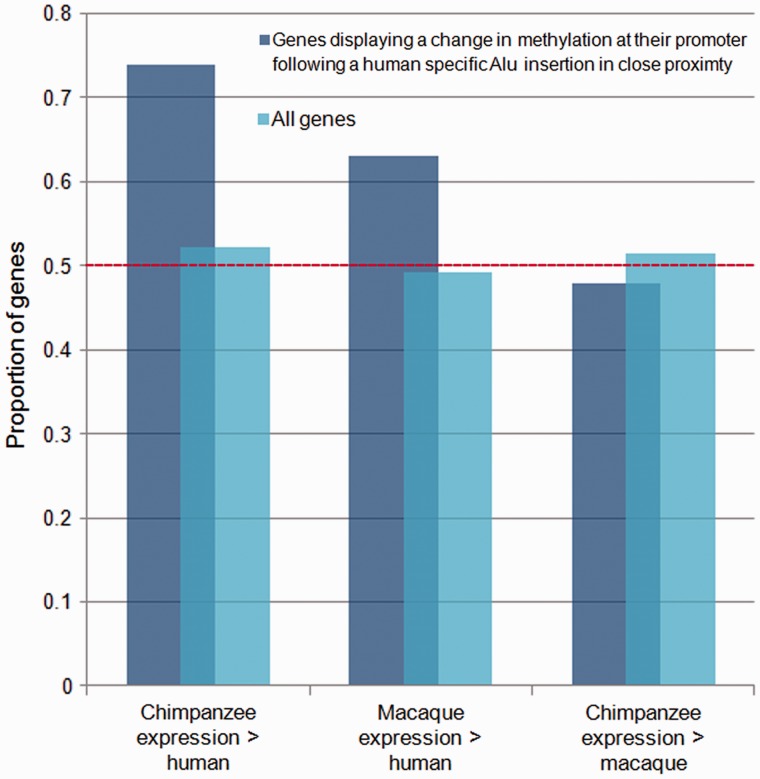
Alu insertions are linked to interspecies gene expression changes. Genes displaying a change in methylation at their promoter following a human-specific Alu insertion in close proximity also disproportionately display a corresponding lower expression level in humans.

Examination of the differences in expression levels among human, macaques, and chimpanzees where a human-specific Alu element insertion in close proximity to a gene promoter is linked to an increase in methylation illustrates that this is generally linked to a lower expression of the corresponding genes in humans relative to chimpanzees (fig. 8). Consequently, these changes in methylation levels linked to an Alu element insertion are linked to downstream changes in gene expression. Examination of the location of these CpG sites that have diverged in their methylation levels following the insertion of an Alu element in close proximity (relative to those sites where methylation levels have not changed following an Alu insertion nearby) shows that they are significantly enriched in regions harboring genes with neural functions, including those involved in neurotransmitter transport, synapse function, and insulin secretion (supplementary fig. S8, Supplementary Material online). Thus, Alu element insertion events in the human lineage appear to be directly linked to the remodeling of methylation levels around regulatory regions involved in key brain pathways are linked to interspecies changes in gene expression and may have consequently contributed to some of the key phenotypic differences between humans and our closest relatives.

## Conclusions

The evolution of the human genome sequence has been intensively studied over the past decade, providing numerous important insights into the evolution of the human lineage. However, despite their substantial importance to various traits and diseases, comparatively little is known about how DNA methylation and chromatin states evolve. Through the study of many tens of thousands of paralogous CpG sites and 25 chromatin marks, we have shown that DNA methylation and chromatin levels are surprisingly well conserved following segmental duplication events. Following the duplication of CpG islands and regulatory regions, and their insertion into a new genomic location, there is in general little divergence in DNA methylation levels or patterns of chromatin. This implies that intact regulatory modules have been copied to a new location in the human genome, and a new genomic neighborhood, while maintaining their original spectrum of DNA methylation and chromatin states. It has already been shown in vitro that the insertion of genes adjacent to previously distant regulatory regions can affect their expression patterns (Weiler and Wakimoto 1995). Just as gene duplication is now regarded as a key substrate for genome evolution, this duplication of functional, regulatory modules is likely to have provided a rich source of phenotypic variation.

Where divergence in methylation patterns did occur, it was observed to be largely uncoupled from the average rate of divergence of the surrounding DNA sequence. The gross levels of genomic and epigenomic divergence at a locus appear to be largely independent. Under the neutral theory of molecular evolution (Kimura 1989), the amount of DNA divergence between two paralogous regions should be approximately related to the time since duplication of the ancestral region. In contrast, it appears that methylation divergence between paralogous CpG sites is largely unlinked to the time since the corresponding duplication event. This argues against DNA methylation levels evolving in a neutral, clock-like fashion. Consistent with this, methylation divergence is enriched at CpG island and shore distal regulatory regions where DNA methylation levels are known to be functionally important (Doi et al. 2009; Irizarry et al. 2009). Protein-coding DNA sequences have been shown to sometimes experience unusually high levels of positive selection following duplication (Zhang 2003). Analogously, the elevated rate of DNA methylation-level divergence at functional regions relative to other CpG sites may be indicative of positive selection acting on the methylation state of duplicated regulatory regions.

The mechanisms underlying evolutionary divergence of the epigenome have until now been poorly characterized. Although DNA methylation and chromatin divergence were observed to be largely uncoupled from the average sequence divergence between paralogous regions, in this study we have shown that divergence in many features of chromatin structure between two paralogous regions is linked to divergence at particular DNA sequence motifs. For example, unmethylated copies of discordant CpG sites were preferentially associated with a GC basepair-rich motif matching the known binding site of a key chromatin regulator, SP1. Data directly assaying SP1 binding at these loci confirmed that binding was preferentially associated with the unmethylated copies of discordant CpG sites. Artificial mutations in the SP1 motif of the mouse *Gtf2a1l* promoter have previously been shown to be associated with loss of neighboring CpG methylation (Lienert et al. 2011). We have shown here that the evolution of this and other key binding motifs has been linked to the divergence of methylation levels across a range of locations in the human genome.

Particular DNA-binding motifs were also observed to have diverged between regions discordant for particular chromatin marks. In particular, discordance in the presence of particular histone modifications between paralogous regions was observed to be linked to divergence in the motifs for particular transcription factors. This suggests that the loss of key transcription factor-binding motifs leads to the loss of binding of the corresponding transcription factor at the region and the loss of the subsequent recruitment of the corresponding chromatin mark, supporting the concept of pioneer transcription factors (Zaret and Carroll 2011). Single-nucleotide polymorphisms at transcription factor motifs have recently been linked to chromatin divergence (Kasowski et al. 2013), in agreement with these findings. However, such population-based studies not only suffer from biological and technical variation but also require the assaying of chromatin states across multiple individuals. Given the links between chromatin and transcription factor motifs is likely to differ between cell types, using such population-based approaches to identify pioneer factors for each cell type can be expensive. Here, we show putative pioneer factors can potentially be identified within a single sample through the study of paralogous regions.

The majority of DNA methylation and chromatin divergence was observed to occur at distal regulatory regions. Although DNA methylation states at promoter regions have been highly conserved, these distal regions appear to have been the main reservoirs of epigenome divergence in the human lineage. Such distal regulatory regions have previously been shown to display more cell type-specific patterns of DNA methylation (Ziller et al. 2013), and we observed that sites of cell type-specific methylation were also more likely to diverge following a duplication event.

Alu elements were observed to be preferentially enriched around methylated copies of discordant paralogous CpG sites. A third of all CpG sites in the human genome is located within an Alu element, and it has been shown that the methylation of these CpG sites can be transcriptionally repressive and increase their mutation rate (due to the high deamination rate of methylated cytosines) ultimately leading to the loss of their activity (Cordaux and Batzer 2009). A “seed and spread model” has been proposed to explain the observed patterns of DNA methylation in the genome where methylation at one region can spread to neighboring sites (Turker 2002; Zhang et al. 2012). It has also been shown that repetitive elements can potentially act as seeds, with the insertion of repetitive elements adjacent to the *INSL6* promoter leading to the de novo methylation of specific CpG sites at the region (Zhang et al. 2012). The results presented here support the hypothesis that close proximity to repetitive elements can alter the methylation state of nearby sites and that Alu elements have substantially shaped the evolution of the human epigenome. Transcription factor binding appears broadly to abrogate DNA methylation at a local region, whereas the presence of Alu elements is associated with increased methylation levels. We highlight that both factors can occur together to reshape the epigenome at regions of otherwise generally strong methylation conservation. These results support the model that DNA methylation can spread from seed regions, such as Alu elements, but be blocked by barriers such as transcription factor binding (Turker 2002; Zhang et al. 2012). We have shown that recent Alu element insertions in the human genome are linked to the remodeling of local methylation patterns in human brain cells, with sites of Alu-associated methylation remodeling preferentially linked to interspecies differences in gene expression and regions associated with key neurological pathways. It is important to note that methylation divergence is not at the Alu element itself, but at conserved sites often hundreds of basepairs from the insertion site that diverge in their methylation levels. These results suggest Alu elements are not always neutral or pathogenic additions to the human genome but may have driven key changes in the human epigenome, leading to important phenotypic differences between humans and other primates.

A substantial literature attests to the importance of gene duplication and the divergence of sister copies during the evolution of protein-coding genes (Zhang 2003). Here, we provide evidence of analogous processes acting at the level of DNA methylation and chromatin structure to affect regulatory evolution across the human genome. We show that regulatory modules (particularly at promoters) can be copied, inserted into new chromosomal environments, and usually maintain their original chromatin states. On the other hand, particular duplicated distal regulatory elements have diverged to adopt different chromatin states and presumably different functions. The mechanisms underlying this chromatin divergence appear to be linked to surprisingly specific sequence-level changes, underlining the interplay of genome and epigenome in recent human evolution.

## Materials and Methods

### Data Sets

The locations and alignments of human segmental duplication events greater than 1 kb in length and over 90% identical between regions were obtained from http://humanparalogy.gs.washington.edu/build36/align_both/ (last accessed July 1, 2014) (She et al. 2004b). The distribution of sizes of these duplicated regions is shown in supplementary figure S9, Supplementary Material online, and the genomic preferences of segmental duplications have previously been documented, with many showing an association to regions of known chromosomal instability and rearrangement such as those at subtelomeric and pericentromeric regions (Bailey et al. 2001; She et al. 2004b). They are also enriched within relatively gene rich chromosomes (Bailey et al. 2002). In total, 159 Mb of the genome is involved in at least one of these duplication events. Assuming neutrality and a molecular clock, this is the fraction of the human genome that has undergone duplication within the past 35 Myr of primate evolution (Bailey and Eichler 2006). The H1, H1-derived neural progenitor, and IMR90 whole-genome bisulfite sequencing data sets were obtained from the National Institute of Health (NIH) Roadmap Epigenomics project (http://www.ncbi.nlm.nih.gov/geo/roadmap/epigenomics/?view=matrix, last accessed July 1, 2014.) (Lister et al. 2009). Histone modification, transcription factor ChIP-seq, and DNase-seq data were obtained from a combination of both the Encyclopedia of DNA Elements (ENCODE Project Consortium et al. 2012) and NIH Epigenomics Roadmap (Bernstein et al. 2010) projects. A full list of the histone modification data sets used in this study can be found in the following file (http://datashare.is.ed.ac.uk/bitstream/handle/10283/239/1756-8935-5-6-s6.xlsx, last accessed July 1, 2014).

### Read Mapping

To enable the accurate study of chromatin at segmentally duplicated regions only reads that could be unambiguously assigned to a single region in the reference genome were included in all analyses in this study. ChIP-seq reads were first trimmed to the first base whose quality was 20 or below using FastX-Toolkit (http://hannonlab.cshl.edu/fastx_toolkit/index.html, last accessed July 1, 2014) to remove low-quality read sections. Reads were then mapped to the hg18 reference genome using bowtie (Langmead et al. 2009) with the −e 1 and −m 1 parameters ensuring only reads that uniquely mapped to one region with no mismatches were retained. Whole-genome bisulfite sequencing reads were mapped to the reference genome using Bismark (Krueger and Andrews 2011). Bismark fully bisulfite converts sequence reads and maps each to bisulfite converted versions of the reference genome, with only reads producing a unique best alignment to a region being kept. Reads that contained any mismatches to the genome that could not be attributed to bisulfite conversion (C->T or G->A) on the appropriate strand were discarded. H1 RNA-seq data from the ENCODE and NIH epigenome roadmap projects were obtained and analyzed as previously described (Prendergast et al. 2012).

### DNA Methylation Analysis

The location of duplicated CpG sites on both strands of the reference sequence was first determined, and these sites were then stringently filtered according to the following criteria. Sites where a known polymorphism (dbSNP 135) overlapped the cytosine in a CpG site, or was located at either flanking base, were excluded. Likewise, any CpG sites overlapped by reads carrying alternative alleles (excluding those expected from bisulfite conversion) were excluded, and all CpG sites had to be covered by at least one read supporting the presence of a cytosine at the corresponding position. The number of bisulfite converted and unconverted reads overlapping each CpG site was counted, and sites where either CpG site was covered by less than six reads were excluded (H1 median depth at paralogous CpG sites: 5, mean depth: 8.13, standard deviation [SD]: 15.27; H1np median: 7, mean: 8.91, SD: 11.69; and IMR90 median: 6, mean: 8.75, SD: 16.31). Sites where total read coverage across the two sites exceeded 100 were also excluded (to exclude sites displaying significant but only marginal differences between paralogous regions). Having applied these filters 82,692, 127,187, and 85,966 paralogous pairs of CpG sites remained in the H1, H1-derived neural progenitor, and IMR90 data sets, respectively. To assess whether the concordance between methylation levels of paralogous CpG sites was more than would be expected by chance CpG sites were randomly shuffled between all duplicated regions 100 times. In all permutations the proportion of sites displaying *a* ≤ 20% difference in methylation levels was lower than that observed in the unpermuted data. Only paralogous pairs of CpG sites with a *P* value smaller than 5 × 10^−^^7^ (Fisher’s exact test) were deemed to be significantly differentially methylated (corresponding approximately to a Bonferroni-corrected *P* value of 0.05 in each analysis). The ancestral and derived copies of interchromosomal paralogous CpG sites were determined by lifting both sites over to the PanTro4 chimpanzee genome. If both sites lifted over to the same chromosome, the site on the syntenic human chromosome was determined to be the ancestral copy. The ancestral copy of 383 (11.9%), 987 (12.8%), and 2,065 (9.7%) pairs of interchromosomal paralogous CpG sites with at least one copy with a methylation level less than 50% in the H1, H1-derived neural progenitor, and IMR90 cell lines, respectively, were successfully determined in this way.

### Linear Modeling

The relationship between methylation changes between cell types and the divergence in methylation levels observed between paralogous sites was modeled using multiple linear regression. The change in methylation levels observed between the H1 and H1-derived neural progenitor cell lines and the methylation level of the same site in the H1 cell line were fitted as explanatory variables along with an interaction term, that is, the equation was of the form:

$$Y_{\text{i}}=\beta_{0}+\beta_{\text{1}}X_{i}{}_{\text{1}}+\beta_{\text{1}}X_{i\text{2}}+\beta_{\text{1}}X_{i\text{1}}X_{i\text{2}}+\varepsilon_{\text{1}},$$

where *X_i_*_1_ corresponds to the observed methylation level of the *i*-th CpG site in the H1 cell line, *X_i_*_2_ is the observed methylation change between the H1 and H1-derived neural progenitor cell lines of the same *i*-th site, and *Y_i_* corresponds to the *i*-th sites observed difference in methylation level to its paralogous site. Both variables and the interaction term were highly significantly linked to the corresponding sites difference in methylation to its paralogous site within the H1 cell line (*P* < 2.2 × 10^−^^16^).

### ChIP-Seq Analysis

Conservation of chromatin patterns between paralogous regions was determined from ChIP-seq data by first excluding pairs of aligned bases to which reads could not be uniquely mapped in both regions. Mapped reads less than 35 bp were discarded and regions of 35 bp that were not unique in the genome were identified using the wgEncodeDukeUniqueness35bp table from the UCSC genome browser (Kent et al. 2002). Corresponding sites that were not unique at either duplicated copy of a region were ignored, and the number of reads mapping to the remaining positions at each nonoverlapping 500 bp region counted. Regions of discordant chromatin state were identified using a binomial test. Only pairs of paralogous sites with a Bonferroni-corrected *P* value less than 0.05 and where one copy of the region had a read count of zero (to restrict to sites where binding has been completely lost on one copy) were used in the analysis of the divergence of underlying DNA motifs.

### Motif Analysis

DNA motifs enriched 500 bp either side of discordant CpG sites were identified using MEME (Bailey and Elkan 1994) and HOMER (Heinz et al. 2010). To ensure each region only appeared once in this analysis, where more than one of the discordant CpG sites was within 1 kb only one (arbitrarily chosen) paralogous pair of sites was kept. Following this filtering, 32 pairs of sites remained in the H1 analysis, 46 in the H1-derived neural progenitor data set, and 22 in the study of the human prefrontal cortex. To identify motifs linked to methylation divergence at sites outside CpG islands across tissues, 13 pairs of sites were randomly selected from each data set (13 being the number of pairs of CpG sites not linked to a CpG island in the prefrontal cortex data set, the smallest number of pairs across these data sets). Discriminative motif discovery was performed by providing the regions around the methylated and unmethylated sites separately, and reversing the background and foreground sets to discover motifs enriched around both groups of sites. Locations of repeats in the human genome were obtained from the UCSC genome browser (Kent et al. 2002).

The HOMER program (Heinz et al. 2010) was used to determine motifs that had diverged between paralogous regions discordant in their chromatin states. Only histone modifications with at least 70 discordant regions could be successfully analyzed with HOMER. Known sites with a corresponding Benjamini *q* value less than 0.01 were treated as enriched between discordant regions.

To detect an enrichment of repeat elements around methylated copies of discordant CpG pairs, the locations of repeats in the human genome were obtained from the RepeatMasker track at the UCSC genome browser (Kent et al. 2002).

### Primate Brain Methylation Divergence

Human-specific Alu insertions were characterized by identifying Alu sequences present in the human genome but absent from the orthologous region of the chimpanzee and orangutan genomes in the UCSC genome browser chained alignments (Kent et al. 2002). The same approach was used to identify chimpanzee-specific insertions. In total 4,435 human-specific and 1,882 chimpanzee-specific Alu insertion events were identified. BS-seq data corresponding to three human and three chimpanzee prefrontal cortex samples were obtained from Zeng et al. (2012). Reads were mapped to the hg19 and PanTro3 genomes using Bismark (Krueger and Andrews 2011) with duplicate reads subsequently removed. Data were combined across the three replicates for each species and the level of methylation for each CpG site conserved across both species was determined. Sites with a combined depth of less than five reads in either species were excluded. Gene level, processed RNA-seq expression data for human, chimpanzee, and macaque (Zeng et al. 2012) were obtained from http://www.ncbi.nlm.nih.gov/geo/query/acc.cgi?acc=GSE33587 (last accessed July 1, 2014).

Functional enrichment analysis was performed using GREAT (McLean et al. 2010) by comparing all CpG sites of low methylation in the chimpanzee genome (<40% methylated) within 2 kb of a human-specific Alu insertion site, to those subset of these sites where the human methylation proportion has increased by an absolute proportion of at least 0.6 relative to the chimpanzee methylation level (i.e., had gone from a low- to a high-methylation level). This allowed us to identify regions where the methylation level showed evidence of having been substantially remodeled following the Alu insertion.

## Supplementary Material

Supplementary figures S1–S11 and tables S1–S3 are available at *Genome Biology and Evolution* online (http://www.gbe.oxfordjournals.org/).

Supplementary Data

## References

[evu142-B1] Bailey JA, Liu G, Eichler EE (2003). An Alu transposition model for the origin and expansion of human segmental duplications. Am J Hum Genet..

[evu142-B2] Bailey JA, Yavor AM, Massa HF, Trask BJ, Eichler EE (2001). Segmental duplications: organization and impact within the current human genome project assembly. Genome Res..

[evu142-B3] Bailey JA (2002). Recent segmental duplications in the human genome. Science.

[evu142-B4] Bailey TL, Elkan C (1994). Fitting a mixture model by expectation maximization to discover motifs in biopolymers. Proc Int Conf Intell Syst Mol Biol..

[evu142-B53] Bailey JA, Eichler EE (2006). Primate segmental duplications: crucibles of evolution, diversity and disease. Nat Rev Genet..

[evu142-B5] Bernstein BE (2010). The NIH roadmap epigenomics mapping consortium. Nat Biotechnol..

[evu142-B6] Bird A (2011). Putting the DNA back into DNA methylation. Nat Genet..

[evu142-B7] Butler JEF, Kadonaga JT (2002). The RNA polymerase II core promoter: a key component in the regulation of gene expression. Genes Dev..

[evu142-B8] Campbell MJ, Turner BM (2013). Altered histone modifications in cancer. Adv Exp Med Biol..

[evu142-B9] Chimpanzee Sequencing and Analysis Consortium (2005). Initial sequence of the chimpanzee genome and comparison with the human genome. Nature.

[evu142-B10] Cordaux R, Batzer MA (2009). The impact of retrotransposons on human genome evolution. Nat Rev Genet..

[evu142-B11] De Grassi A, Lanave C, Saccone C (2008). Genome duplication and gene-family evolution: the case of three OXPHOS gene families. Gene.

[evu142-B12] Doi A (2009). Differential methylation of tissue- and cancer-specific CpG island shores distinguishes human induced pluripotent stem cells, embryonic stem cells and fibroblasts. Nat Genet..

[evu142-B13] ENCODE Project Consortium (2012). An integrated encyclopedia of DNA elements in the human genome. Nature.

[evu142-B14] Foerster AM, Dinh HQ, Sedman L, Wohlrab B, Mittelsten Scheid O (2011). Genetic rearrangements can modify chromatin features at epialleles. PLoS Genet..

[evu142-B15] Gaidatzis D (2014). DNA sequence explains seemingly disordered methylation levels in partially methylated domains of mammalian genomes. PLoS Genet..

[evu142-B16] Gibbs JR (2010). Abundant quantitative trait loci exist for DNA methylation and gene expression in human brain. PLoS Genet..

[evu142-B17] Heinz S (2010). Simple combinations of lineage-determining transcription factors prime cis-regulatory elements required for macrophage and B cell identities. Mol Cell..

[evu142-B18] Ince TA, Scotto KW (1995). A conserved downstream element defines a new class of RNA polymerase II promoters. J Biol Chem..

[evu142-B19] Irizarry RA (2009). The human colon cancer methylome shows similar hypo- and hypermethylation at conserved tissue-specific CpG island shores. Nat Genet..

[evu142-B20] Jones PA (2012). Functions of DNA methylation: islands, start sites, gene bodies and beyond. Nat Rev Genet..

[evu142-B21] Kasowski M (2013). Extensive variation in chromatin states across humans. Science.

[evu142-B22] Kent WJ (2002). The human genome browser at UCSC. Genome Res..

[evu142-B23] Kimura M (1989). The neutral theory of molecular evolution and the world view of the neutralists. Genome.

[evu142-B24] Krueger F, Andrews SR (2011). Bismark: a flexible aligner and methylation caller for Bisulfite-Seq applications. Bioinformatics.

[evu142-B25] Langmead B, Trapnell C, Pop M, Salzberg SL (2009). Ultrafast and memory-efficient alignment of short DNA sequences to the human genome. Genome Biol..

[evu142-B26] Li G, Reinberg D (2011). Chromatin higher-order structures and gene regulation. Curr Opin Genet Dev..

[evu142-B27] Lienert F (2011). Identification of genetic elements that autonomously determine DNA methylation states. Nat Genet..

[evu142-B28] Lister R (2009). Human DNA methylomes at base resolution show widespread epigenomic differences. Nature.

[evu142-B29] Marques-Bonet T, Girirajan S, Eichler EE (2009). The origins and impact of primate segmental duplications. Trends Genet..

[evu142-B30] McDaniell R (2010). Heritable individual-specific and allele-specific chromatin signatures in humans. Science.

[evu142-B31] McLean CY (2010). GREAT improves functional interpretation of cis-regulatory regions. Nat Biotechnol..

[evu142-B32] Miura A (2001). Mobilization of transposons by a mutation abolishing full DNA methylation in *Arabidopsis*. Nature.

[evu142-B33] Mohn F, Schübeler D (2009). Genetics and epigenetics: stability and plasticity during cellular differentiation. Trends Genet..

[evu142-B34] Okano M, Bell DW, Haber DA, Li E (1999). DNA methyltransferases Dnmt3a and Dnmt3b are essential for de novo methylation and mammalian development. Cell.

[evu142-B35] Ong C-T, Corces VG (2011). Enhancer function: new insights into the regulation of tissue-specific gene expression. Nat Rev Genet..

[evu142-B36] Prendergast JGD, Tong P, Hay DC, Farrington SM, Semple CAM (2012). A genome-wide screen in human embryonic stem cells reveals novel sites of allele-specific histone modification associated with known disease loci. Epigenetics Chromatin.

[evu142-B37] Schmitz RJ (2011). Transgenerational epigenetic instability is a source of novel methylation variants. Science.

[evu142-B38] She X (2004a). Shotgun sequence assembly and recent segmental duplications within the human genome. Nature.

[evu142-B39] She X (2004b). The structure and evolution of centromeric transition regions within the human genome. Nature.

[evu142-B40] Shibata Y (2012). Extensive evolutionary changes in regulatory element activity during human origins are associated with altered gene expression and positive selection. PLoS Genet..

[evu142-B41] Shukla S (2011). CTCF-promoted RNA polymerase II pausing links DNA methylation to splicing. Nature.

[evu142-B42] Su Z, Han L, Zhao Z (2011). Conservation and divergence of DNA methylation in eukaryotes: new insights from single base-resolution DNA methylomes. Epigenetics.

[evu142-B43] Turker MS (2002). Gene silencing in mammalian cells and the spread of DNA methylation. Oncogene.

[evu142-B44] Urdinguio RG, Sanchez-Mut JV, Esteller M (2009). Epigenetic mechanisms in neurological diseases: genes, syndromes, and therapies. Lancet Neurol..

[evu142-B45] Wang X (2011). Spreading of Alu methylation to the promoter of the MLH1 gene in gastrointestinal cancer. PLoS One.

[evu142-B46] Weiler KS, Wakimoto BT (1995). Heterochromatin and gene expression in *Drosophila*. Annu Rev Genet..

[evu142-B47] Zaret KS, Carroll JS (2011). Pioneer transcription factors: establishing competence for gene expression. Genes Dev..

[evu142-B48] Zeng J (2012). Divergent whole-genome methylation maps of human and chimpanzee brains reveal epigenetic basis of human regulatory evolution. Am J Hum Genet..

[evu142-B49] Zhang J (2003). Evolution by gene duplication: an update. Trends Ecol Evol..

[evu142-B50] Zhang Y (2012). Repetitive elements and enforced transcriptional repression co-operate to enhance DNA methylation spreading into a promoter CpG-island. Nucleic Acids Res..

[evu142-B51] Zheng D (2008). Asymmetric histone modifications between the original and derived loci of human segmental duplications. Genome Biol..

[evu142-B52] Ziller MJ (2013). Charting a dynamic DNA methylation landscape of the human genome. Nature.

