# Supplementary material for: Sequence-Level Mechanisms of Human Epigenome Evolution
Source: Genome Biol Evol. 2014 Jun 24;6(7):1758–71. doi: 10.1093/gbe/evu142 (PMC4122940; doi:10.1093/gbe/evu142)
Supplement: Supplementary Data [file supp_6_7_1758__index.html]

Sequence level mechanisms of human epigenome evolution — Sequence-Level Mechanisms of Human Epigenome Evolution — Supplementary Data 

# Sequence-Level Mechanisms of Human Epigenome Evolution

## Supplementary Data

files

**Files in this Data Supplement:**

- Supplementary Data - docx file
