# Supplementary material for: Sequence-Level Mechanisms of Human Epigenome Evolution
Source: Genome Biol Evol. 2014 Jun 24;6(7):1758–71. doi: 10.1093/gbe/evu142 (PMC4122940; doi:10.1093/gbe/evu142)
Supplement: Supplementary Data [file supp_evu142_supplementalData_GBE_resub2.docx]

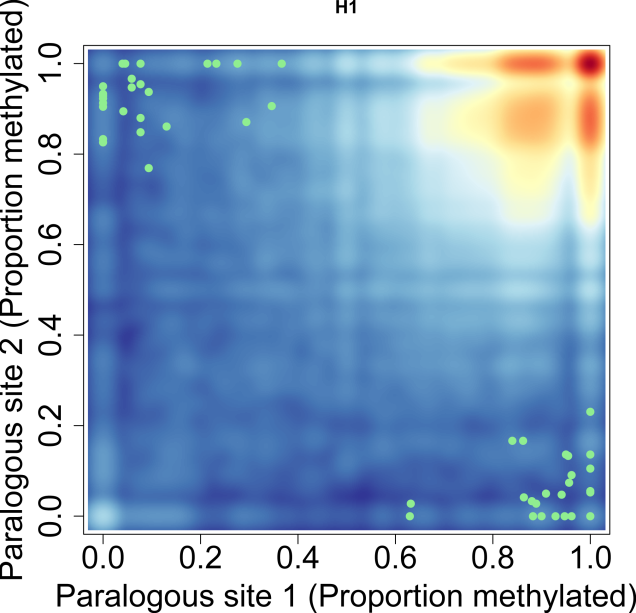

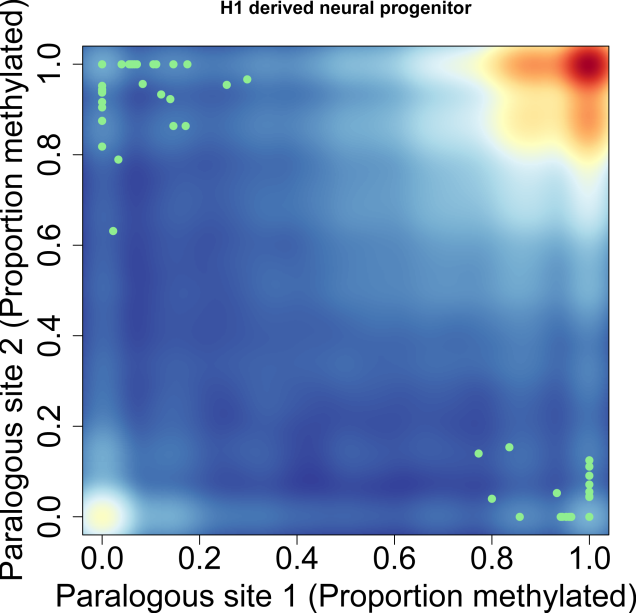


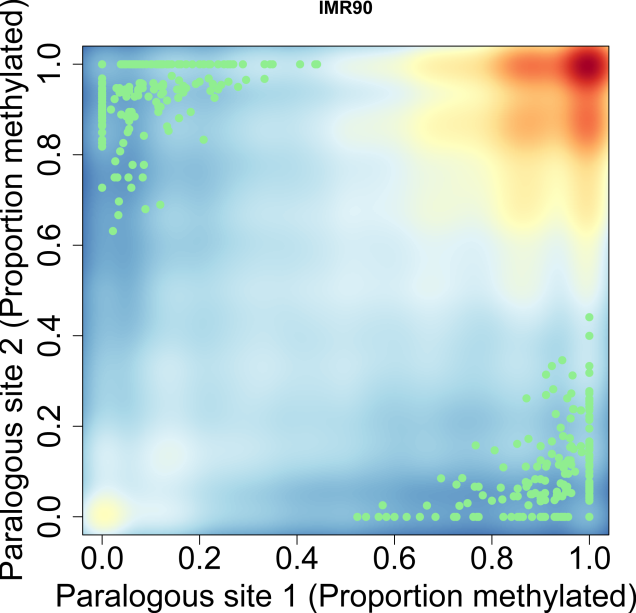


**Figure S1**: Corresponding methylation levels at matching, paralogous CpG sites in the H1, H1 derived neural progenitor and IMR90 cell lines. Intensity of colour (blue to red) corresponds to the relative density of analogous pairs of CpG sites with the corresponding methylation levels. Pairs of paralogous CpG sites significantly different in their observed methylation levels are indicated by light green points.


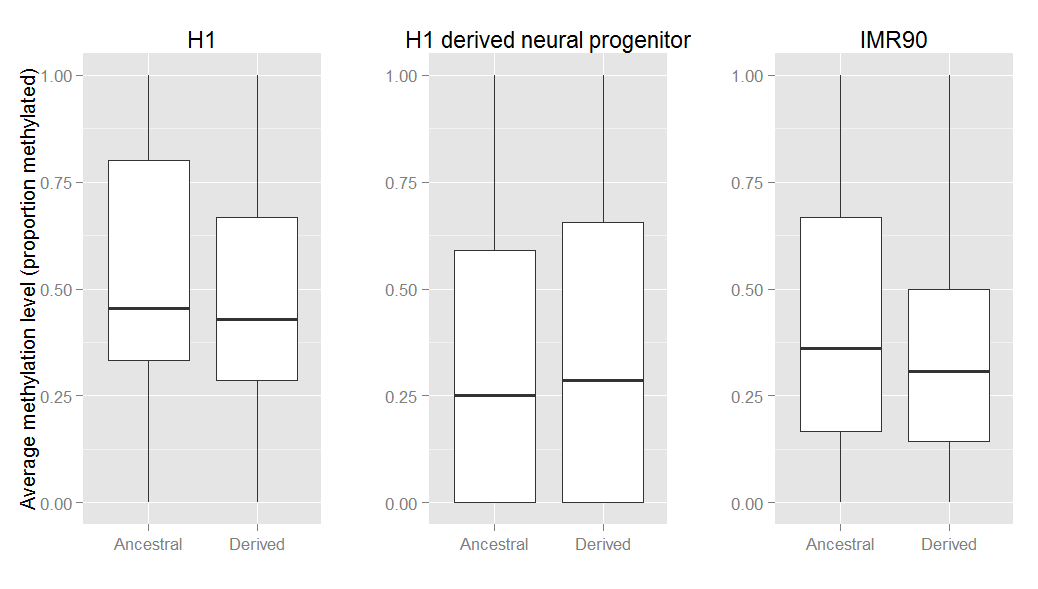


**Figure S2**: Average methylation levels at ancestral and derived copies of paralogous CpG sites where at least one of the sites had a methylation level less than 50%.


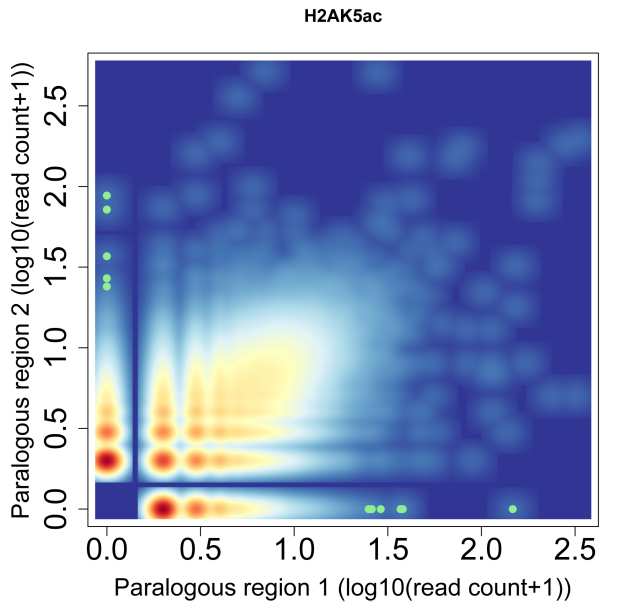

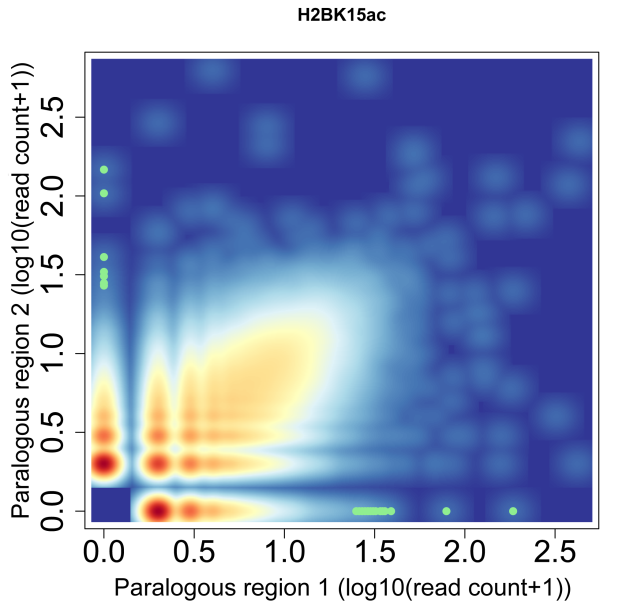


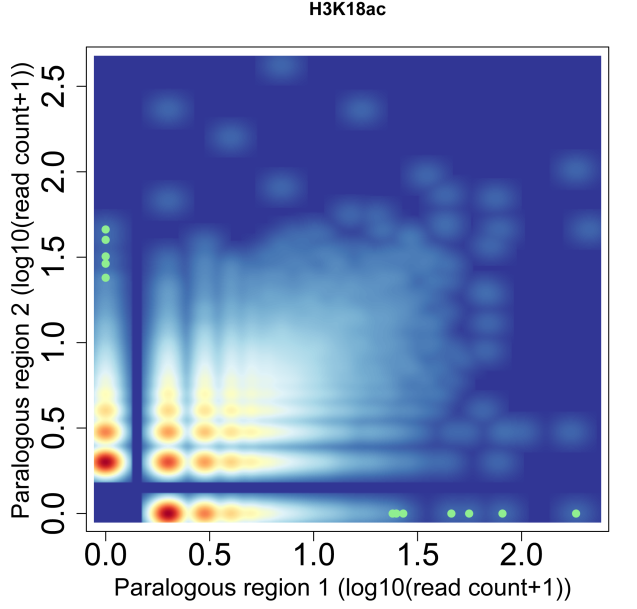

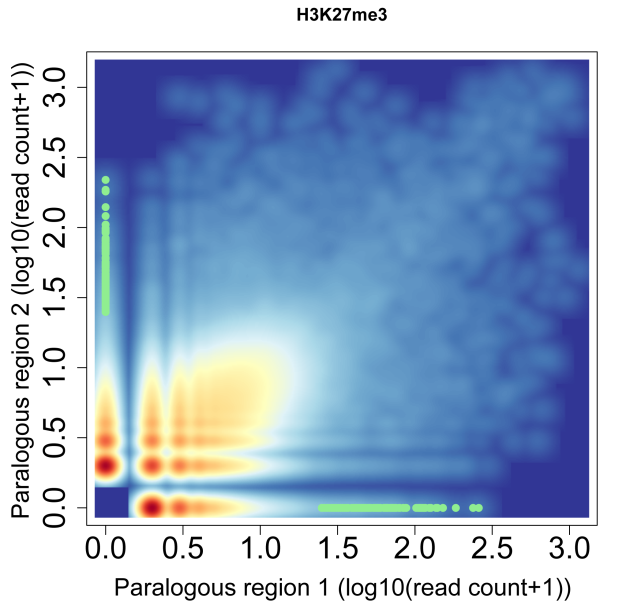


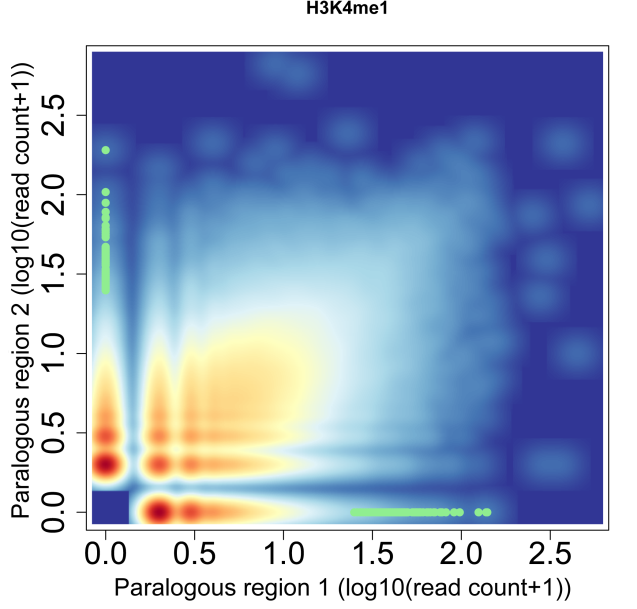

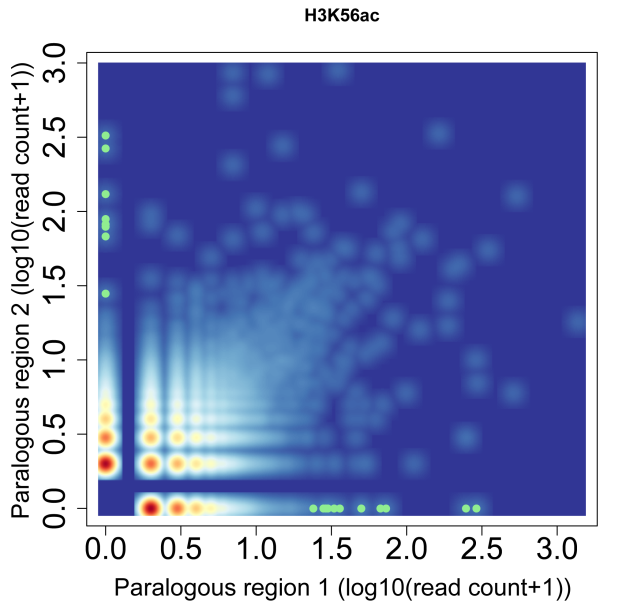


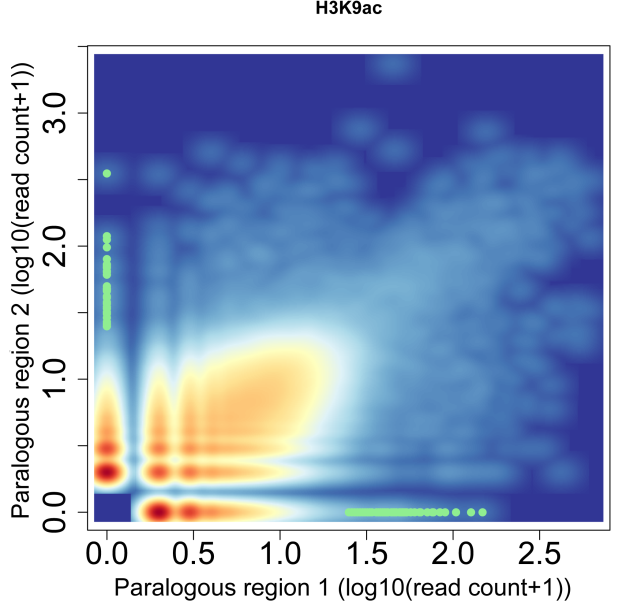

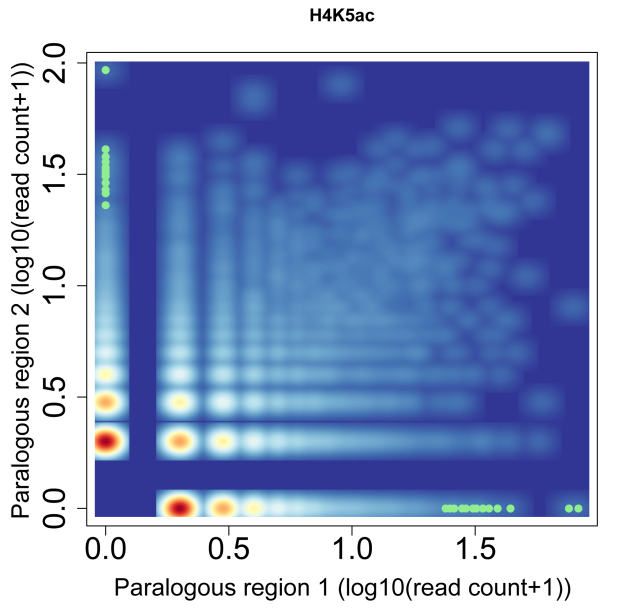


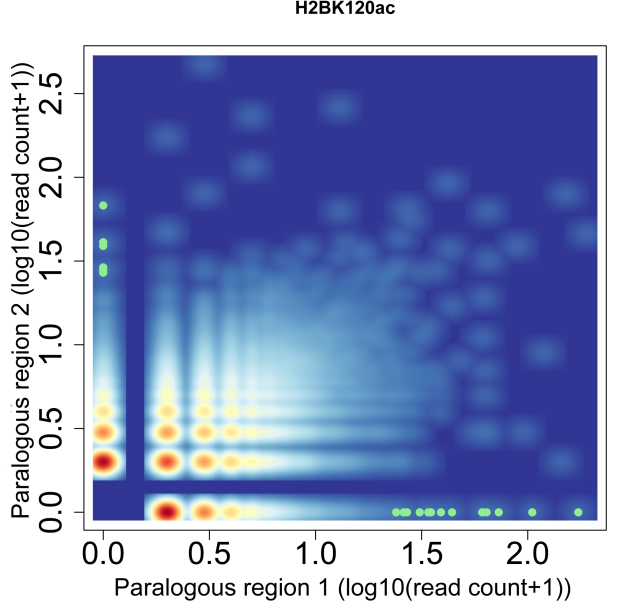

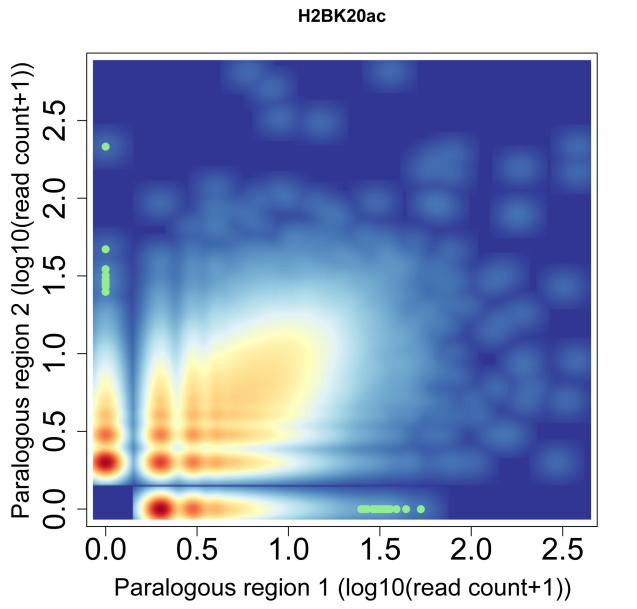


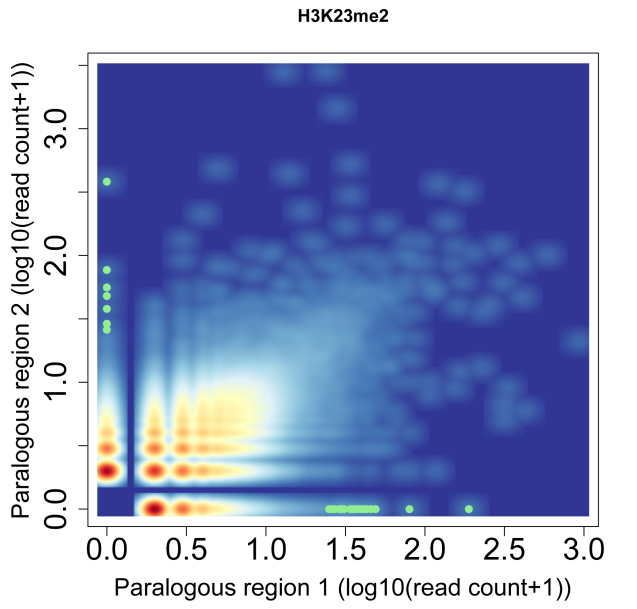

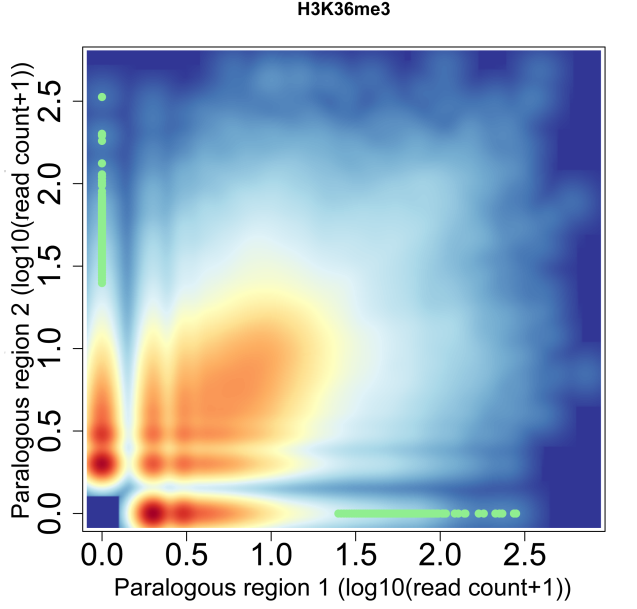


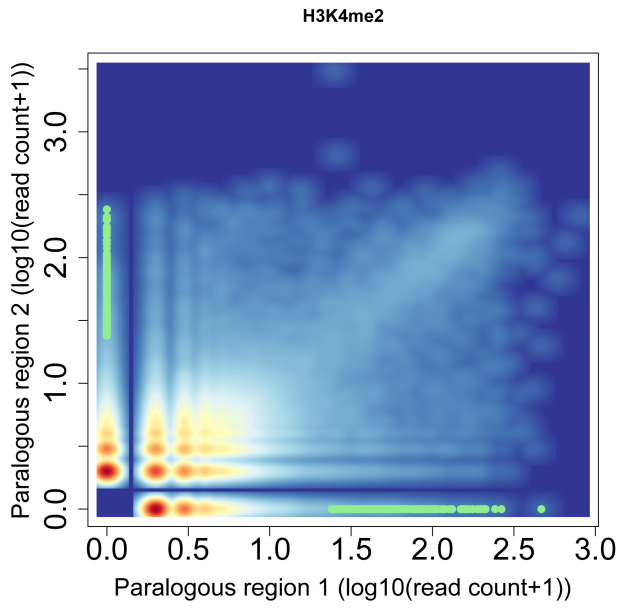

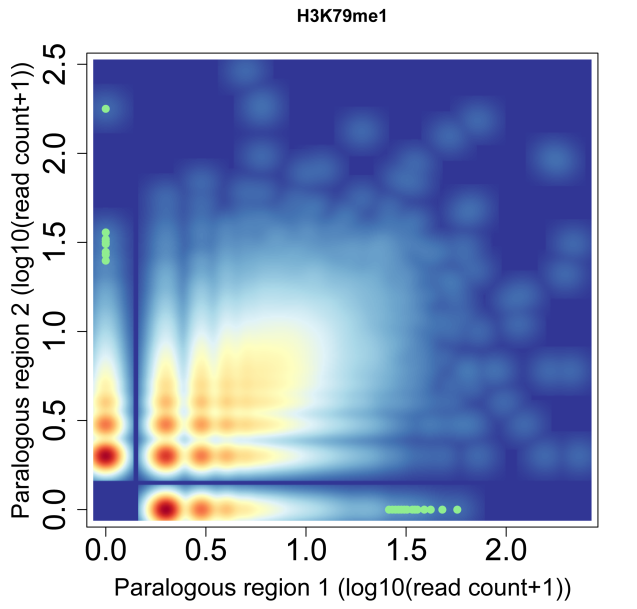


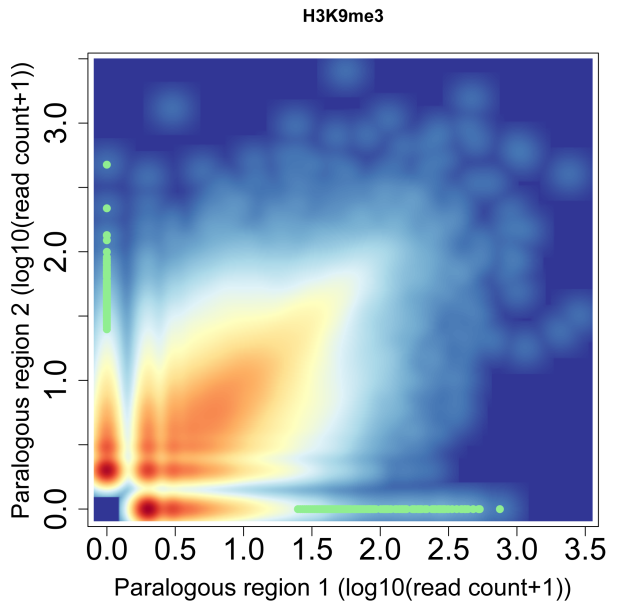

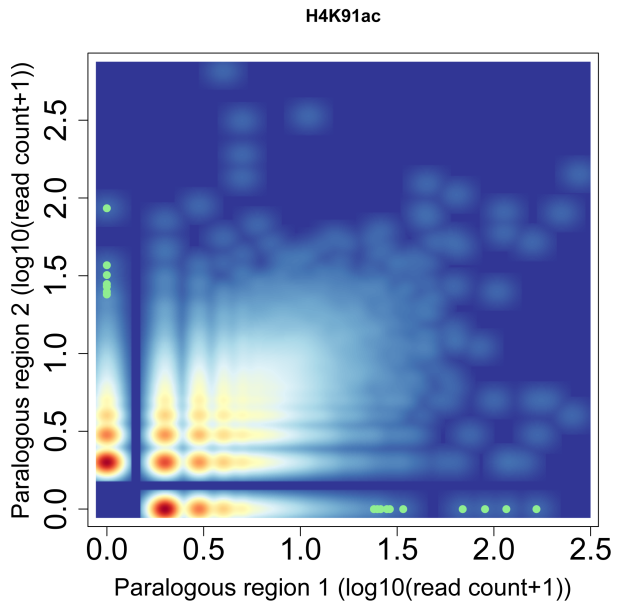


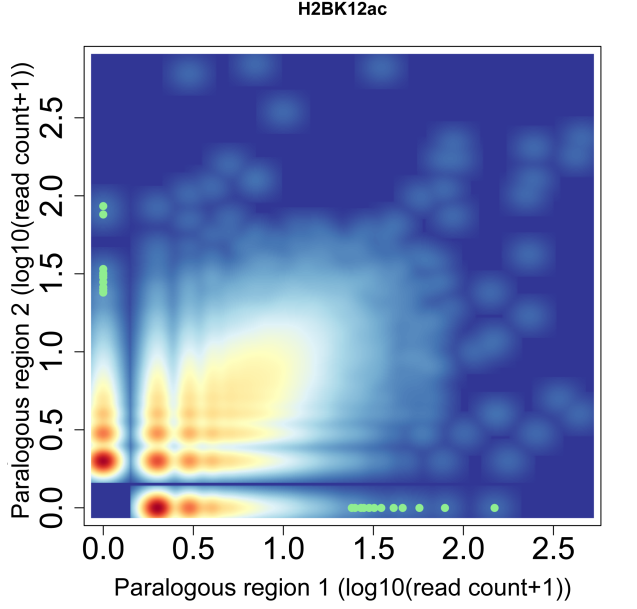

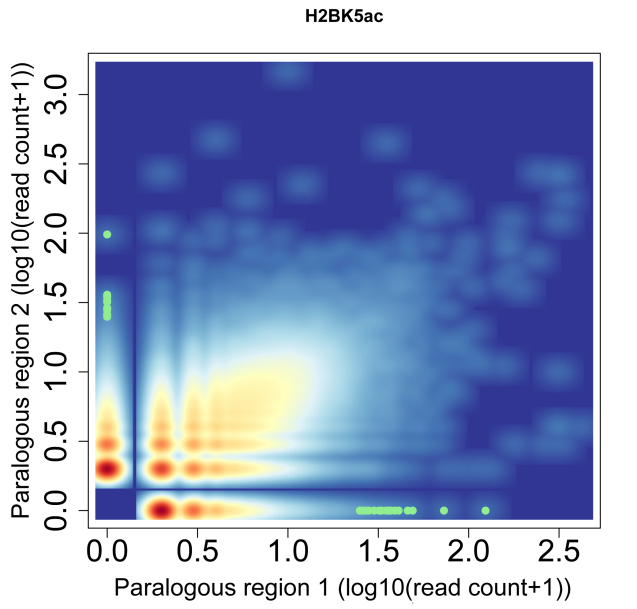


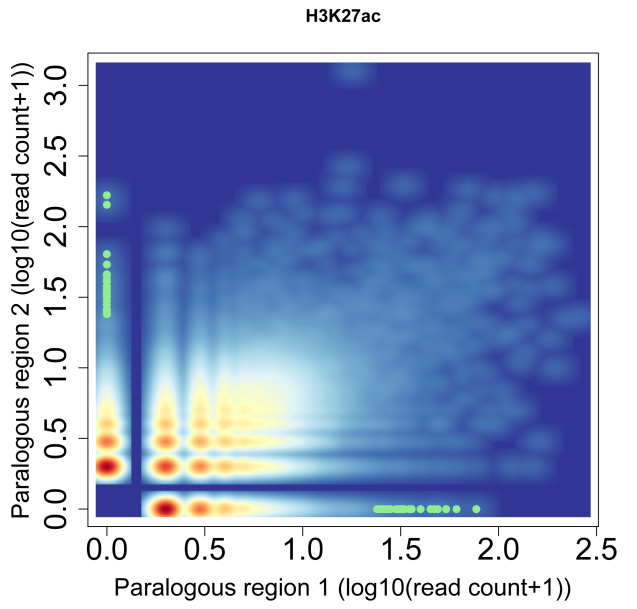

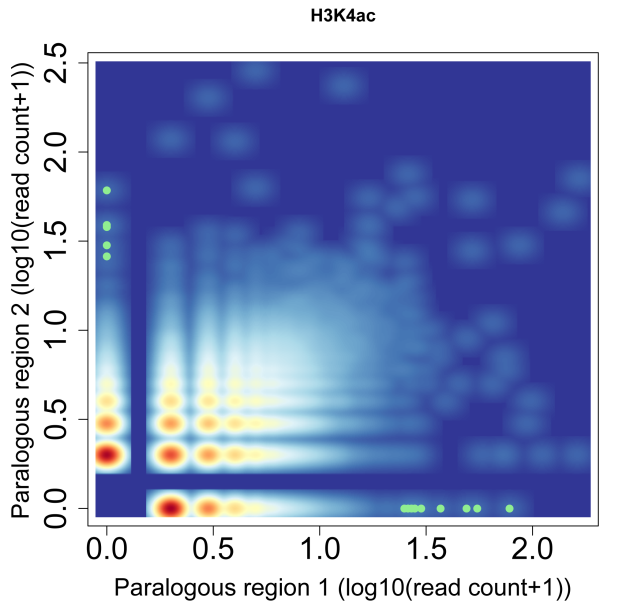


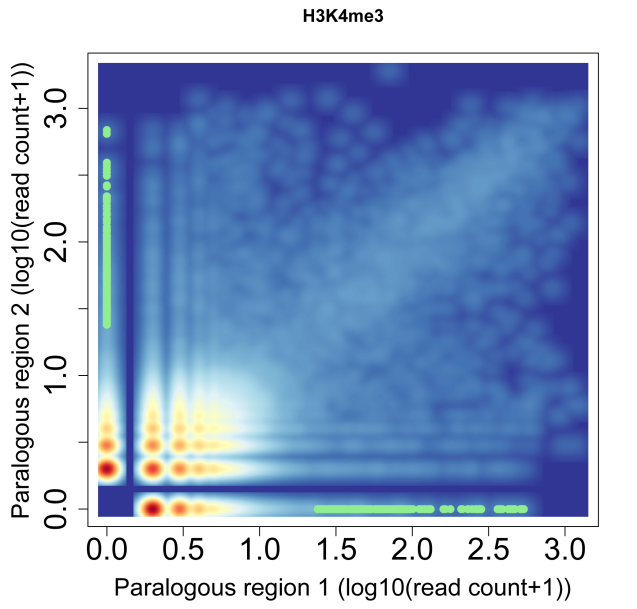

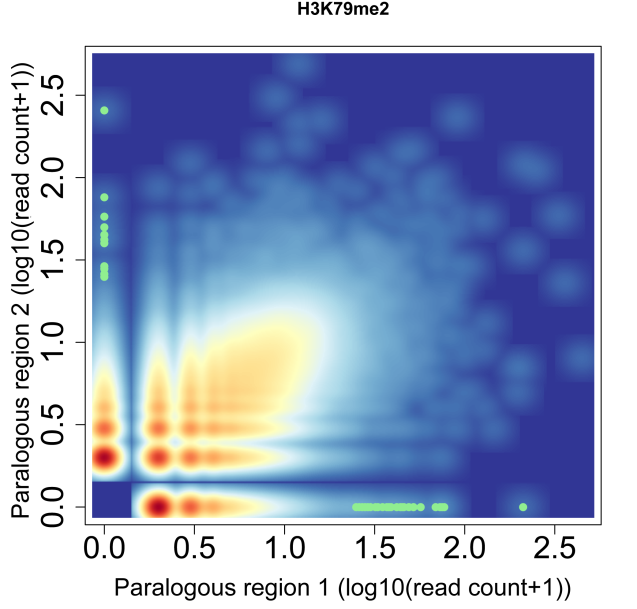


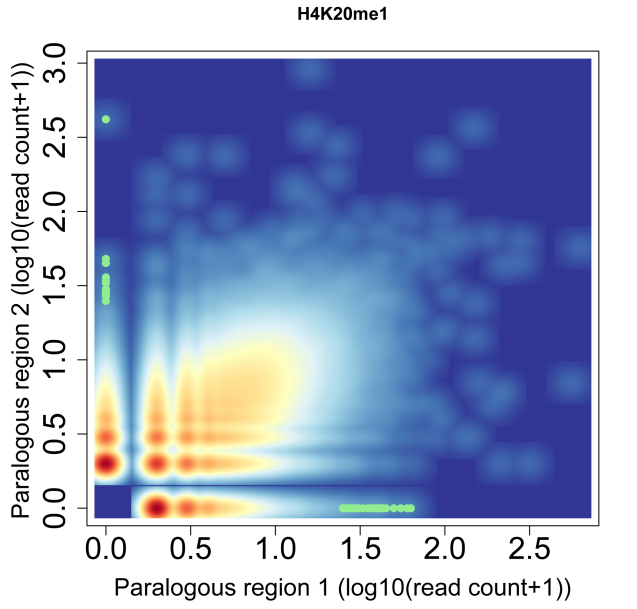

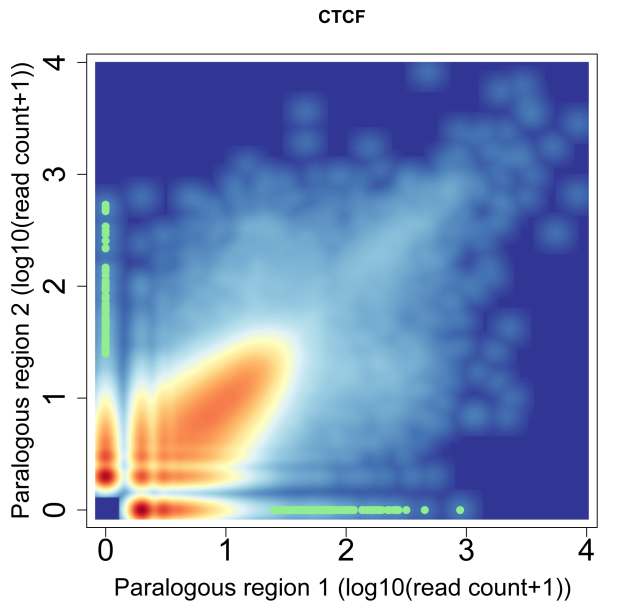


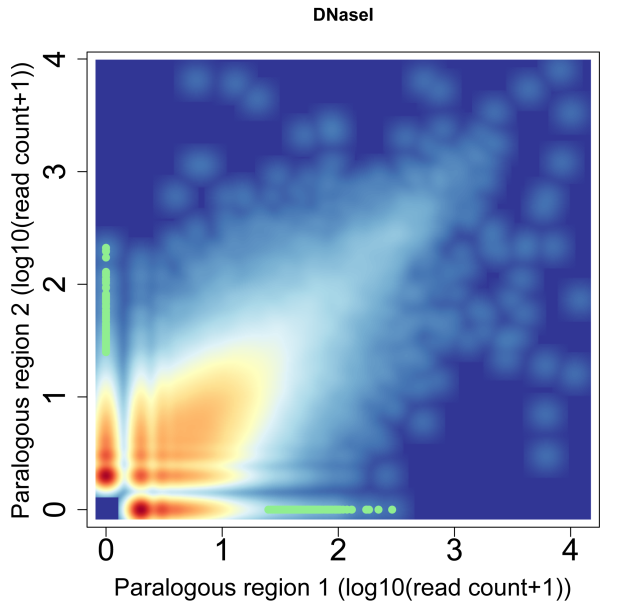


**Figure S3**: read counts of 25 ChIP-seq/DNase-seq experiments in the H1 cell line (corresponding to 23 histone modifications, DNase I and CTCF) found at each pair of non-overlapping 500bp paralogous regions in the human genome. Paralogous pairs of windows with no reads mapping to either region are excluded from these plots. Pairs of paralogous regions of significantly different chromatin states (Bonferroni corrected binomial p value < 0.05) and with one of the regions having no corresponding reads mapping to it are shown in light green (these being the pairs of regions subsequently analysed to determine if DNA motifs are linked to chromatin divergence). log10(read count+1) values of 0, 1, 2 and 3 correspond to read counts of 0, 9, 99 and 999 respectively.


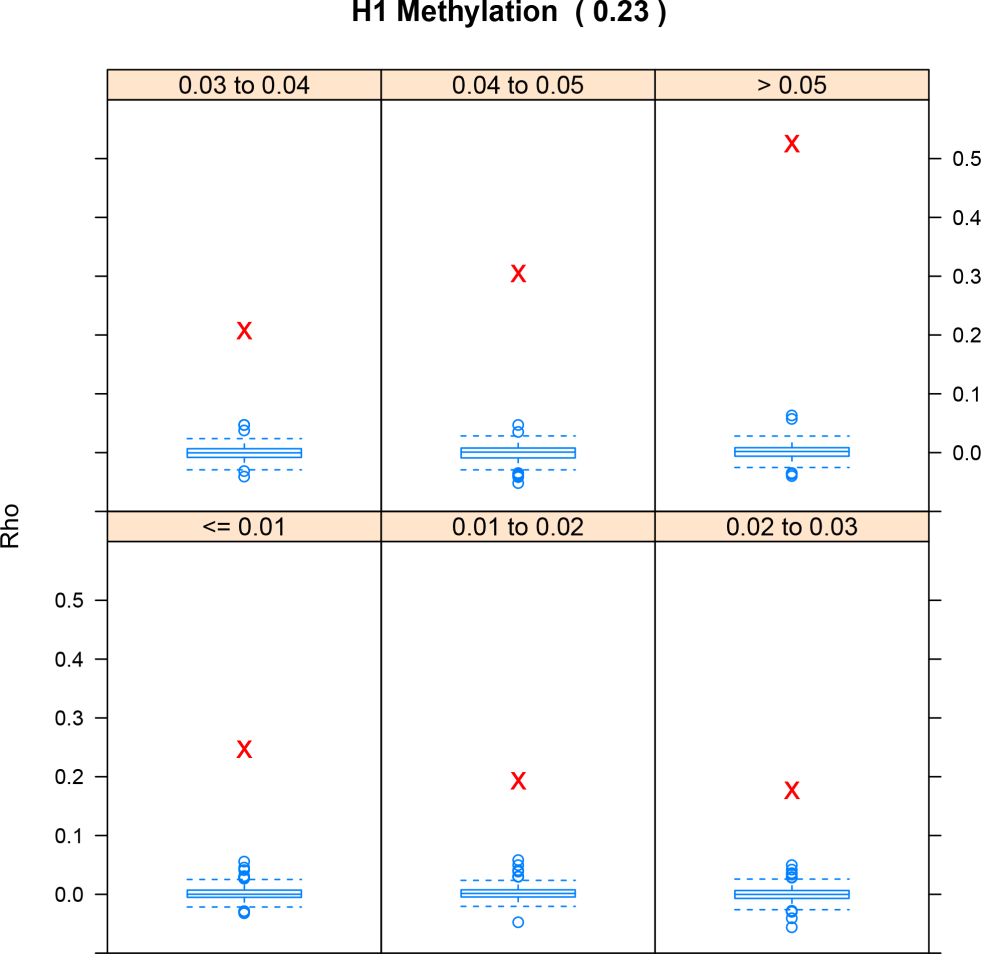


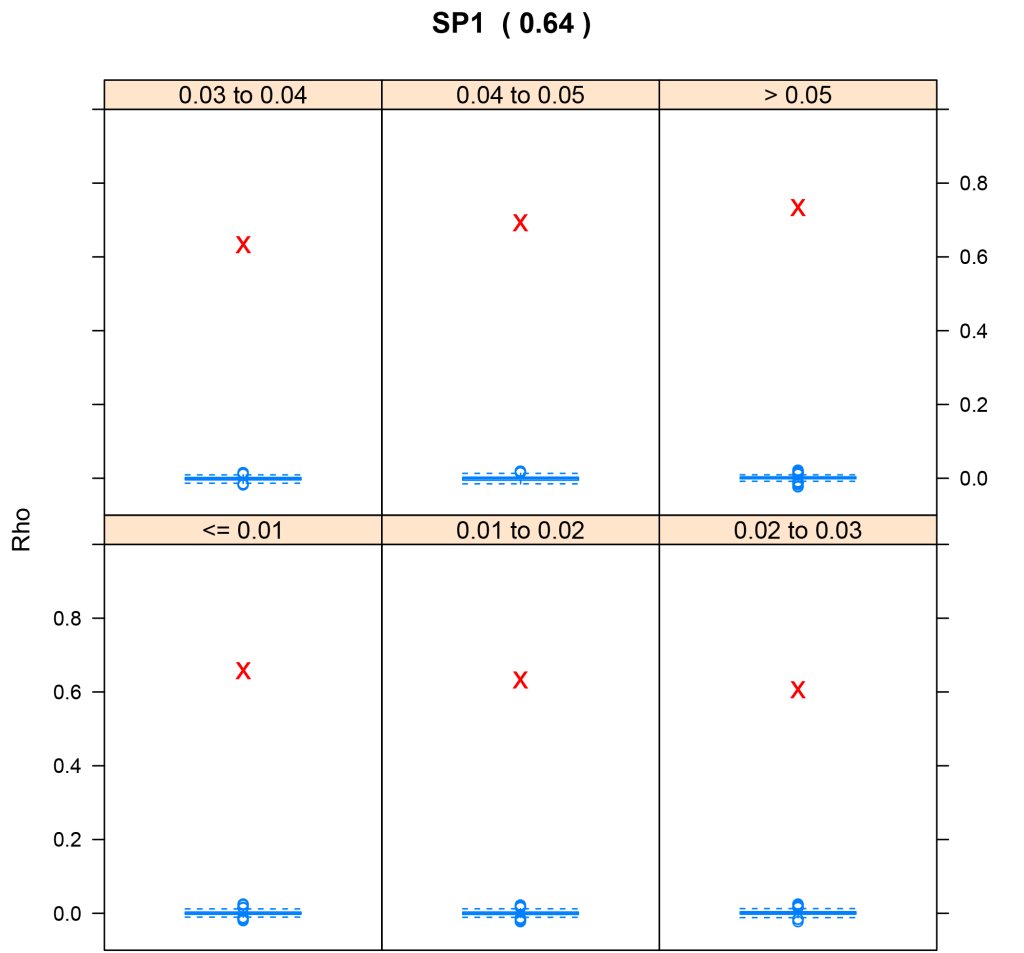

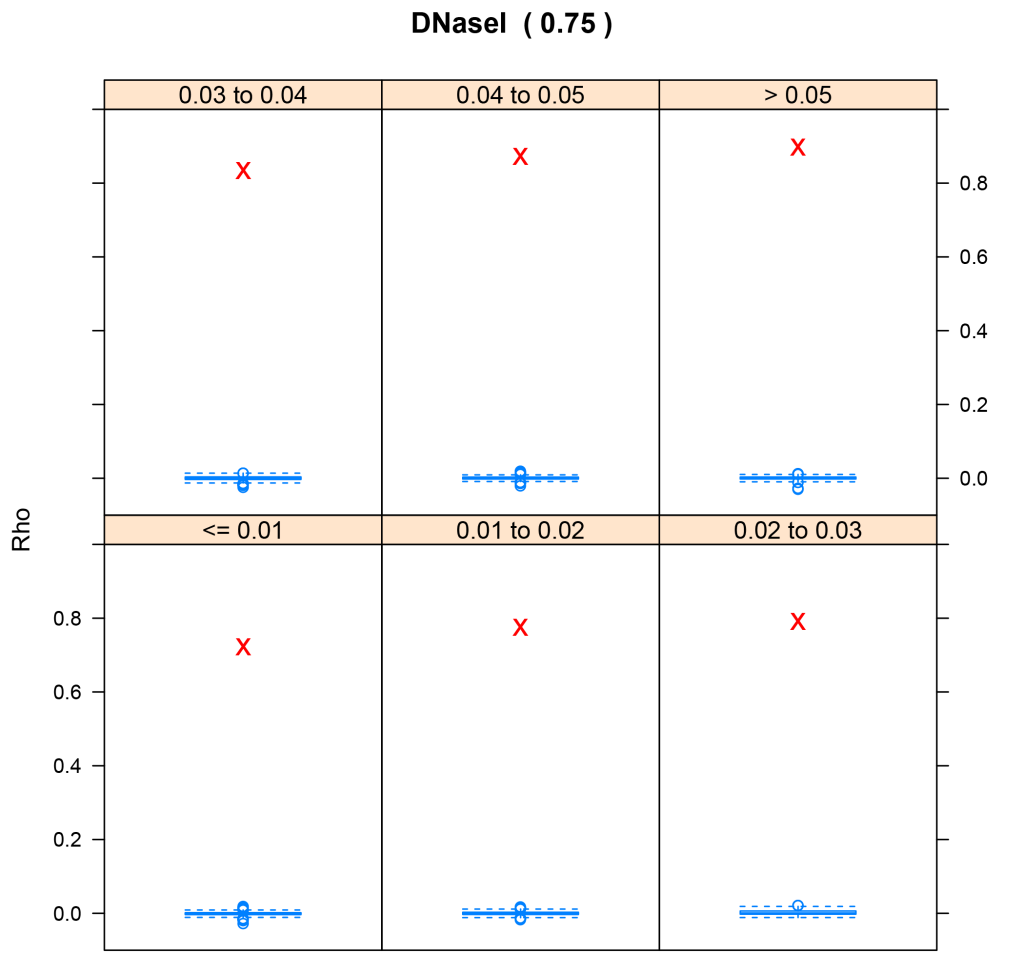

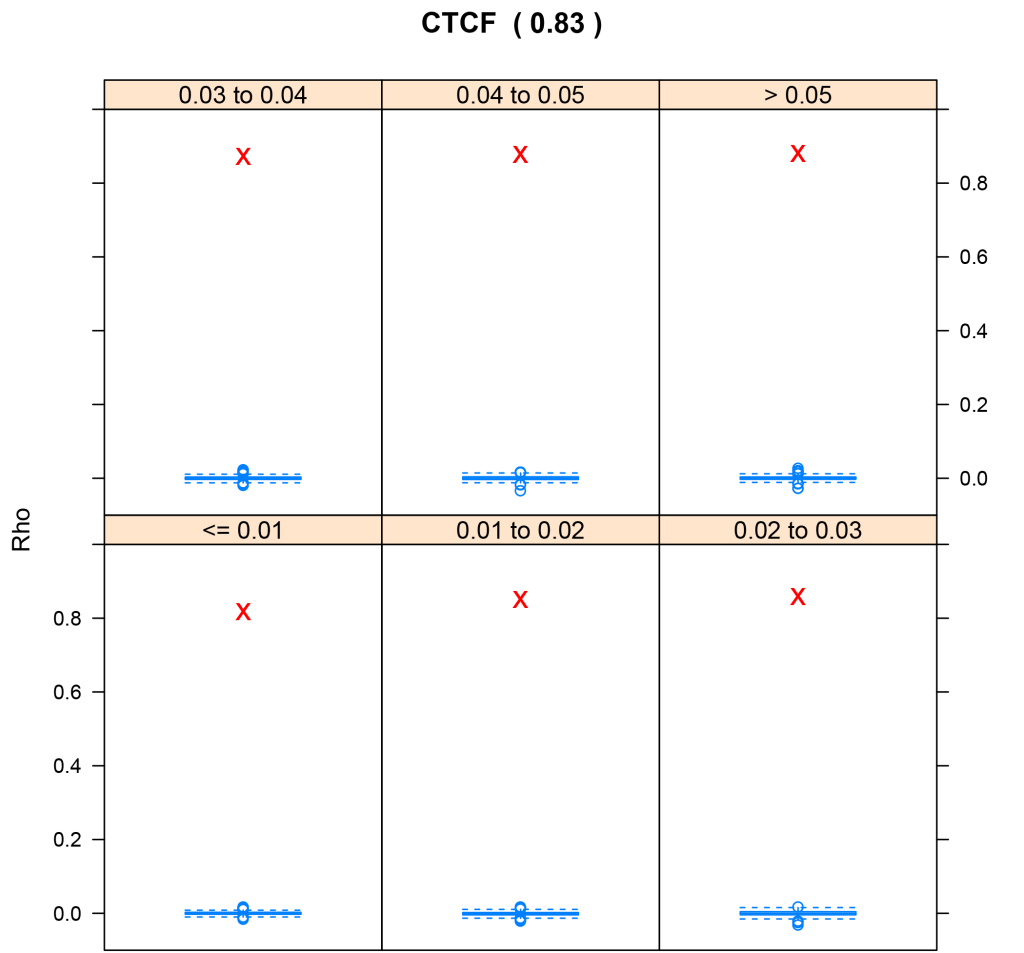

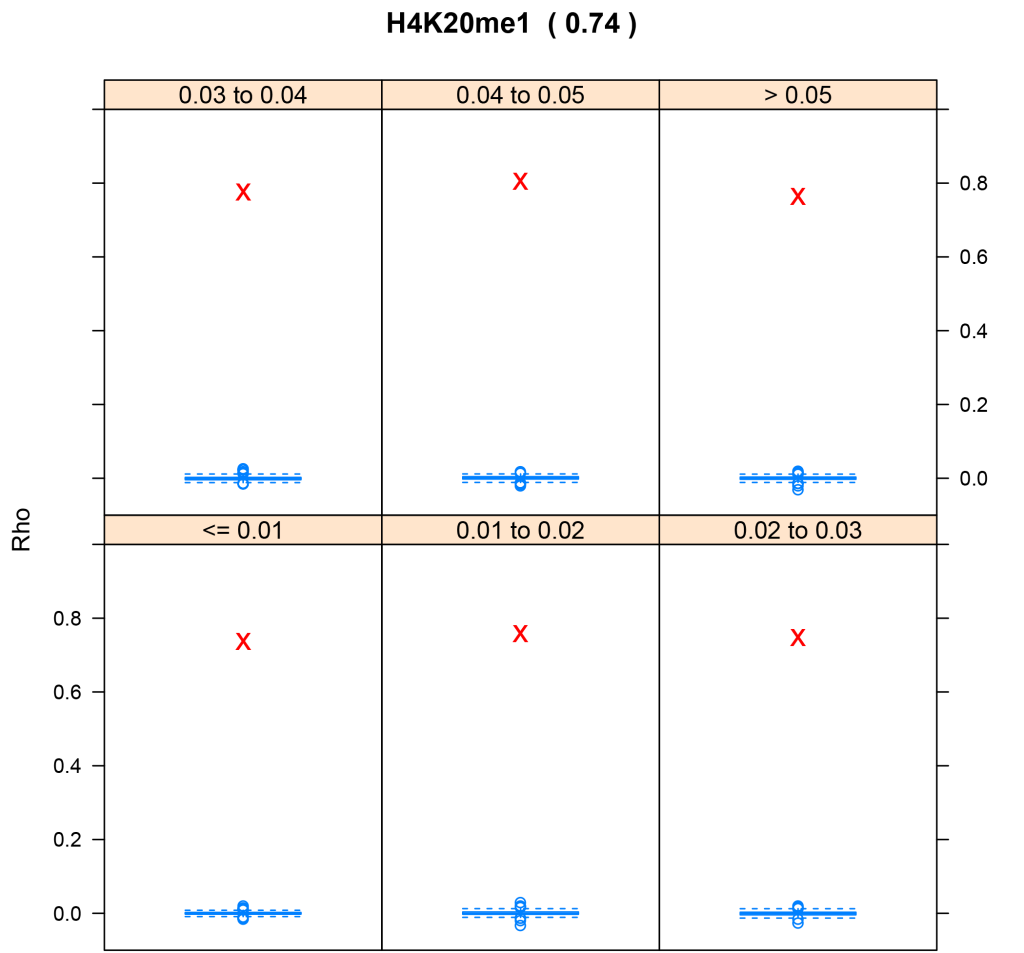


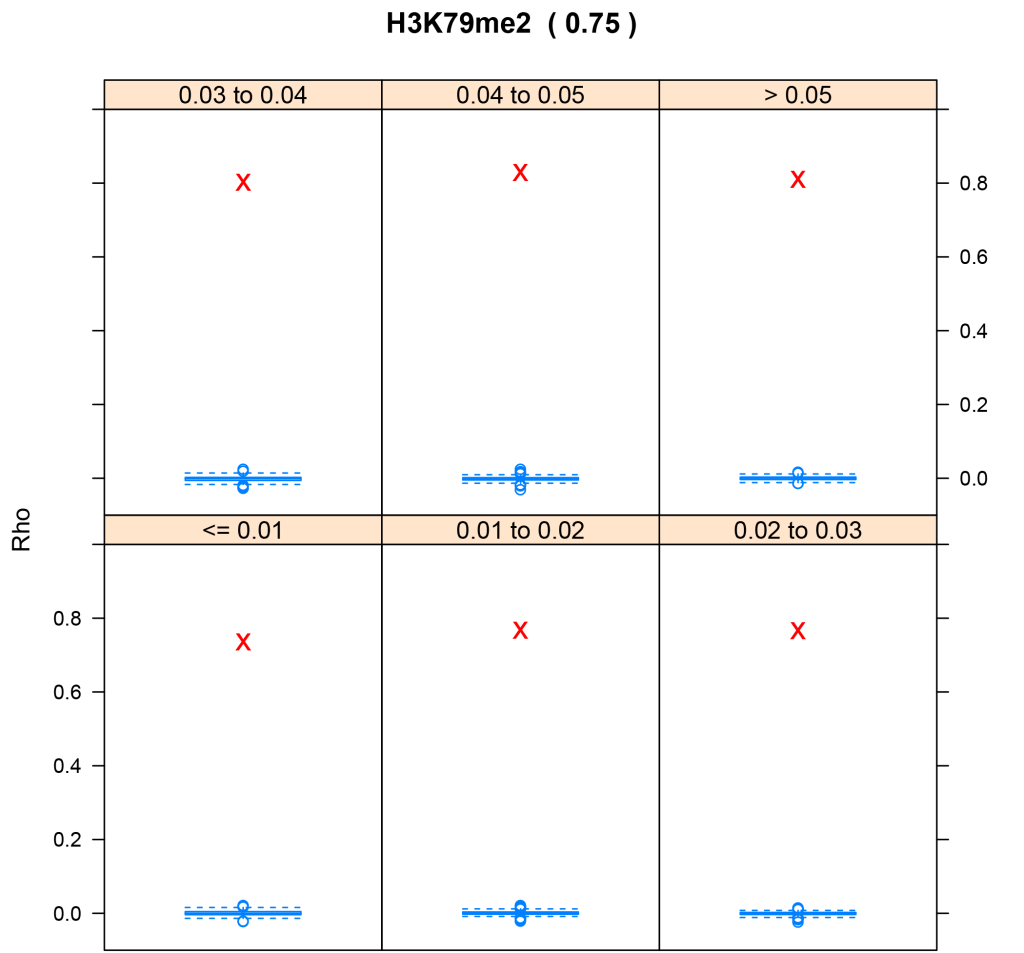

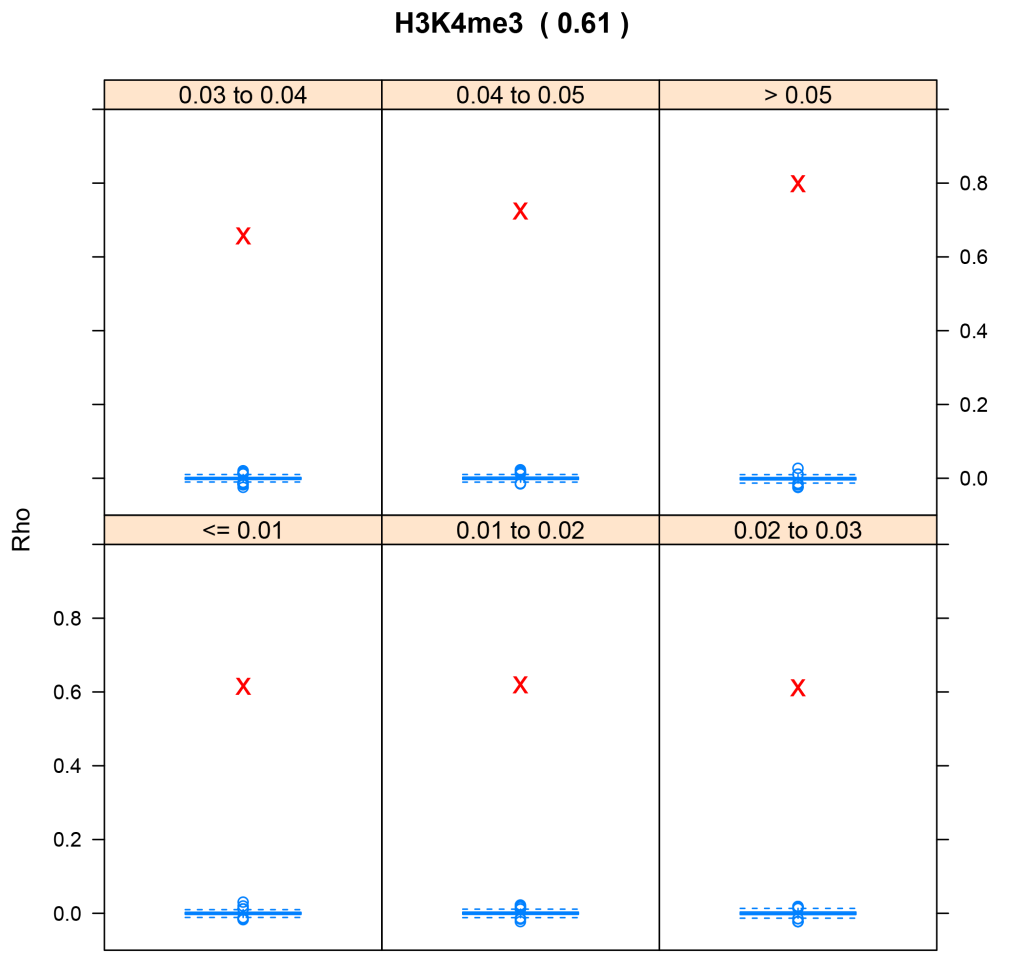

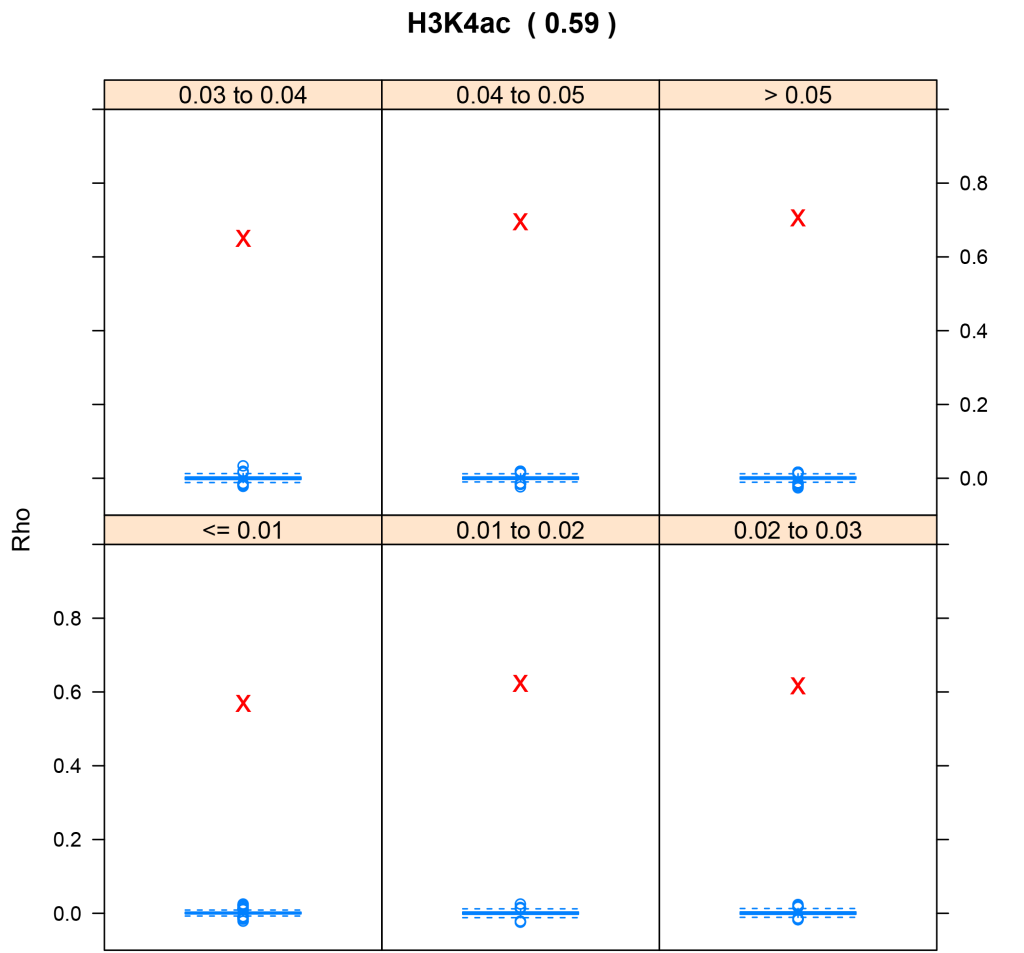

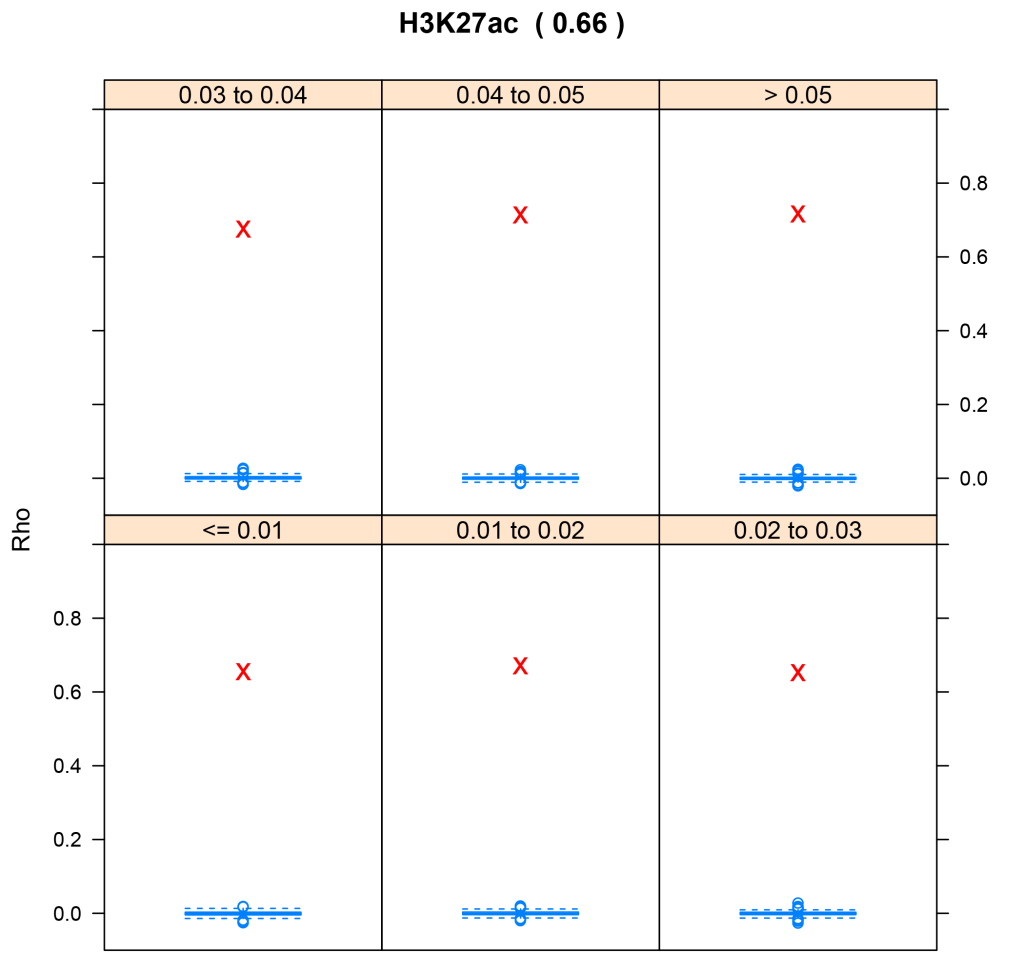

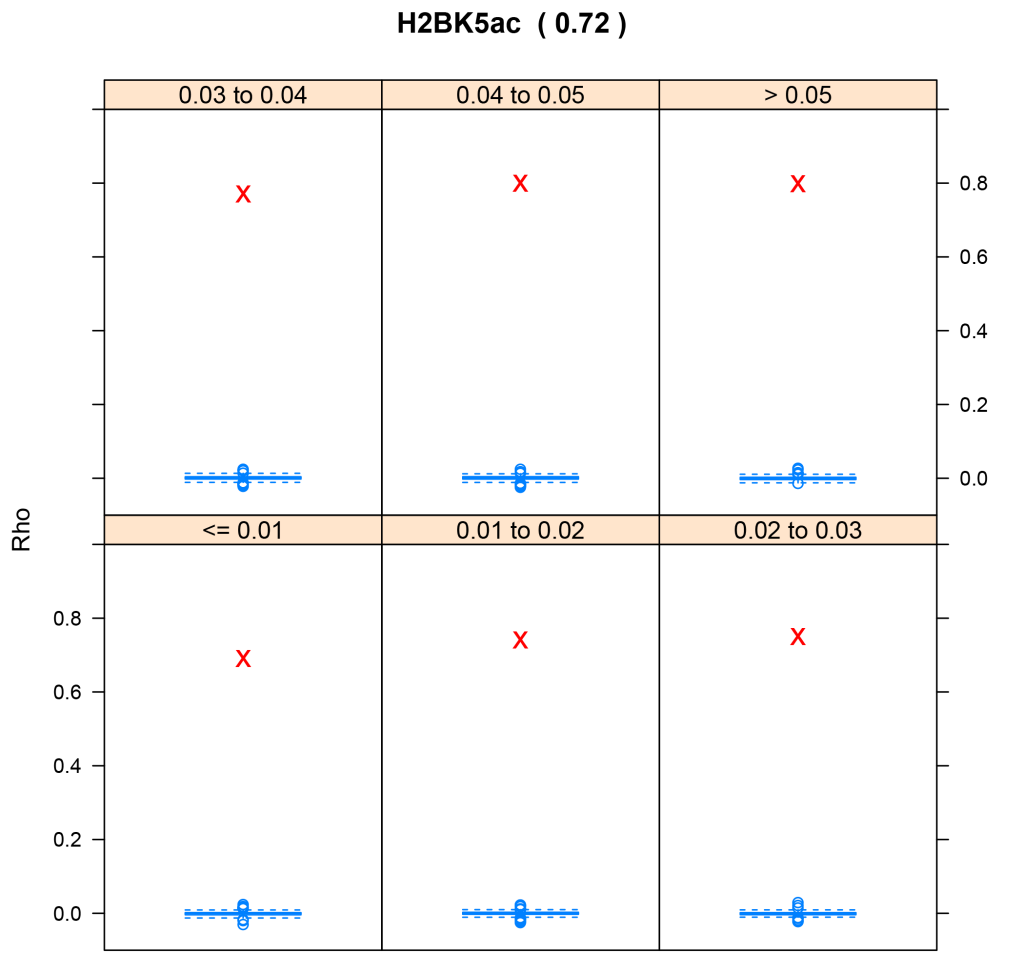

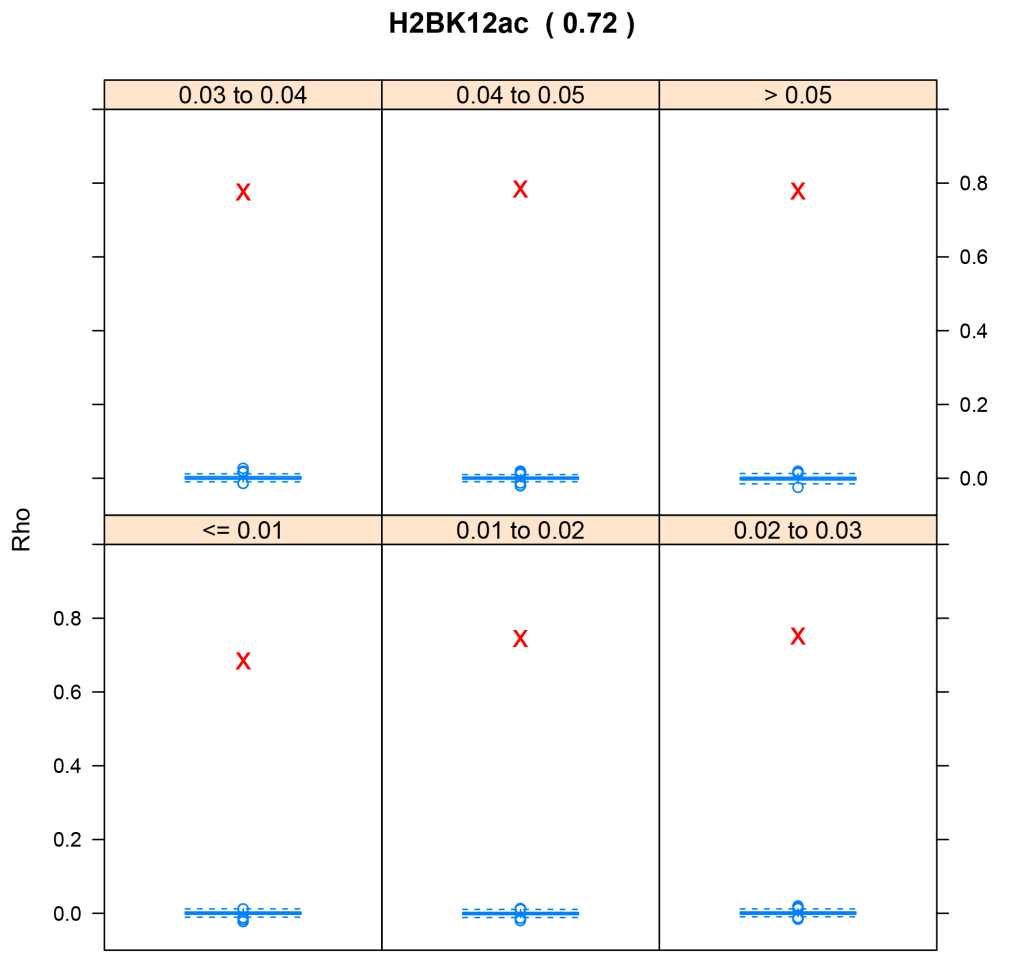

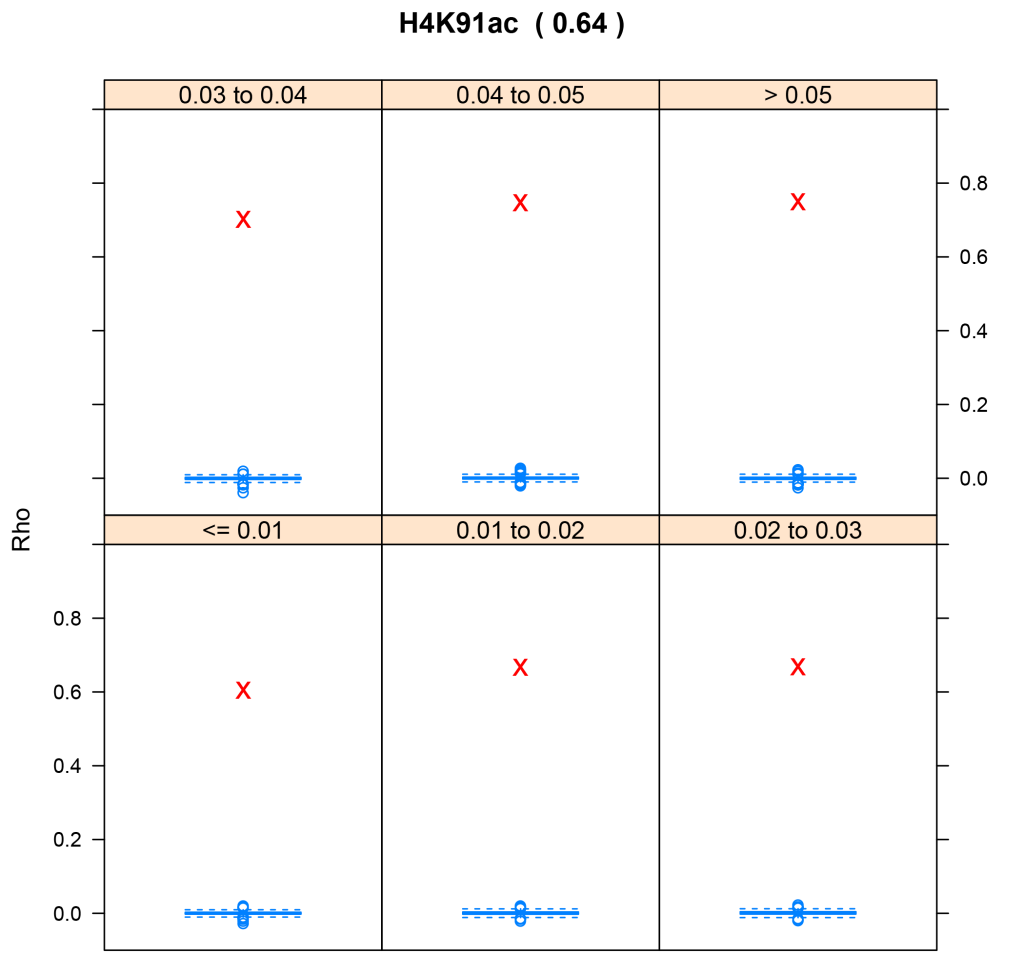

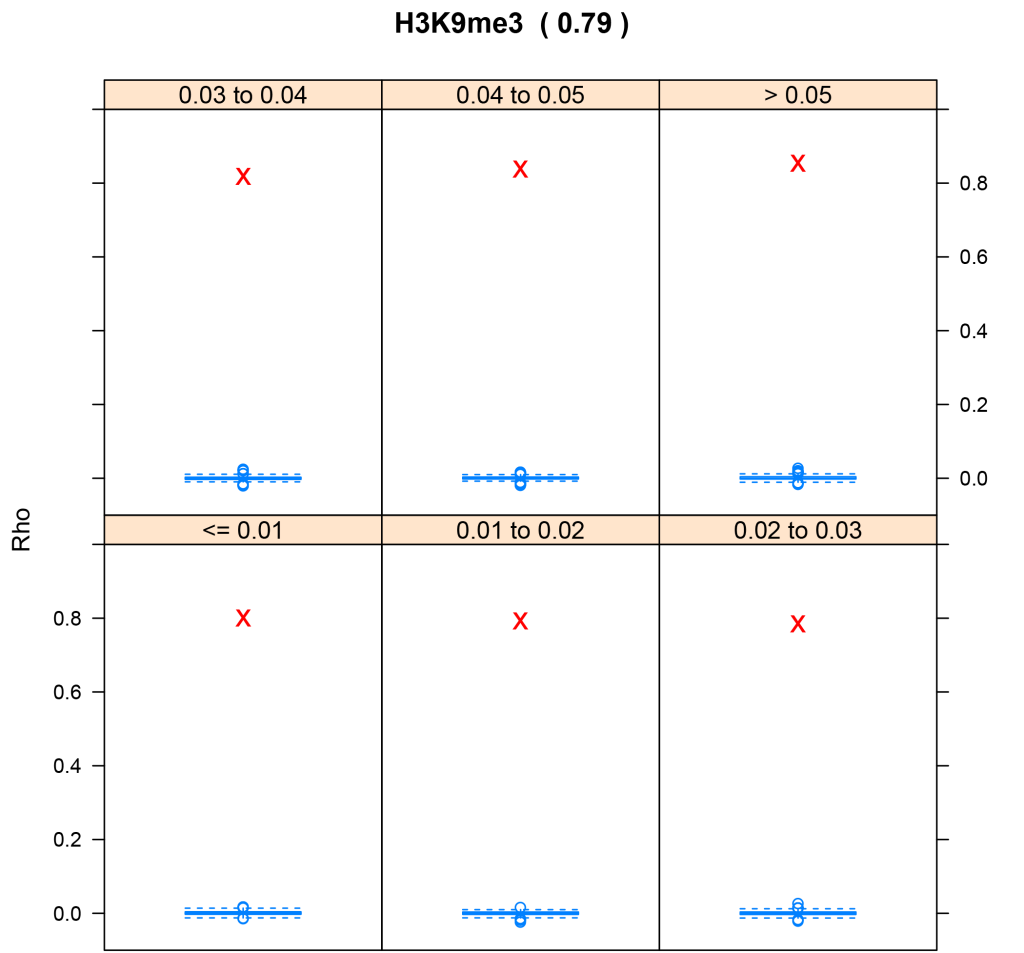

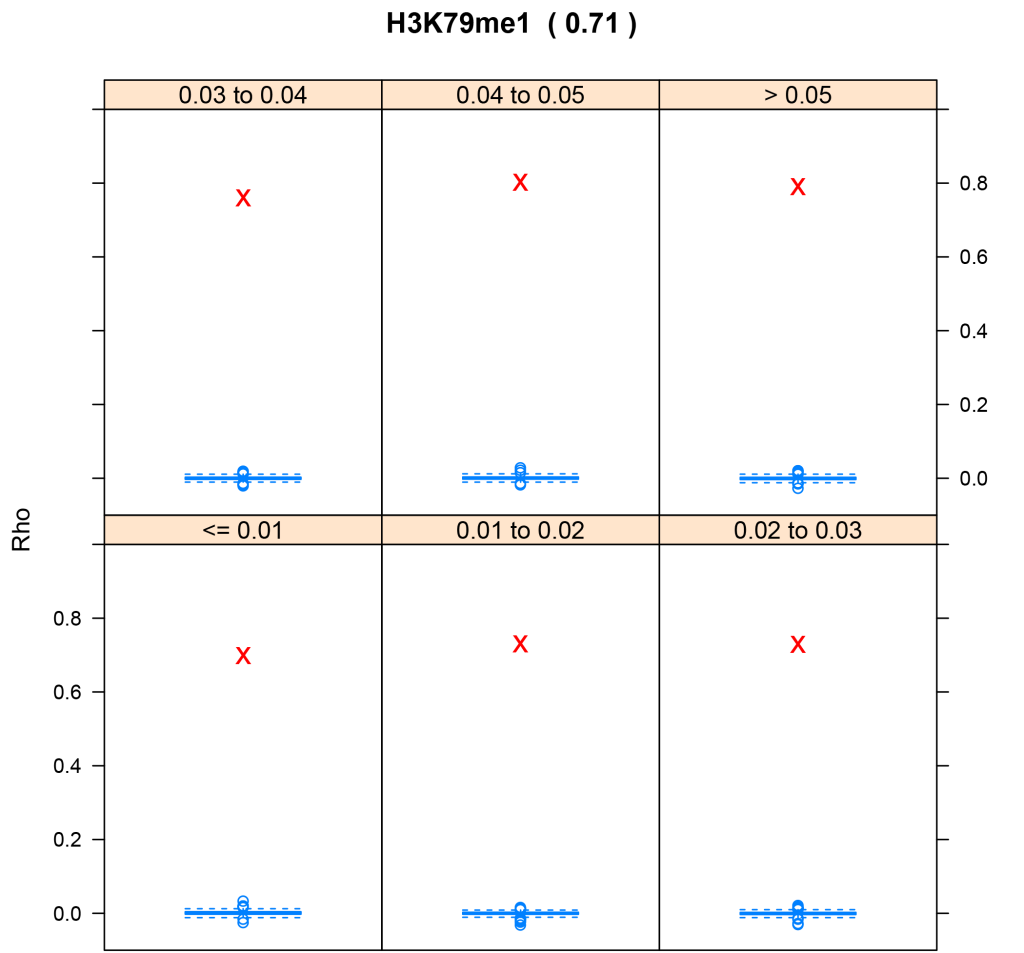

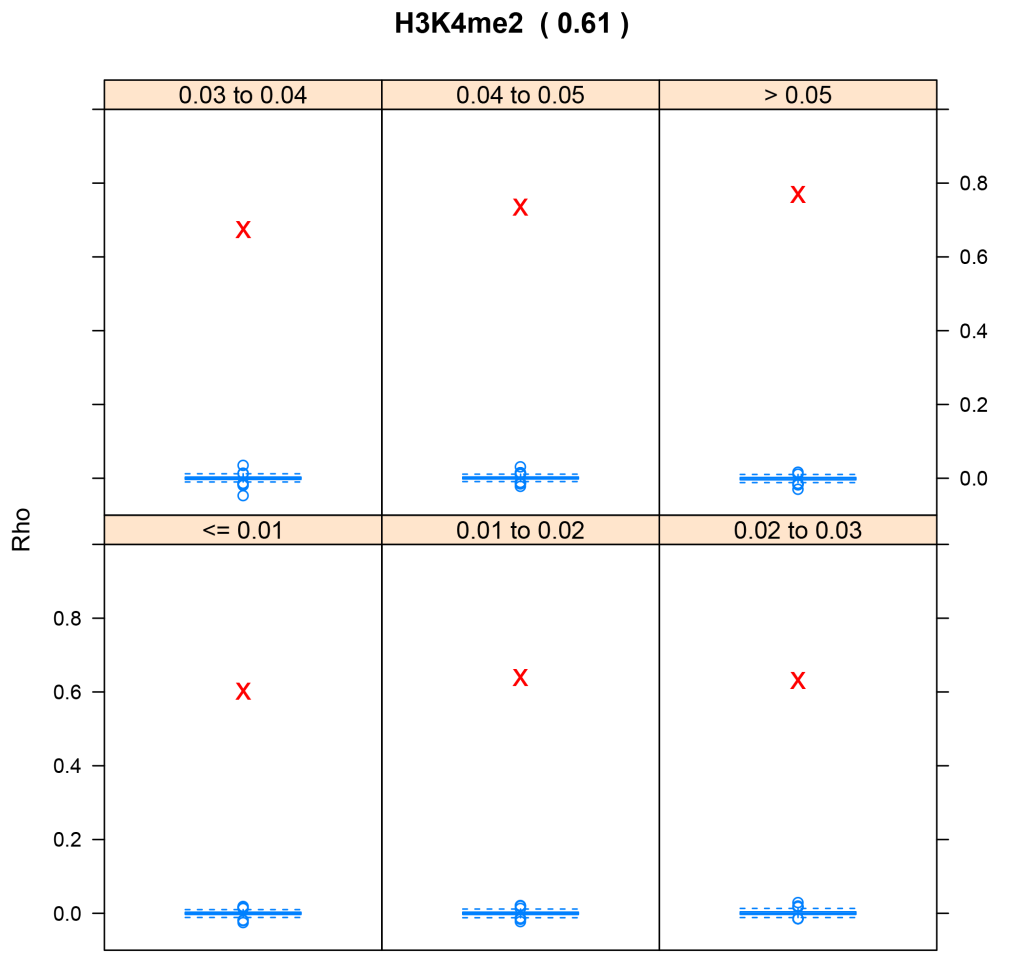

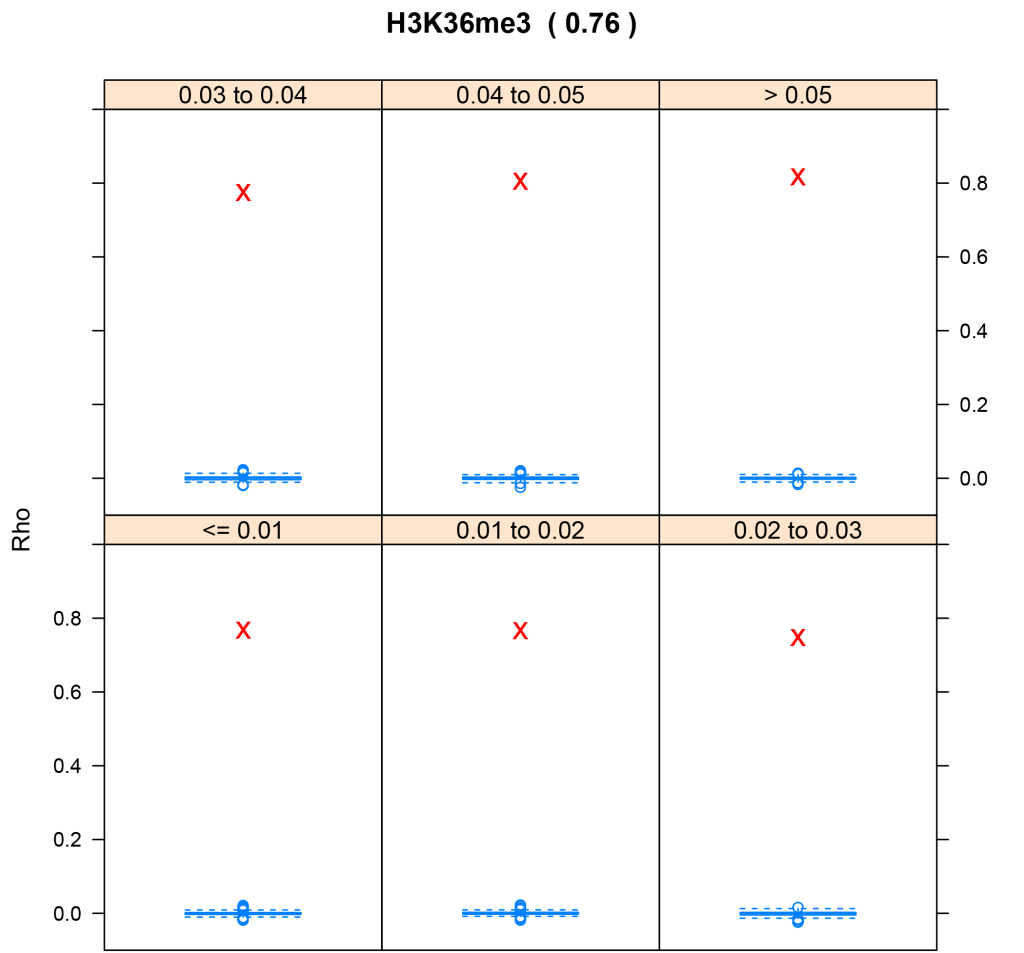

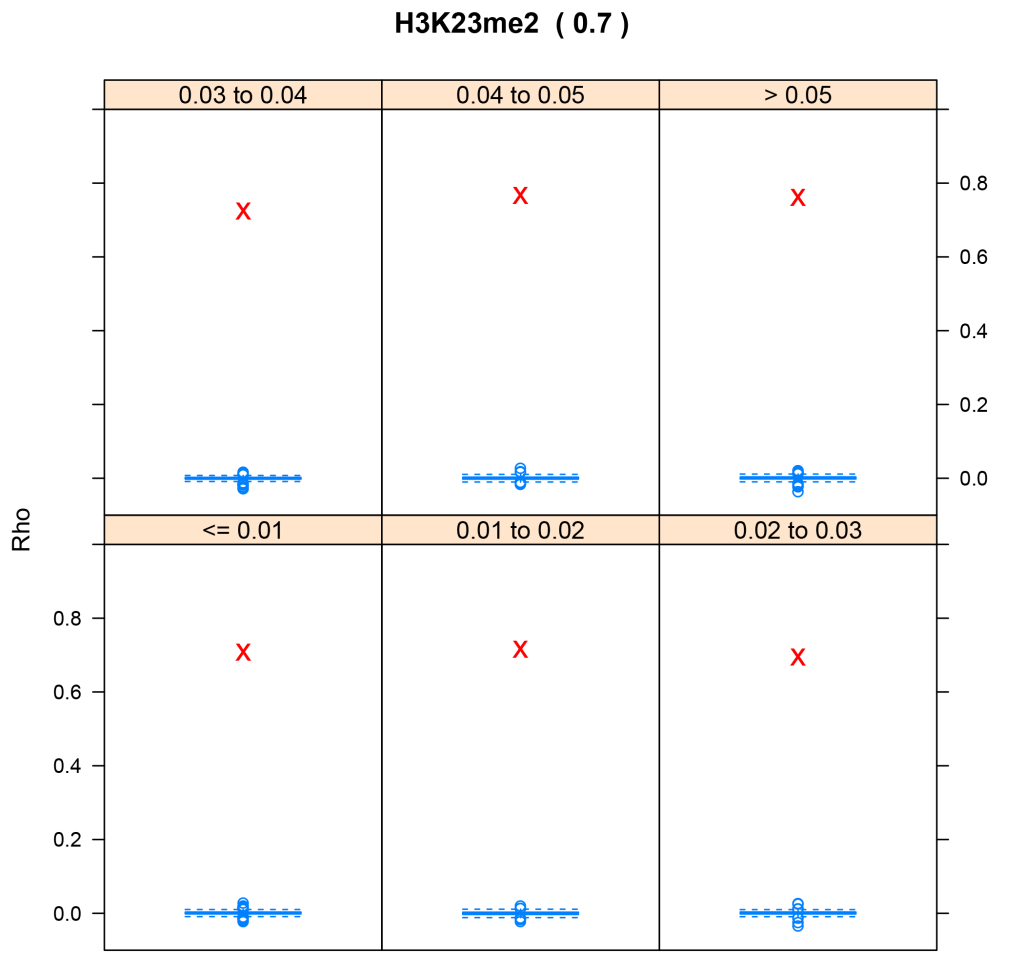

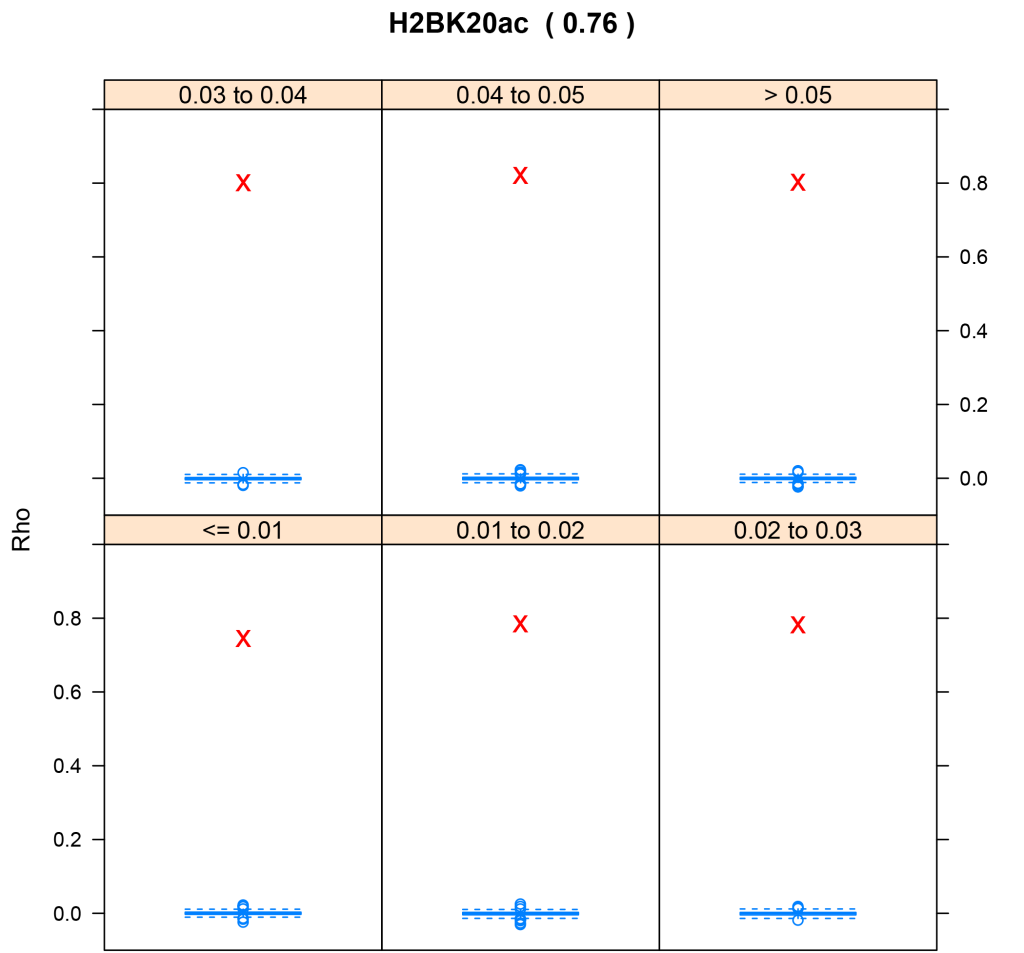

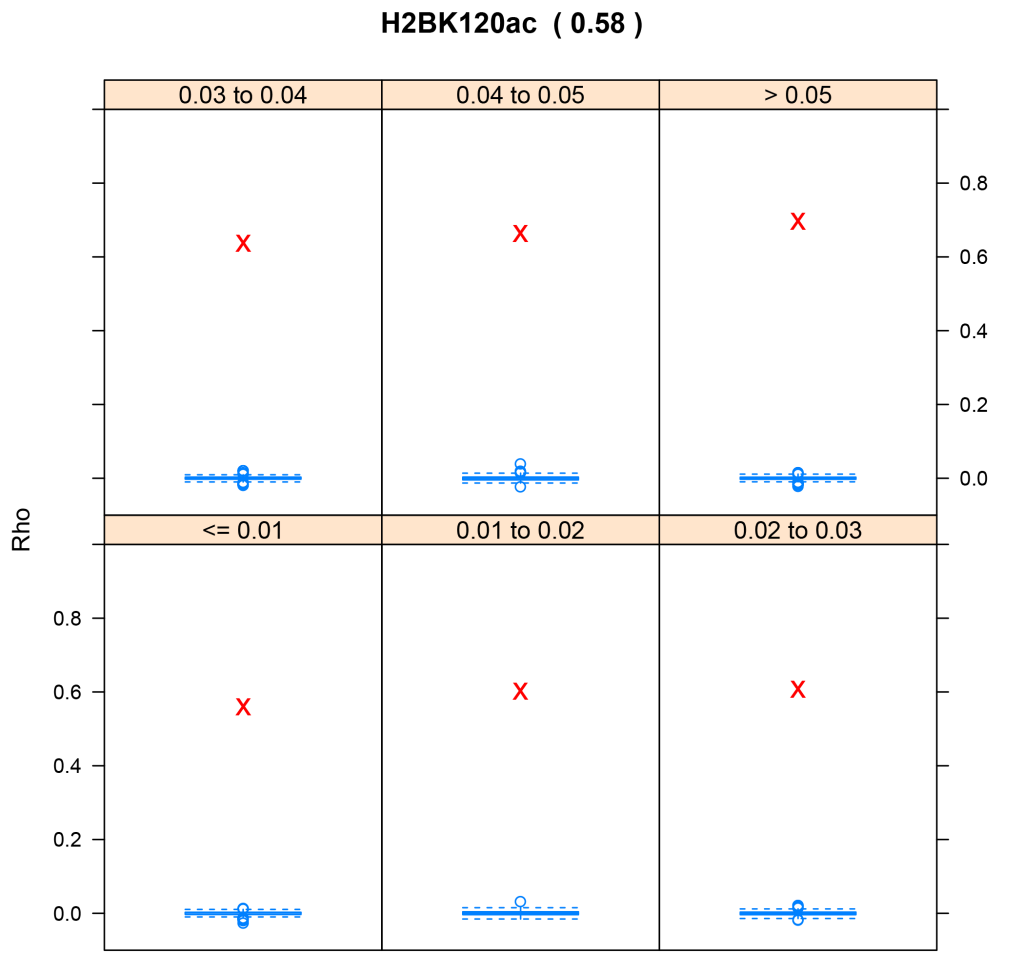

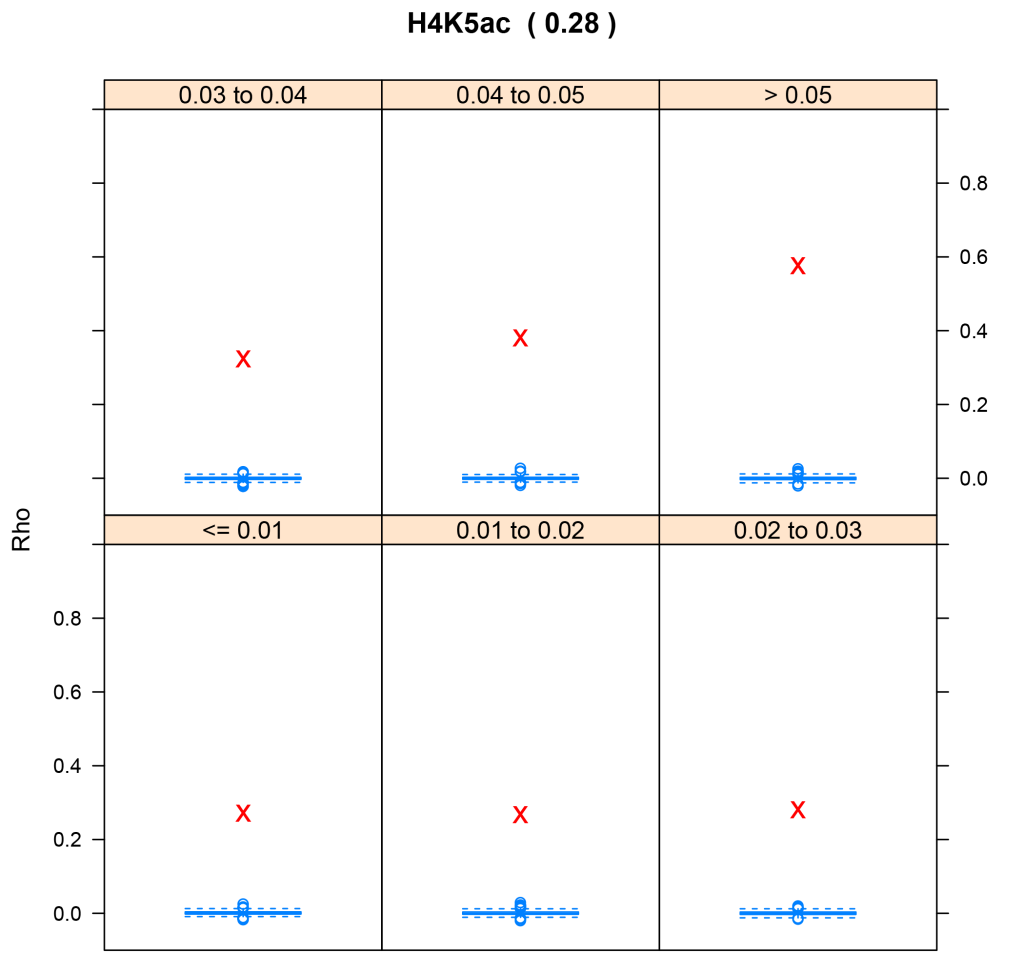

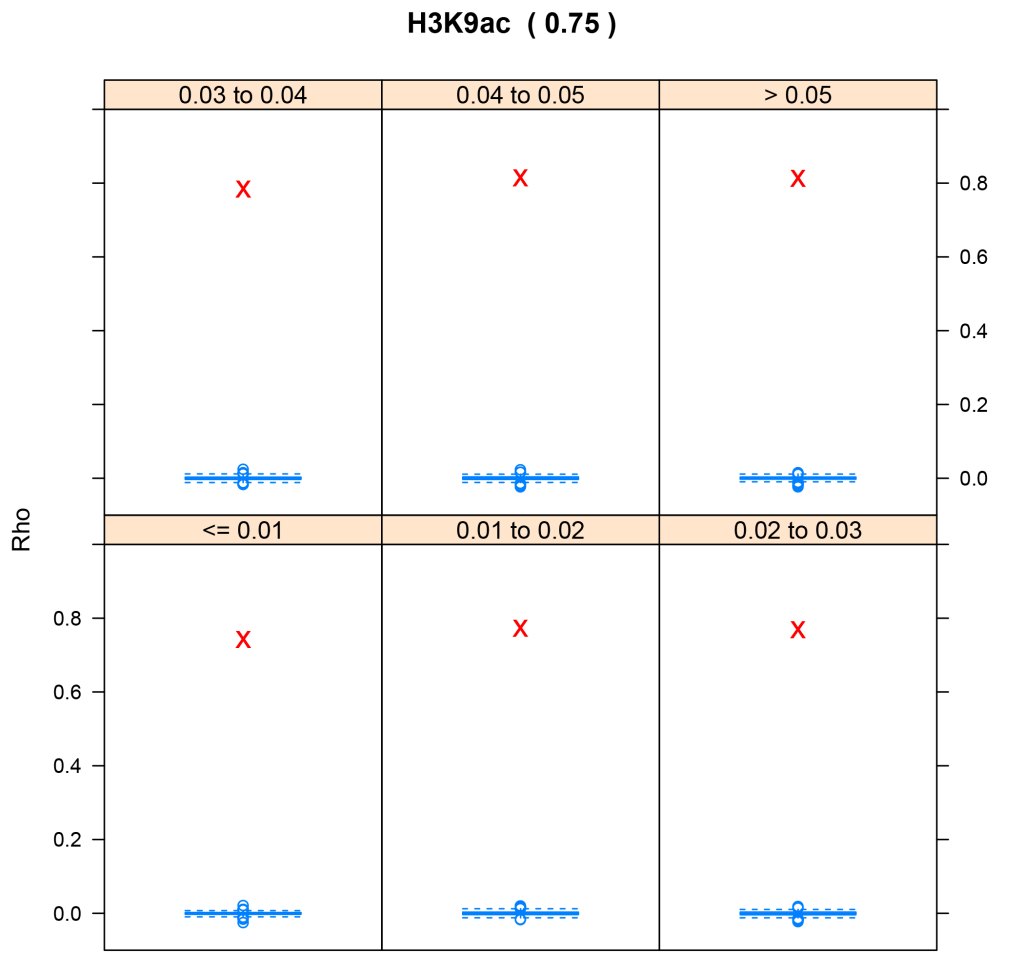

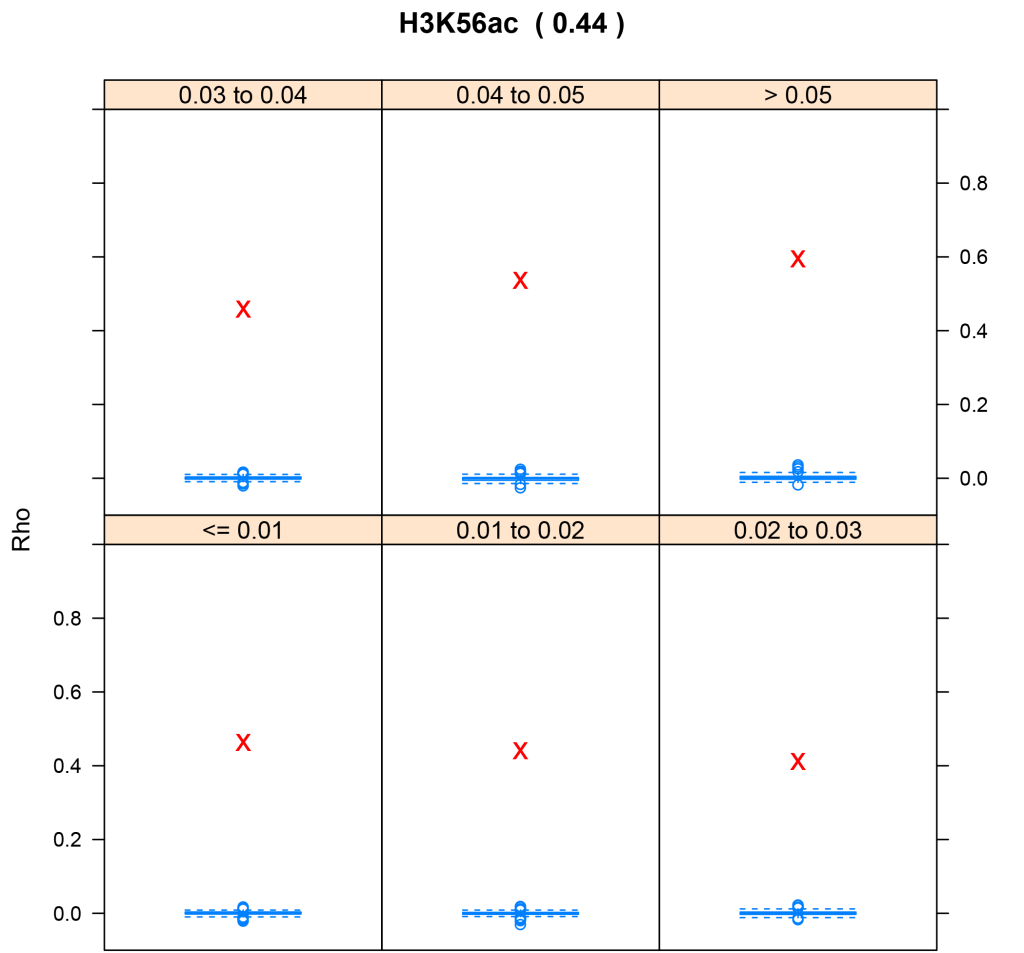

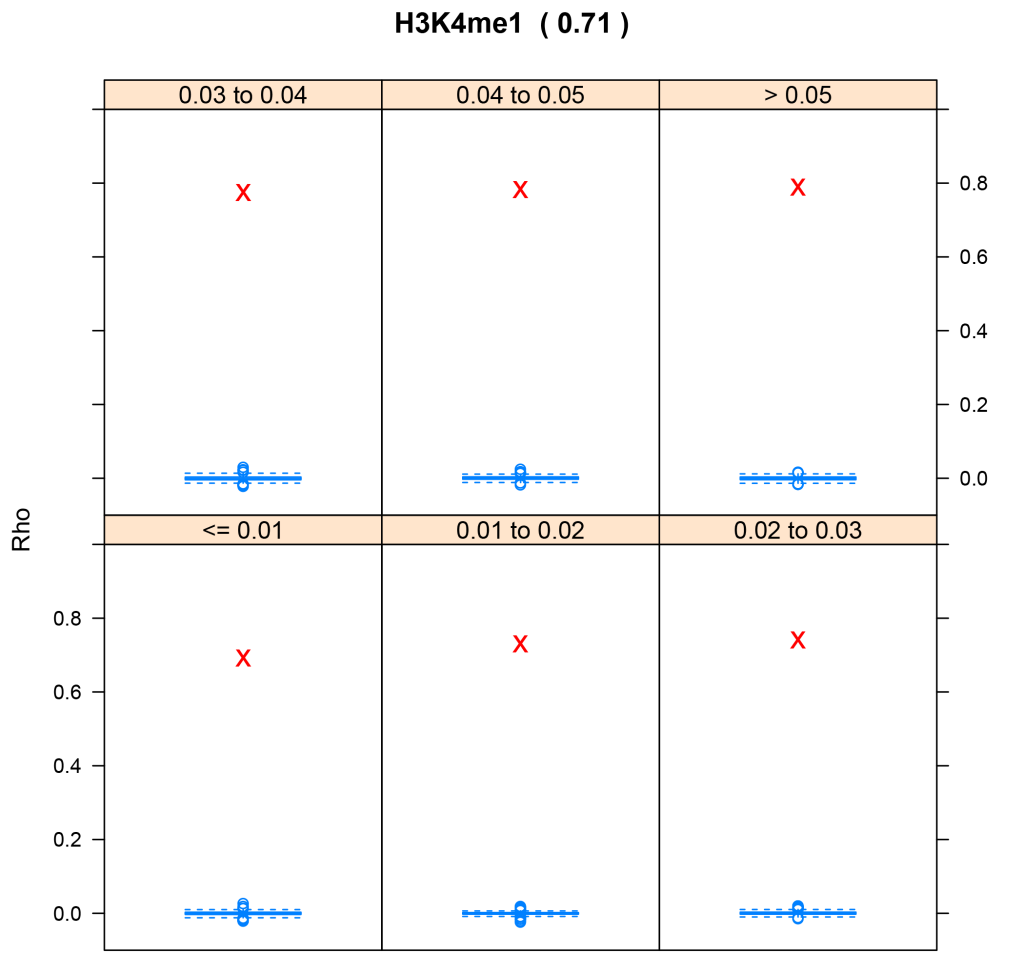

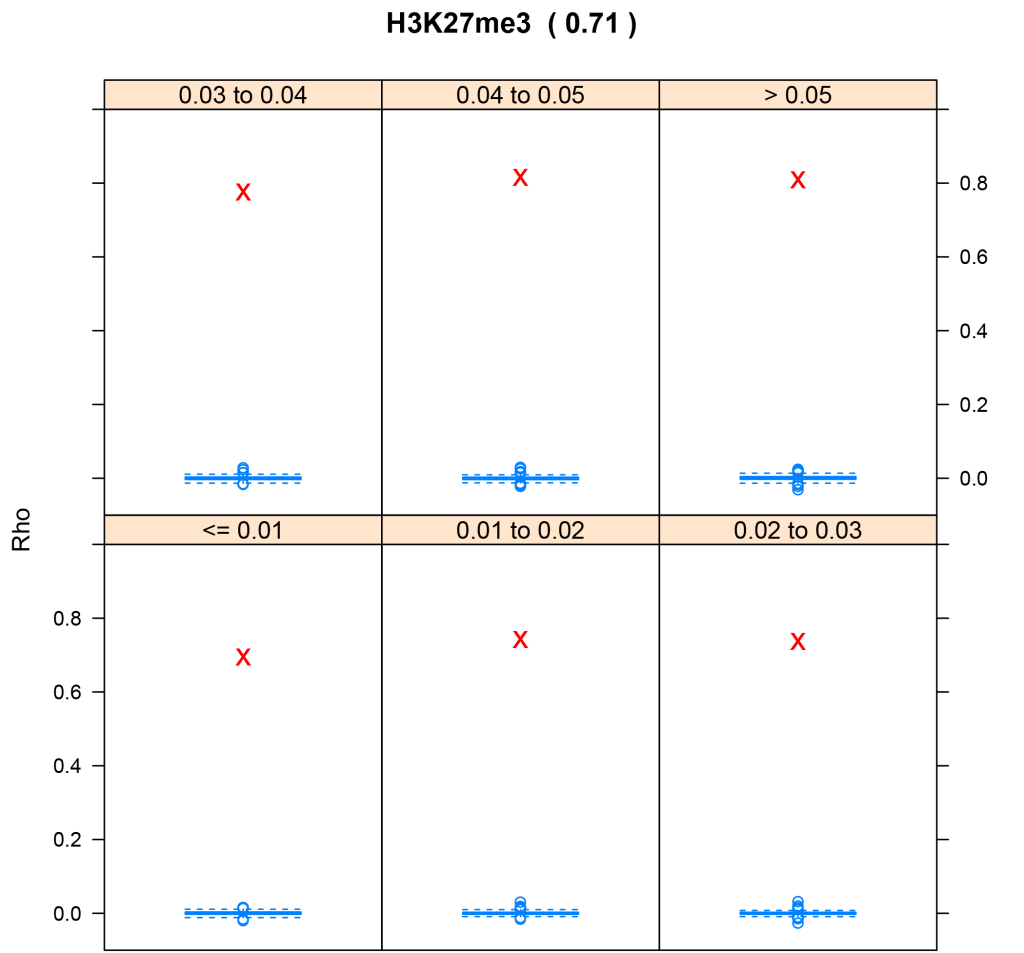

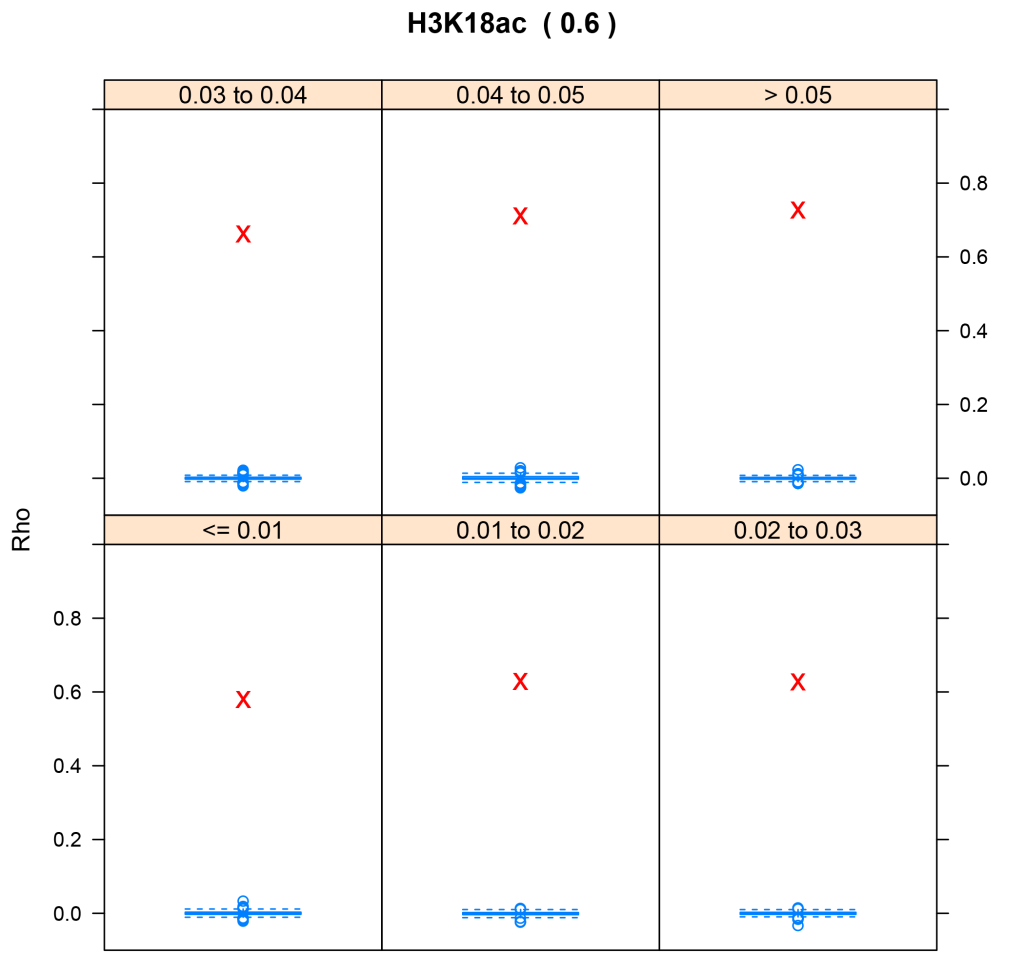


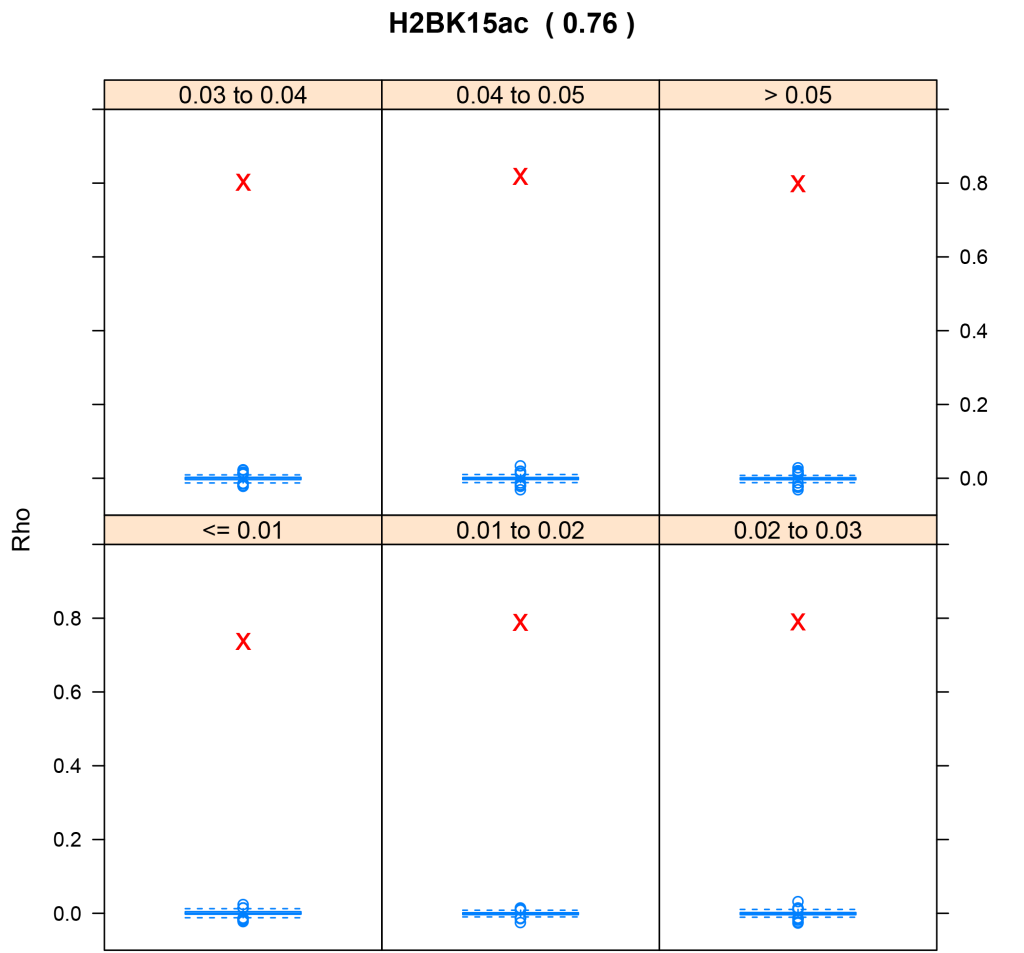


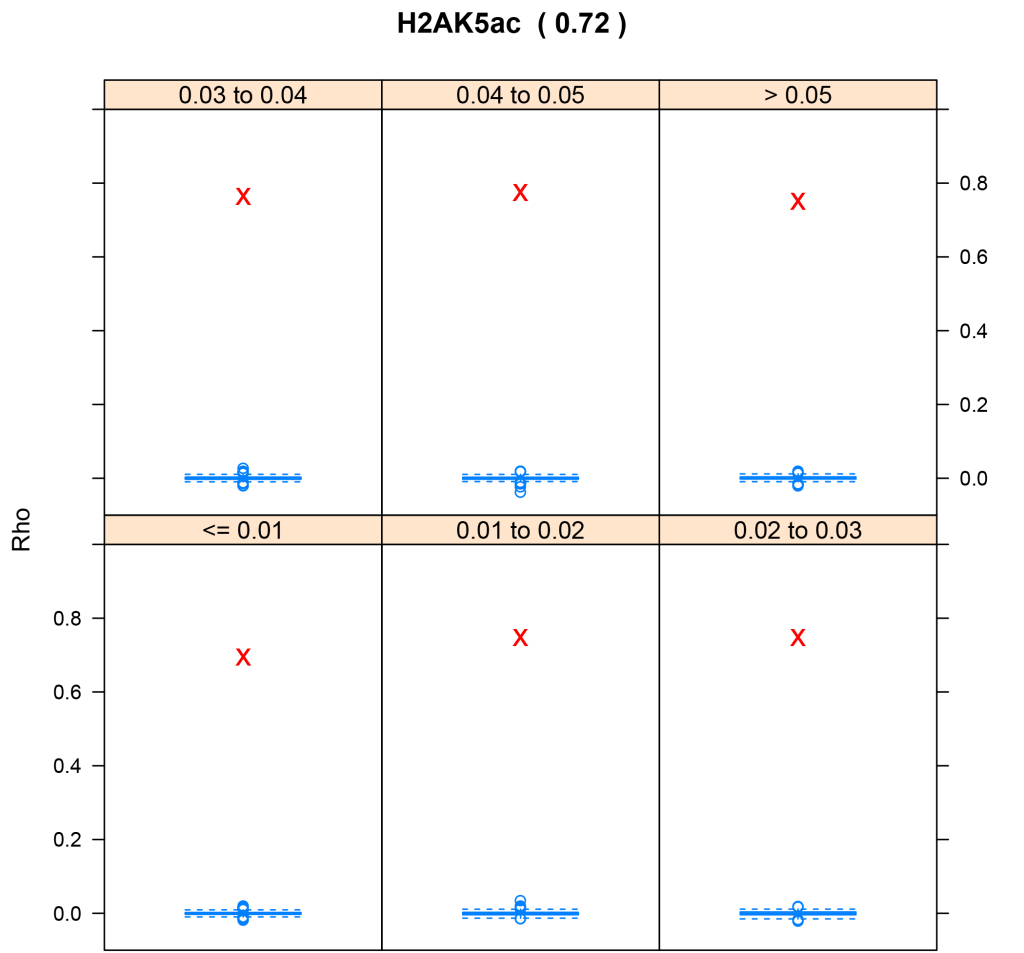


**Figure S4**. Spearmans correlations observed when dividing the paralagous regions into CpG content bins (measured as the average proportion of dincleotides that are CpGs in 1000bp windows at each copy). Each subpanel contains the data for pairs of paralagous regions of a given average CpG dinucleotide content. The correlation observed between the methylation levels/ChIP –seq read counts across the paralagous regions of the given CpG content are indiciated by a red cross. Pairs of sites within each bin were then shuffled with respect to one another 100 times and the correlations resulting from these permutations are shown as a blue box and whisker plot. The corresponding Spearmans correlation coefficient observed across all paralagous pairs is shown in the title.

|  | Enriched regions | Depleted regions | Enriched regions | Depleted regions | Enriched regions | Depleted regions | Enriched regions | Depleted regions | Enriched regions | Depleted regions | Enriched regions | Depleted regions | Enriched regions | Depleted regions | Enriched regions | Depleted regions | Enriched regions | Depleted regions | Enriched regions | Depleted regions | Enriched regions | Depleted regions |  |
| --- | --- | --- | --- | --- | --- | --- | --- | --- | --- | --- | --- | --- | --- | --- | --- | --- | --- | --- | --- | --- | --- | --- | --- |
|  | H3K36me3 | H3K36me3 | H3K9me3 | H3K9me3 | H3K4me2 | H3K4me2 | H3K4me3 | H3K4me3 | H3K4me1 | H3K4me1 | DNaseI | DNaseI | CTCF | CTCF | H3K27me3 | H3K27me3 | H3K9ac | H3K9ac | H3K79me2 | H3K79me2 | H4K20me1 | H4K20me1 |  |
| ATF3(bZIP)/K562-ATF3-ChIP-Seq | 0 | 0 | 0 | 0 | 0 | 0 | 0 | 0 | 0 | 0 | 0 | 0 | 0 | 1 | 0 | 0 | 0 | 0 | 0 | 0 | 0 | 0 | 1 |
| BORIS(Zf)/K562-CTCFL-ChIP-Seq | 1 | 0 | 0 | 0 | 0 | 0 | 0 | 0 | 0 | 0 | 0 | 0 | 0 | 0 | 0 | 0 | 0 | 0 | 0 | 0 | 0 | 0 | 1 |
| Bcl6(Zf)/Liver-Bcl6-ChIP-Seq(GSE31578) | 0 | 0 | 0 | 0 | 0 | 0 | 0 | 0 | 0 | 0 | 0 | 0 | 1 | 0 | 0 | 0 | 0 | 0 | 0 | 0 | 0 | 0 | 1 |
| CArG(MADS)/PUER-Srf-ChIP-Seq | 0 | 0 | 0 | 0 | 1 | 0 | 0 | 0 | 0 | 0 | 0 | 0 | 0 | 0 | 0 | 0 | 0 | 0 | 0 | 0 | 0 | 0 | 1 |
| CRE(bZIP)/Promoter | 1 | 0 | 0 | 0 | 0 | 0 | 0 | 0 | 0 | 0 | 0 | 0 | 0 | 0 | 0 | 0 | 0 | 0 | 0 | 0 | 0 | 0 | 1 |
| CTCF(Zf)/CD4+-CTCF-ChIP-Seq | 0 | 0 | 0 | 0 | 0 | 0 | 0 | 0 | 0 | 0 | 1 | 0 | 1 | 0 | 0 | 0 | 0 | 0 | 0 | 0 | 0 | 0 | 2 |
| Cdx2(Homeobox)/mES-Cdx2-ChIP-Seq | 0 | 0 | 0 | 0 | 0 | 0 | 0 | 0 | 0 | 0 | 1 | 0 | 0 | 0 | 0 | 0 | 1 | 0 | 0 | 0 | 0 | 0 | 2 |
| E2F1(E2F)/Hela-E2F1-ChIP-Seq/Hoemr | 0 | 0 | 0 | 0 | 0 | 0 | 0 | 0 | 0 | 0 | 0 | 0 | 1 | 0 | 0 | 0 | 0 | 0 | 0 | 0 | 0 | 0 | 1 |
| E2F4(E2F)/K562-E2F4-ChIP-Seq(GSE31477) | 0 | 0 | 0 | 0 | 0 | 0 | 0 | 0 | 0 | 0 | 0 | 0 | 1 | 0 | 0 | 0 | 1 | 0 | 0 | 0 | 0 | 0 | 2 |
| E2F6(E2F)/Hela-E2F6-ChIP-Seq(GSE31477) | 0 | 0 | 0 | 0 | 0 | 0 | 0 | 0 | 0 | 0 | 0 | 0 | 1 | 0 | 0 | 0 | 0 | 0 | 0 | 0 | 0 | 0 | 1 |
| EBF(EBF)/proBcell-EBF-ChIP-Seq | 0 | 0 | 0 | 1 | 0 | 0 | 0 | 0 | 0 | 0 | 0 | 0 | 0 | 0 | 0 | 0 | 0 | 0 | 0 | 0 | 0 | 0 | 1 |
| ETS1(ETS)/Jurkat-ETS1-ChIP-Seq | 0 | 0 | 0 | 0 | 0 | 0 | 0 | 0 | 0 | 0 | 0 | 0 | 0 | 0 | 0 | 0 | 1 | 0 | 0 | 0 | 0 | 0 | 1 |
| EWS:FLI1-fusion(ETS)/SK_N_MC-EWS:FLI1-ChIP-Seq | 0 | 0 | 0 | 0 | 0 | 0 | 0 | 0 | 0 | 0 | 0 | 0 | 1 | 0 | 0 | 0 | 1 | 0 | 0 | 0 | 0 | 0 | 2 |
| Elk4(ETS)/Hela-Elk4-ChIP-Seq(GSE31477) | 1 | 0 | 0 | 0 | 0 | 0 | 0 | 0 | 0 | 0 | 0 | 0 | 0 | 0 | 0 | 0 | 0 | 0 | 0 | 0 | 0 | 0 | 1 |
| Esrrb(NR)/mES-Esrrb-ChIP-Seq | 0 | 0 | 0 | 0 | 0 | 0 | 0 | 0 | 0 | 0 | 0 | 0 | 0 | 0 | 0 | 0 | 0 | 0 | 1 | 0 | 0 | 0 | 1 |
| FOXP1(Forkhead)/H9-FOXP1-ChIP-Seq(GSE31006) | 0 | 0 | 0 | 0 | 1 | 0 | 0 | 0 | 0 | 0 | 0 | 0 | 0 | 0 | 0 | 0 | 0 | 0 | 0 | 0 | 0 | 0 | 1 |
| FXR(NR/IR1)/Liver-FXR-ChIP-Seq | 0 | 0 | 1 | 0 | 0 | 0 | 0 | 0 | 0 | 0 | 0 | 0 | 0 | 0 | 1 | 0 | 0 | 0 | 0 | 0 | 0 | 0 | 2 |
| Fli1(ETS)/CD8-FLI-ChIP-Seq(GSE20898) | 0 | 0 | 0 | 0 | 0 | 0 | 0 | 0 | 0 | 0 | 0 | 0 | 0 | 0 | 0 | 0 | 1 | 0 | 0 | 0 | 0 | 0 | 1 |
| Foxh1(Forkhead)/hESC-FOXH1-ChIP-Seq(GSE29422) | 0 | 0 | 0 | 0 | 0 | 0 | 1 | 0 | 0 | 0 | 0 | 0 | 0 | 0 | 0 | 0 | 1 | 0 | 0 | 0 | 0 | 0 | 2 |
| Foxo1(Forkhead)/RAW-Foxo1-ChIP-Seq | 0 | 0 | 0 | 0 | 1 | 0 | 0 | 0 | 0 | 0 | 0 | 0 | 0 | 0 | 0 | 0 | 0 | 0 | 0 | 0 | 0 | 0 | 1 |
| GATA-DR4(Zf)/iTreg-Gata3-ChIP-Seq(GSE20898) | 0 | 0 | 0 | 0 | 0 | 0 | 0 | 0 | 0 | 0 | 1 | 0 | 0 | 0 | 0 | 0 | 0 | 0 | 0 | 0 | 0 | 0 | 1 |
| GATA-DR8(Zf)/iTreg-Gata3-ChIP-Seq(GSE20898) | 1 | 0 | 0 | 0 | 0 | 0 | 0 | 0 | 0 | 0 | 0 | 0 | 0 | 0 | 0 | 0 | 0 | 0 | 0 | 0 | 0 | 0 | 1 |
| GATA-IR3(Zf)/iTreg-Gata3-ChIP-Seq(GSE20898) | 0 | 0 | 0 | 0 | 0 | 0 | 0 | 0 | 0 | 0 | 0 | 0 | 0 | 1 | 0 | 0 | 0 | 0 | 0 | 0 | 0 | 0 | 1 |
| GATA3(Zf)/iTreg-Gata3-ChIP-Seq(GSE20898) | 0 | 1 | 0 | 0 | 0 | 0 | 0 | 0 | 0 | 0 | 0 | 0 | 0 | 0 | 0 | 0 | 0 | 0 | 0 | 0 | 0 | 0 | 1 |
| GRE/RAW264.7-GRE-ChIP-Seq | 0 | 0 | 0 | 0 | 0 | 0 | 0 | 0 | 0 | 0 | 0 | 0 | 1 | 0 | 0 | 0 | 0 | 0 | 0 | 0 | 0 | 0 | 1 |
| Gata1(Zf)/K562-GATA1-ChIP-Seq | 0 | 1 | 0 | 0 | 0 | 0 | 0 | 0 | 0 | 0 | 0 | 0 | 0 | 0 | 0 | 0 | 0 | 0 | 0 | 0 | 0 | 0 | 1 |
| Gata2(Zf)/K562-GATA2-ChIP-Seq | 0 | 1 | 0 | 0 | 0 | 0 | 0 | 0 | 0 | 0 | 0 | 0 | 0 | 0 | 0 | 0 | 0 | 0 | 0 | 0 | 0 | 0 | 1 |
| HIF-1a(HLH)/MCF7-HIF1a-ChIP-Seq | 0 | 0 | 0 | 0 | 0 | 0 | 0 | 0 | 0 | 0 | 0 | 0 | 0 | 1 | 0 | 0 | 0 | 0 | 0 | 0 | 0 | 0 | 1 |
| HIF2a(HLH)/O785-HIF2a-ChIP-Seq(GSE34871) | 0 | 0 | 0 | 0 | 0 | 0 | 0 | 0 | 0 | 0 | 0 | 0 | 1 | 0 | 0 | 0 | 0 | 0 | 0 | 0 | 0 | 0 | 1 |
| HNF4a(NR/DR1)/HepG2-HNF4a-ChIP-Seq | 0 | 0 | 0 | 0 | 0 | 0 | 0 | 0 | 0 | 0 | 0 | 0 | 0 | 0 | 1 | 0 | 1 | 0 | 0 | 0 | 0 | 0 | 2 |
| HRE(HSF)/HepG2-HSF1-ChIP-Seq | 0 | 0 | 0 | 0 | 0 | 0 | 0 | 0 | 0 | 0 | 0 | 0 | 1 | 0 | 0 | 0 | 0 | 0 | 0 | 0 | 0 | 0 | 1 |
| Hnf1(Homeobox)/Liver-Foxa2-Chip-Seq | 0 | 0 | 0 | 0 | 0 | 0 | 0 | 0 | 0 | 0 | 1 | 0 | 0 | 0 | 0 | 0 | 0 | 0 | 0 | 0 | 0 | 0 | 1 |
| Lhx3(Homeobox)/Forebrain-p300-ChIP-Seq | 0 | 0 | 0 | 0 | 0 | 0 | 0 | 0 | 0 | 0 | 0 | 0 | 0 | 0 | 1 | 0 | 0 | 0 | 0 | 0 | 0 | 0 | 1 |
| Max(HLH)/K562-Max-ChIP-Seq | 0 | 0 | 0 | 0 | 0 | 0 | 0 | 0 | 0 | 0 | 0 | 0 | 0 | 0 | 0 | 0 | 1 | 0 | 0 | 0 | 0 | 0 | 1 |
| Mef2c(MADS)/GM12878-Mef2c-ChIP-Seq(GSE32465) | 0 | 0 | 1 | 0 | 0 | 0 | 0 | 0 | 0 | 0 | 0 | 0 | 1 | 0 | 0 | 0 | 0 | 0 | 0 | 0 | 0 | 0 | 2 |
| NF1(CTF)/LNCAP-NF1-ChIP-Seq | 0 | 0 | 0 | 0 | 0 | 0 | 0 | 0 | 0 | 0 | 0 | 0 | 0 | 0 | 1 | 0 | 0 | 0 | 0 | 0 | 0 | 0 | 1 |
| NFAT(RHD)/Jurkat-NFATC1-ChIP-Seq | 0 | 0 | 0 | 0 | 0 | 0 | 0 | 0 | 0 | 0 | 0 | 0 | 1 | 0 | 0 | 0 | 0 | 0 | 0 | 0 | 0 | 0 | 1 |
| NFAT:AP1/Jurkat-NFATC1-ChIP-Seq | 0 | 0 | 0 | 0 | 0 | 0 | 0 | 0 | 0 | 0 | 0 | 1 | 0 | 0 | 0 | 0 | 0 | 0 | 0 | 0 | 0 | 0 | 1 |
| NFkB-p65-Rel(RHD)/LPS-exp | 0 | 0 | 0 | 0 | 1 | 0 | 0 | 0 | 0 | 0 | 0 | 0 | 0 | 0 | 0 | 0 | 0 | 0 | 0 | 0 | 0 | 0 | 1 |
| NRF1/Promoter | 0 | 0 | 1 | 0 | 0 | 0 | 0 | 0 | 0 | 0 | 0 | 0 | 0 | 0 | 0 | 0 | 0 | 0 | 0 | 0 | 0 | 0 | 1 |
| Nr5a2(NR)/mES-Nr5a2-ChIP-Seq | 0 | 0 | 1 | 0 | 0 | 0 | 0 | 0 | 0 | 0 | 1 | 0 | 0 | 0 | 0 | 0 | 0 | 0 | 0 | 0 | 0 | 0 | 2 |
| Oct2(POU/Homeobox)/Bcell-Oct2-ChIP-Seq | 0 | 0 | 0 | 0 | 1 | 0 | 0 | 0 | 0 | 0 | 0 | 0 | 0 | 0 | 0 | 0 | 0 | 0 | 0 | 0 | 0 | 0 | 1 |
| PAX3:FKHR-fusion(Paired/Homeobox)/Rh4-PAX3:FKHR-ChIP-Seq | 0 | 0 | 0 | 1 | 0 | 0 | 0 | 0 | 0 | 0 | 0 | 0 | 0 | 0 | 0 | 0 | 0 | 0 | 0 | 0 | 0 | 0 | 1 |
| Pax7-longest(Paired/Homeobox)/Myoblast-Pax7-ChIP-Seq(GSE25064) | 0 | 0 | 1 | 0 | 0 | 0 | 0 | 0 | 0 | 0 | 0 | 0 | 0 | 0 | 0 | 0 | 0 | 0 | 0 | 0 | 0 | 0 | 1 |
| Reverb(NR/DR2)/BLRP(RAW)-Reverba-ChIP-Seq | 0 | 0 | 1 | 0 | 0 | 0 | 0 | 0 | 0 | 0 | 0 | 0 | 0 | 0 | 0 | 0 | 0 | 0 | 0 | 0 | 0 | 0 | 1 |
| Rfx1(HTH)/NPC-Rfx1-ChIP-Seq | 0 | 0 | 0 | 0 | 0 | 0 | 0 | 0 | 1 | 0 | 1 | 0 | 0 | 0 | 0 | 0 | 0 | 0 | 0 | 0 | 0 | 0 | 2 |
| Rfx5(HTH)/GM12878-Rfx5-ChIP-Seq(GSE31477) | 0 | 0 | 0 | 1 | 0 | 0 | 0 | 0 | 0 | 0 | 0 | 0 | 0 | 0 | 0 | 0 | 0 | 0 | 0 | 0 | 0 | 0 | 1 |
| SPDEF(ETS)/VCaP-SPDEF-ChIP-Seq | 0 | 0 | 0 | 0 | 0 | 0 | 0 | 0 | 0 | 0 | 0 | 0 | 0 | 0 | 1 | 0 | 0 | 0 | 0 | 0 | 0 | 0 | 1 |
| STAT6/Macrophage-Stat6-ChIP-Seq | 0 | 0 | 0 | 0 | 0 | 0 | 0 | 0 | 0 | 0 | 0 | 0 | 1 | 0 | 0 | 0 | 0 | 0 | 0 | 0 | 0 | 0 | 1 |
| Sox3(HMG)/NPC-Sox3-ChIP-Seq(GSE33059) | 0 | 0 | 0 | 0 | 0 | 0 | 0 | 0 | 0 | 0 | 0 | 0 | 0 | 0 | 0 | 0 | 1 | 0 | 0 | 0 | 0 | 0 | 1 |
| Sox6(HMG)/Myotubes-Sox6-ChIP-Seq(GSE32627) | 0 | 0 | 0 | 0 | 0 | 0 | 1 | 0 | 0 | 0 | 0 | 0 | 0 | 0 | 0 | 0 | 0 | 0 | 1 | 0 | 0 | 0 | 2 |
| Srebp1a(HLH)/HepG2-Srebp1a-ChIP-Seq | 0 | 0 | 1 | 0 | 0 | 0 | 0 | 0 | 0 | 0 | 0 | 0 | 0 | 0 | 0 | 0 | 0 | 0 | 0 | 0 | 0 | 0 | 1 |
| TATA-Box(TBP)/Promoter | 0 | 0 | 0 | 0 | 0 | 0 | 0 | 0 | 0 | 0 | 0 | 0 | 0 | 0 | 0 | 0 | 0 | 0 | 0 | 0 | 1 | 0 | 1 |
| TEAD(TEA)/Fibroblast-PU.1-ChIP-Seq | 0 | 0 | 0 | 0 | 1 | 0 | 0 | 0 | 0 | 0 | 0 | 0 | 0 | 0 | 0 | 0 | 0 | 0 | 0 | 0 | 0 | 0 | 1 |
| TEAD4(TEA)/Tropoblast-Tead4-ChIP-Seq(GSE37350) | 0 | 0 | 0 | 0 | 1 | 0 | 0 | 0 | 0 | 0 | 0 | 0 | 0 | 0 | 0 | 0 | 0 | 0 | 0 | 0 | 0 | 0 | 1 |
| Tbet(T-box)/CD8-Tbet-ChIP-Seq(GSE33802) | 0 | 0 | 0 | 0 | 0 | 0 | 0 | 0 | 0 | 0 | 0 | 0 | 0 | 0 | 1 | 0 | 0 | 0 | 0 | 0 | 0 | 0 | 1 |
| Tbox:Smad/ESCd5-Smad2_3-ChIP-Seq(GSE29422) | 0 | 0 | 0 | 0 | 0 | 0 | 1 | 0 | 0 | 0 | 0 | 0 | 0 | 0 | 0 | 0 | 0 | 0 | 0 | 0 | 0 | 0 | 1 |
| Tbx5(T-box)/HL1-Tbx5.biotin-ChIP-Seq | 0 | 0 | 0 | 0 | 0 | 0 | 0 | 0 | 0 | 0 | 0 | 0 | 0 | 0 | 0 | 0 | 0 | 0 | 0 | 0 | 1 | 0 | 1 |
| Tcfcp2l1(CP2)/mES-Tcfcp2l1-ChIP-Seq | 0 | 0 | 0 | 0 | 1 | 0 | 0 | 0 | 0 | 0 | 0 | 0 | 0 | 0 | 0 | 0 | 0 | 0 | 0 | 0 | 0 | 0 | 1 |
| USF1(HLH)/GM12878-Usf1-ChIP-Seq | 0 | 0 | 0 | 0 | 0 | 0 | 0 | 0 | 0 | 0 | 0 | 0 | 0 | 1 | 0 | 0 | 0 | 0 | 0 | 0 | 0 | 0 | 1 |
| Usf2(HLH)/C2C12-Usf2-ChIP-Seq(GSE36030) | 0 | 0 | 0 | 0 | 0 | 0 | 0 | 0 | 0 | 0 | 0 | 0 | 0 | 1 | 0 | 0 | 0 | 0 | 0 | 0 | 0 | 0 | 1 |
| VDR(NR/DR3)/GM10855-VDR+vitD-ChIP-Seq | 0 | 0 | 0 | 0 | 0 | 0 | 0 | 0 | 0 | 0 | 0 | 0 | 1 | 0 | 0 | 0 | 0 | 0 | 0 | 0 | 0 | 0 | 1 |
| X-box(HTH)/NPC-H3K4me1-ChIP-Seq | 0 | 0 | 0 | 0 | 0 | 0 | 0 | 0 | 1 | 0 | 0 | 0 | 0 | 0 | 0 | 0 | 0 | 0 | 0 | 0 | 0 | 0 | 1 |
| bHLHE40(HLH)/HepG2-BHLHE40-ChIP-Seq | 0 | 0 | 1 | 0 | 0 | 0 | 0 | 0 | 0 | 0 | 0 | 0 | 0 | 0 | 0 | 0 | 0 | 0 | 0 | 0 | 0 | 0 | 1 |
| c-Jun-CRE(bZIP)/K562-cJun-ChIP-Seq | 0 | 0 | 0 | 0 | 0 | 0 | 0 | 0 | 0 | 0 | 0 | 0 | 1 | 0 | 0 | 0 | 0 | 0 | 0 | 0 | 0 | 0 | 1 |
| c-Myc(HLH)/LNCAP-cMyc-ChIP-Seq | 0 | 0 | 1 | 0 | 0 | 0 | 0 | 0 | 0 | 0 | 0 | 0 | 0 | 1 | 0 | 0 | 0 | 0 | 0 | 0 | 0 | 0 | 2 |
| n-Myc(HLH)/mES-nMyc-ChIP-Seq | 0 | 0 | 1 | 0 | 0 | 0 | 0 | 0 | 0 | 0 | 0 | 0 | 0 | 0 | 0 | 0 | 0 | 0 | 0 | 0 | 0 | 0 | 1 |
| p53(p53)/Saos-p53-ChIP-Seq | 1 | 0 | 0 | 0 | 0 | 0 | 0 | 0 | 0 | 0 | 0 | 0 | 0 | 0 | 0 | 0 | 0 | 0 | 0 | 0 | 0 | 0 | 1 |
| p63(p53)/Keratinocyte-p63-ChIP-Seq | 1 | 0 | 0 | 0 | 0 | 0 | 0 | 0 | 0 | 0 | 0 | 0 | 1 | 0 | 0 | 0 | 0 | 0 | 0 | 0 | 0 | 0 | 2 |
|  | 6 | 3 | 10 | 3 | 8 | 0 | 3 | 0 | 2 | 0 | 6 | 1 | 15 | 6 | 6 | 0 | 9 | 0 | 2 | 0 | 2 | 0 |  |

**Table S1**: Known DNA binding motifs found to be significantly enriched (HOMER Benjamini q value < 0.01) at the chromatin enriched or depleted copies of discordant paralogous regions in the H1 cell line are indicated with a 1. Row and column counts are shown.

| **Unmethylated copy** |  | **Methylated copy** |  |
| --- | --- | --- | --- |
| **Chr** | **Pos** | **Chr** | **Pos** |
| chr1 | 17059399 | chr1 | 16924767 |
| chr1 | 46956776 | chr1 | 35359807 |
| chr1 | 141813287 | chr3 | 75901633 |
| chr10 | 19818142 | chr17 | 38550605 |
| chr10 | 135367457 | chr22 | 49585848 |
| chr11 | 2923301 | chr1 | 116876964 |
| chr14 | 18176718 | chr2 | 132639212 |
| chr16 | 5125404 | chr4 | 9248544 |
| chr17 | 16224968 | chr17 | 21653874 |
| chr17 | 16225769 | chr17 | 21654648 |
| chr17 | 16590391 | chr17 | 20220332 |
| chr17 | 16590391 | chr9 | 101800884 |
| chr17 | 16590412 | chr9 | 101800905 |
| chr17 | 21308304 | chr17 | 21929594 |
| chr17 | 21308316 | chr17 | 21929582 |
| chr17 | 30845152 | chr6 | 20601560 |
| chr17 | 38577179 | chr17 | 38534184 |
| chr17 | 41016767 | chr17 | 63714956 |
| chr17 | 41016971 | chr17 | 63714751 |
| chr17 | 41016981 | chr17 | 26383707 |
| chr17 | 63253690 | chr17 | 63714750 |
| chr17 | 63253692 | chr17 | 26383718 |
| chr17 | 63714107 | chr17 | 26383074 |
| chr19 | 11860235 | chr19 | 11787725 |
| chr19 | 11860242 | chr19 | 11787732 |
| chr19 | 11860281 | chr19 | 11787771 |
| chr2 | 70169708 | chr1 | 109447327 |
| chr2 | 132823172 | chr19 | 49551027 |
| chr2 | 144863595 | chr4 | 15971516 |
| chr2 | 144863605 | chr4 | 15971526 |
| chr20 | 23597039 | chr20 | 23727824 |
| chr20 | 51644764 | chr21 | 10013225 |
| chr22 | 15431750 | chr3 | 180417524 |
| chr22 | 37125313 | chr21 | 10013225 |
| chr3 | 123862526 | chr10 | 21358315 |
| chr3 | 123862526 | chr16 | 46157927 |
| chr3 | 182914404 | chr8 | 125384643 |
| chr4 | 156604695 | chr2 | 212346835 |
| chr4 | 156604695 | chr17 | 19445549 |
| chr4 | 156604742 | chr20 | 55367153 |
| chr4 | 156604748 | chr20 | 55367159 |
| chr6 | 26809632 | chr6 | 58276582 |
| chr6 | 26809657 | chr6 | 58276557 |
| chr6 | 26809799 | chr6 | 58276415 |
| chr6 | 105507999 | chr5 | 162775124 |
| chr6 | 105508860 | chr5 | 162775992 |
| chr6 | 150351338 | chr6 | 150396616 |
| chr7 | 125711 | chr9 | 140232020 |
| chr7 | 125711 | chr16 | 88701035 |
| chr7 | 138838717 | chr12 | 56664226 |
| chr8 | 12339574 | chr3 | 75568299 |
| chrX | 52980246 | chrX | 52968371 |
| chrX | 52980273 | chrX | 52968345 |

**Table S2**: The 53 pairs of sites with a p<5x10^-7^ (Fisher’s Exact test) for differential methylation between paralogous sites in the H1 cell line.


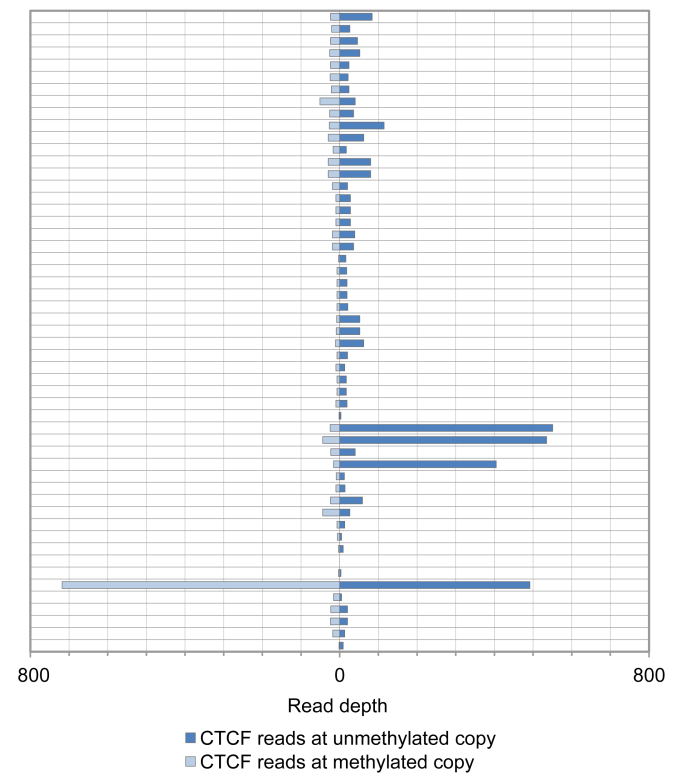

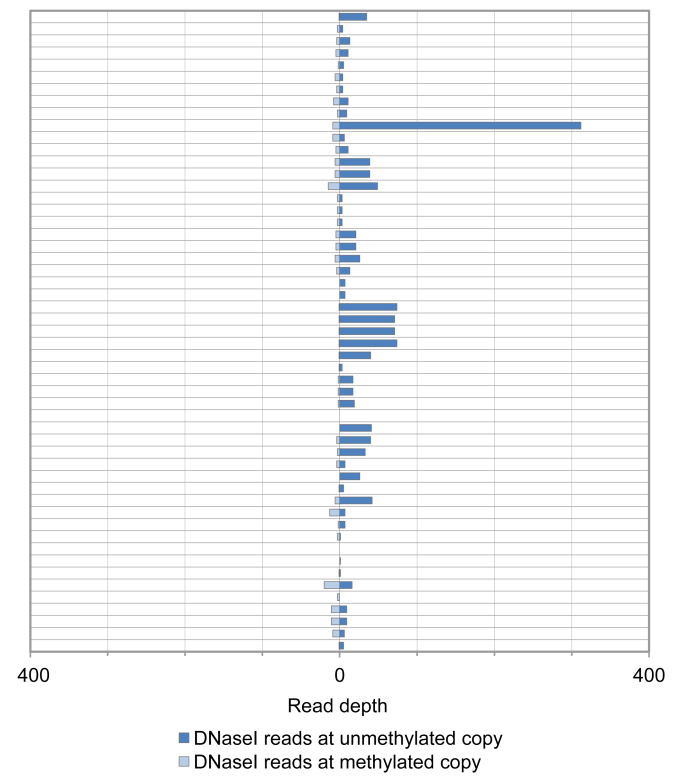

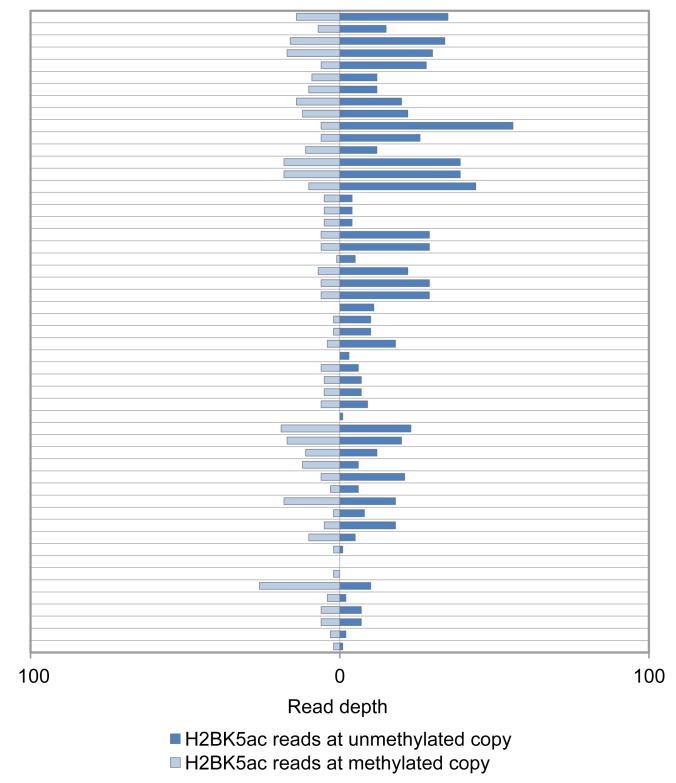

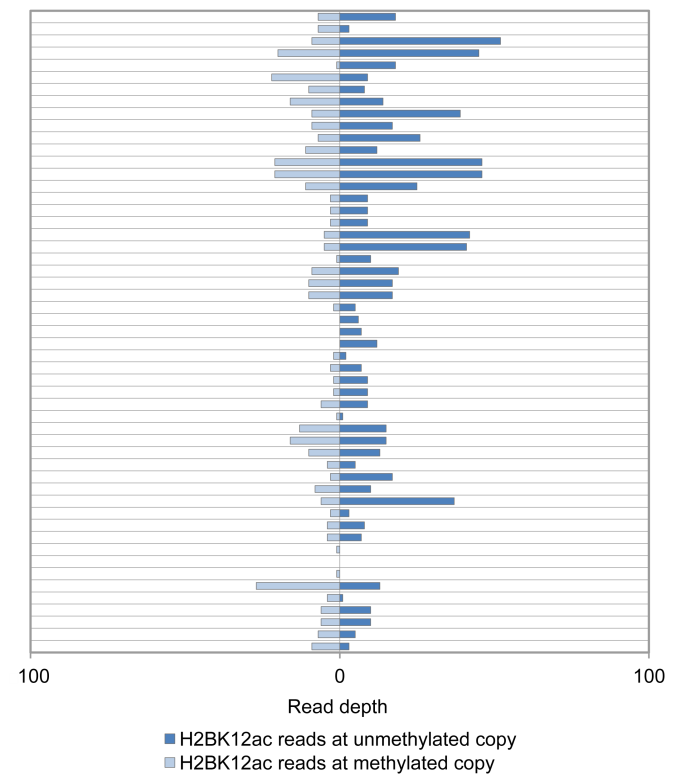

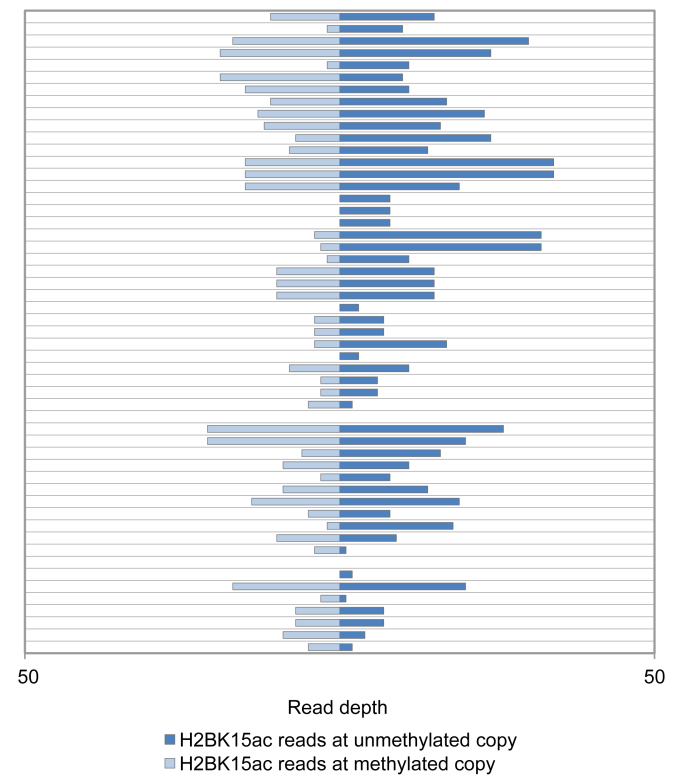

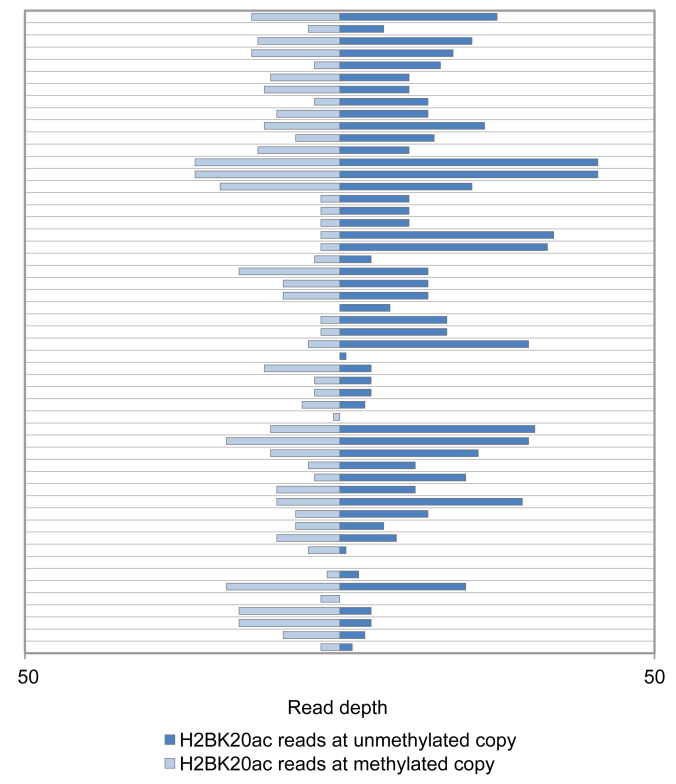

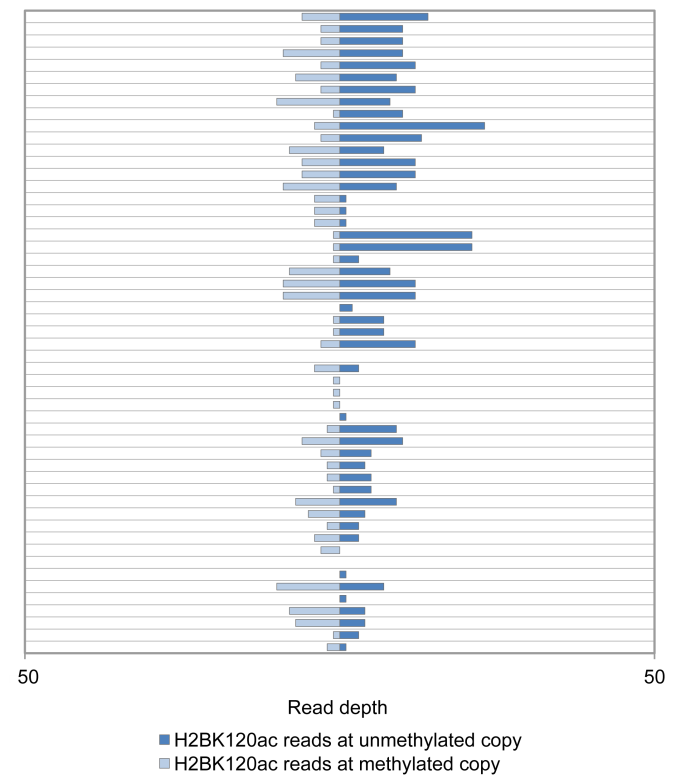

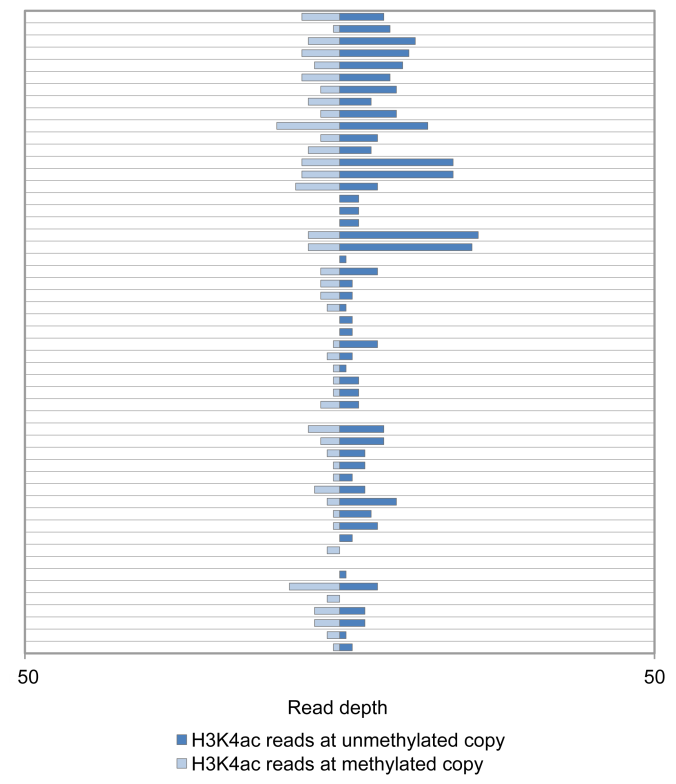

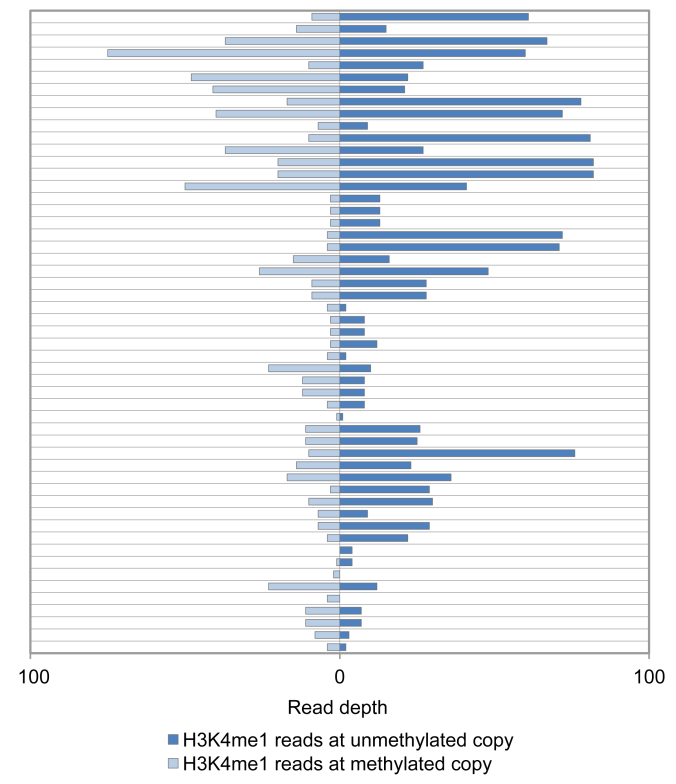

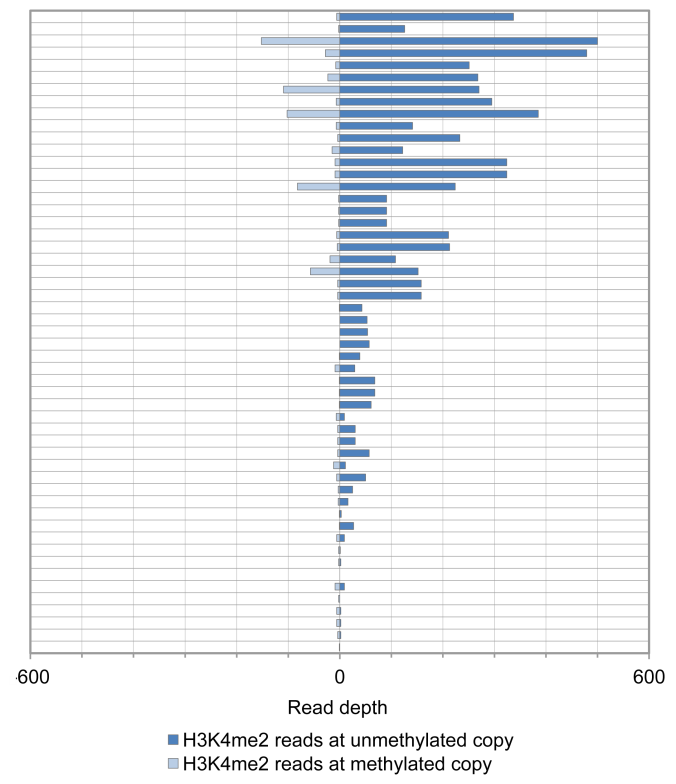

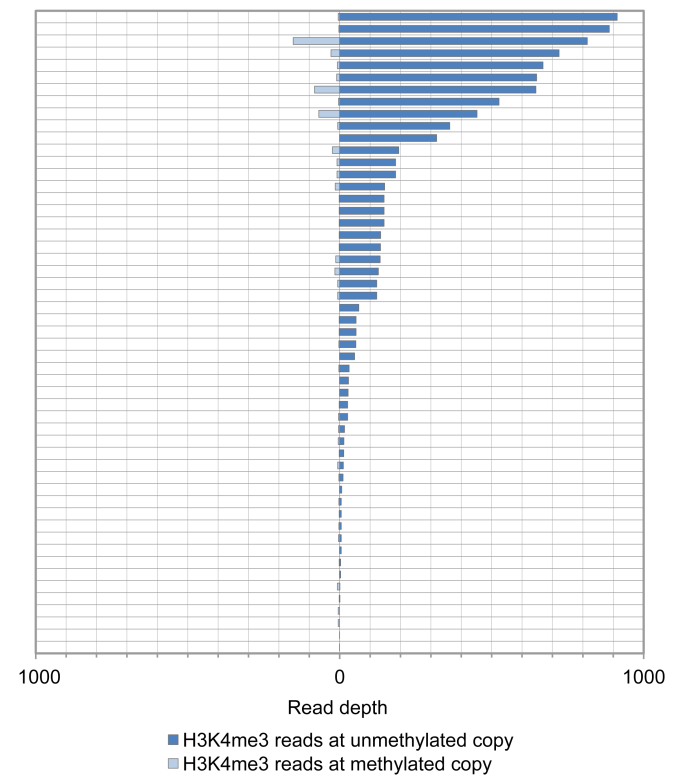

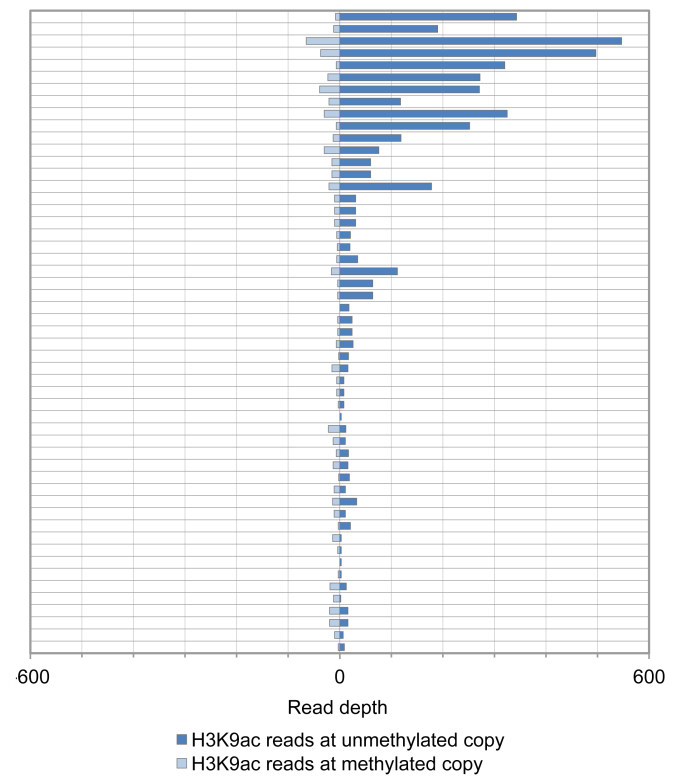

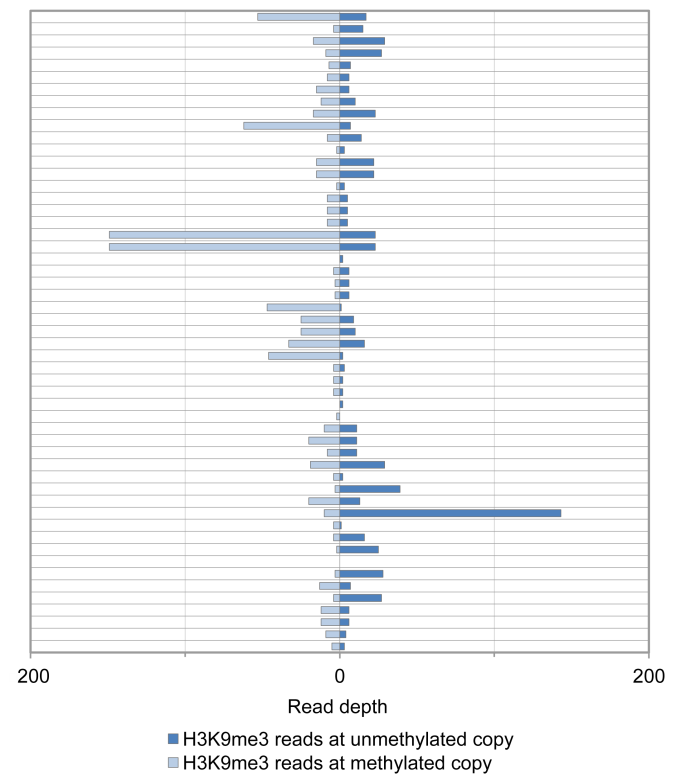

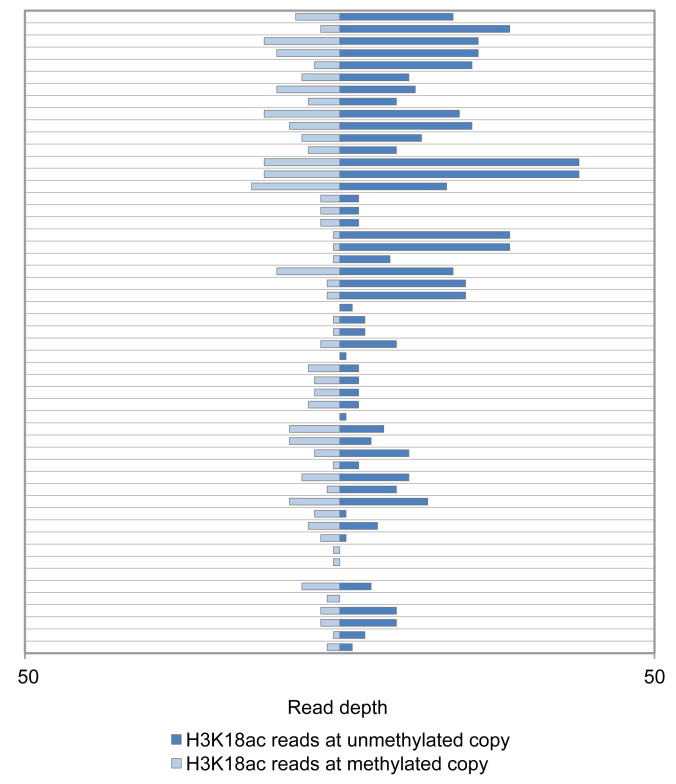

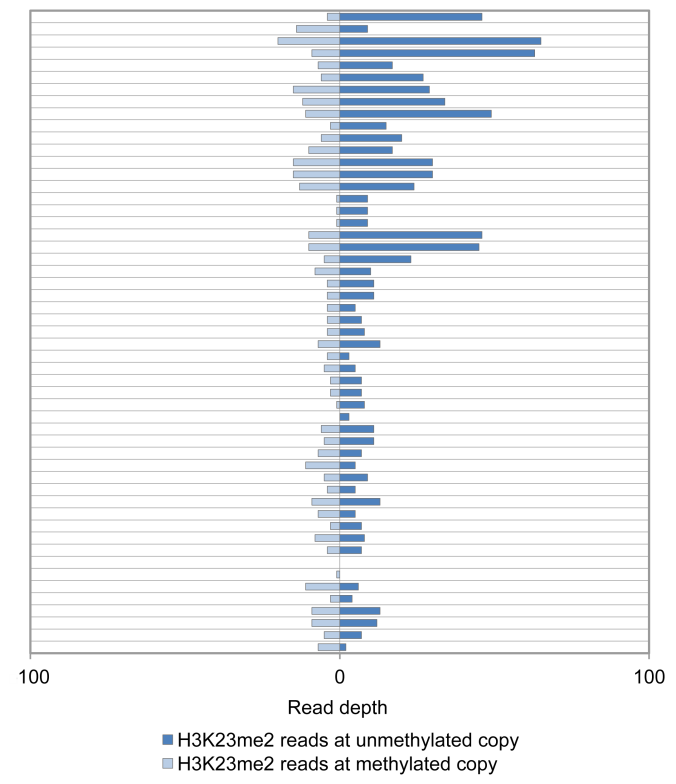

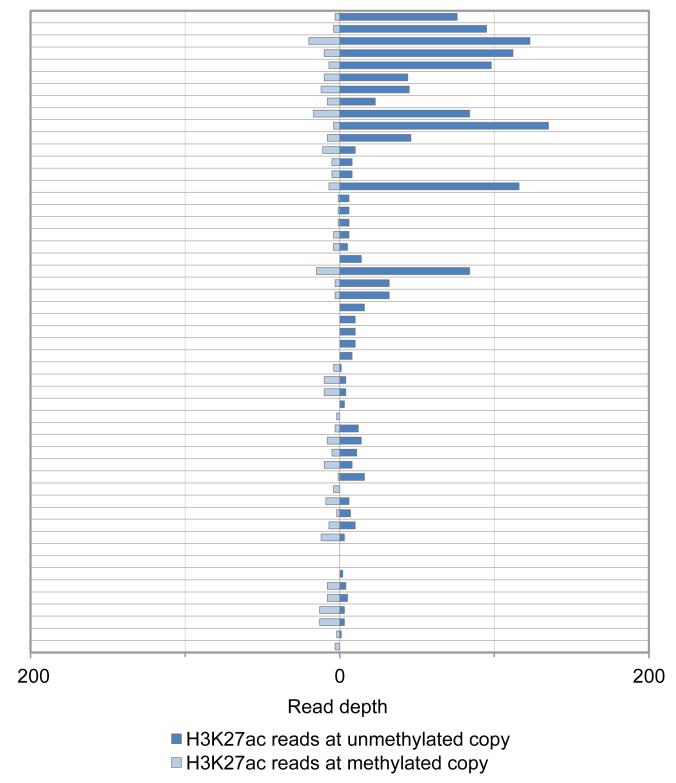

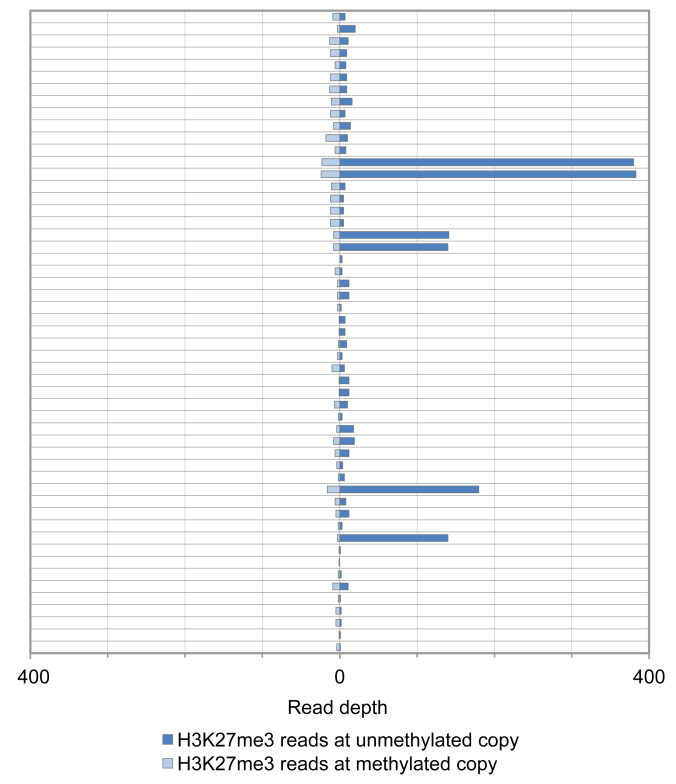

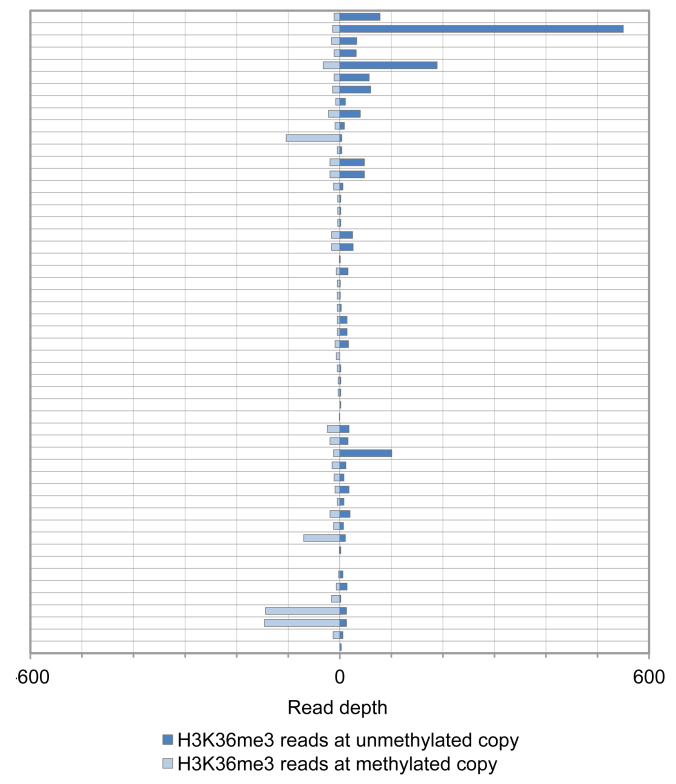

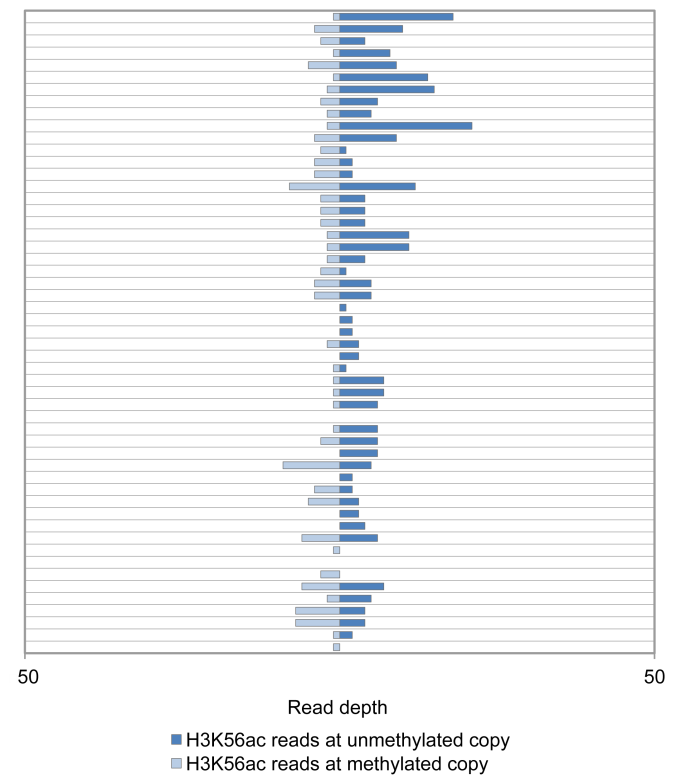

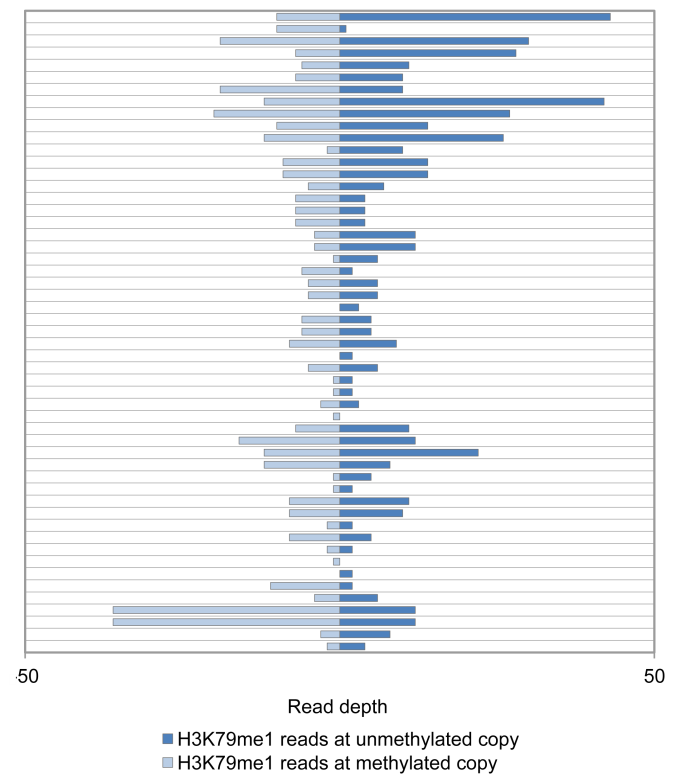

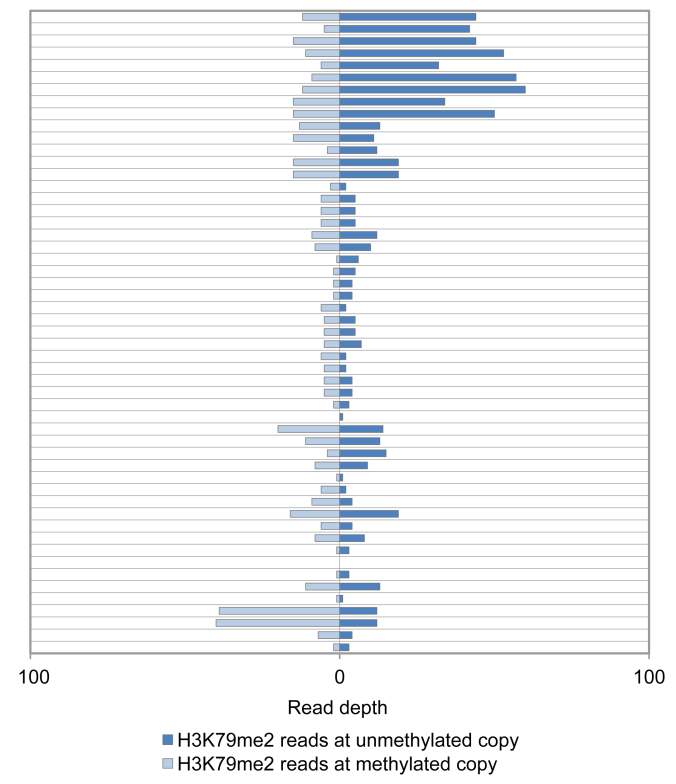

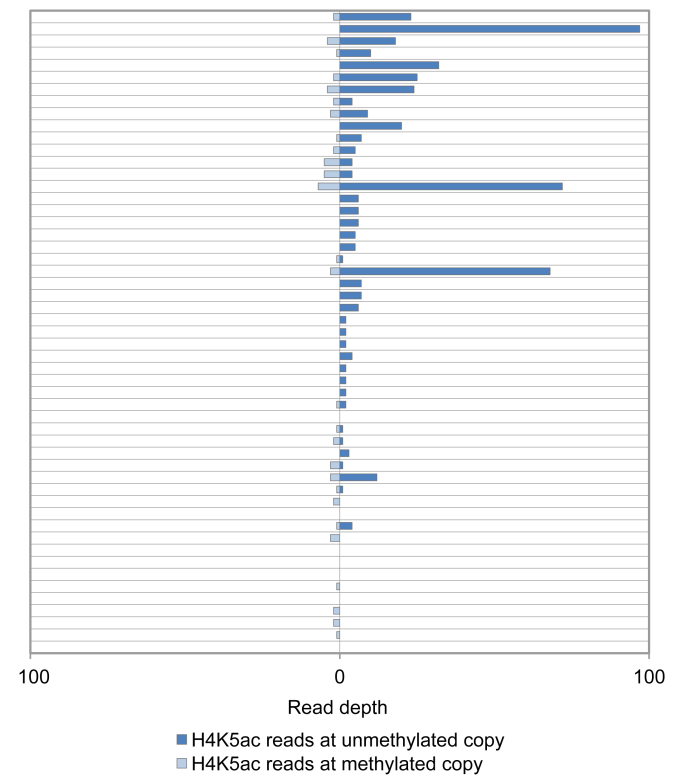

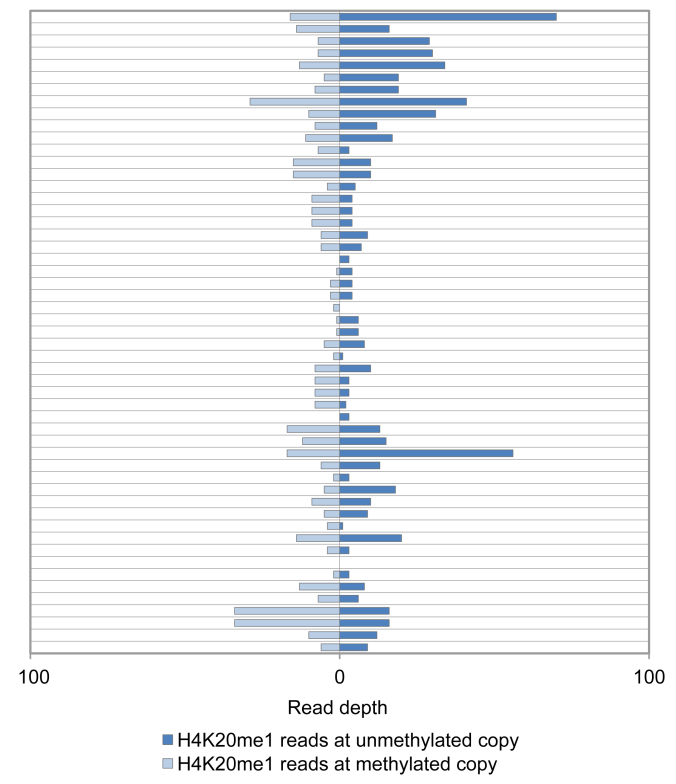

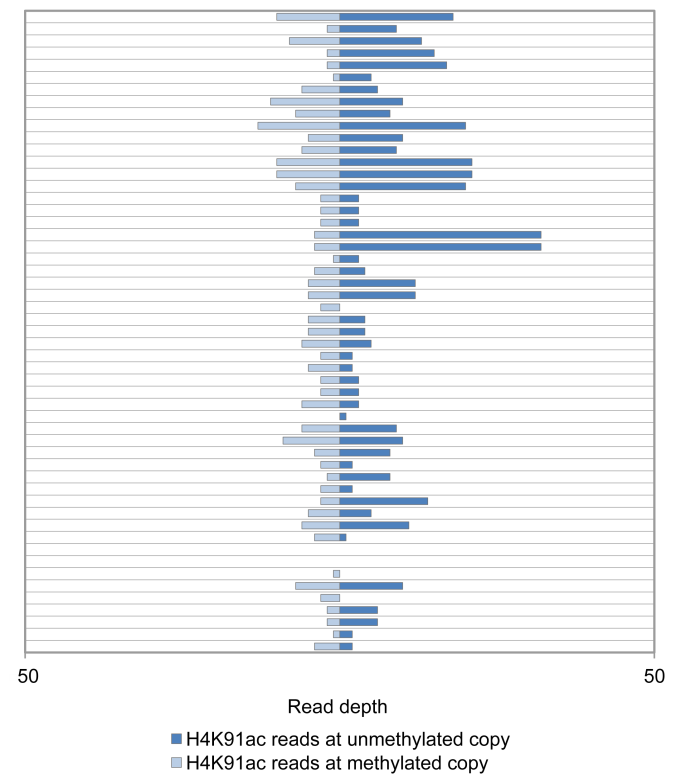


**
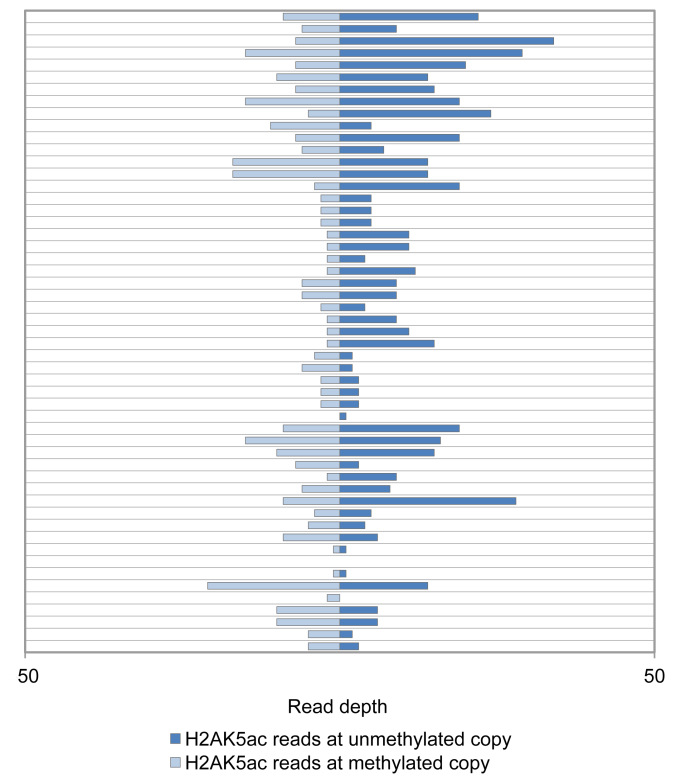
**

**Figure S5**: ChIP-seq/DNase-seq read counts observed at the methylated and unmethylated copies of paralogous CpG sites discordant in their methylation levels. Read counts for 23 histone modifications, CTCF and DNase I are shown. Each row corresponds to one of the 53 pairs of sites displaying significantly different methylation levels between copies (ranked in the same order in each plot according to the H3K4me3 read count observed at the unmethylated copy of the pair of sites).

|  | **Number of pairs of paralogous sites read count was higher at:** | |  |
| --- | --- | --- | --- |
| **Modification** | **Unmethylated copy** | **Methylated copy** | **p value** |
| *H3K4me3* | 47 | 6 | 0.000000006 |
| *SP1* | 44 | 9 | 0.0000012 |
| *H3K4me2* | 43 | 10 | 0.0000056 |
| *H3K23me2* | 42 | 11 | 0.000022 |
| *H3K9ac* | 41 | 12 | 0.000082 |
| *H2BK15ac* | 40 | 13 | 0.00027 |
| *CTCF* | 40 | 13 | 0.00027 |
| *H2BK5ac* | 39 | 14 | 0.00080 |
| *H2BK12ac* | 38 | 15 | 0.0022 |
| *H2BK20ac* | 37 | 16 | 0.0055 |
| *H3K56ac* | 37 | 16 | 0.0055 |
| *DNase I* | 37 | 16 | 0.0055 |
| *H3K18ac* | 36 | 17 | 0.013 |
| *H3K27ac* | 36 | 17 | 0.013 |
| *H3K4ac* | 35 | 18 | 0.027 |
| *H3K4me1* | 35 | 18 | 0.027 |
| *H3K79me1* | 34 | 19 | 0.053 |
| *H4K20me1* | 34 | 19 | 0.053 |
| *H2AK5ac* | 33 | 20 | 0.098 |
| *H2BK120ac* | 33 | 20 | 0.098 |
| *H4K5ac* | 33 | 20 | 0.098 |
| *H3K9me3* | 23 | 30 | 0.41 |
| *H3K27me3* | 29 | 24 | 0.58 |
| *H3K79me2* | 29 | 24 | 0.58 |
| *H4K91ac* | 29 | 24 | 0.58 |
| *H3K36me3* | 26 | 27 | 1 |

**Table S3**: Types of chromatin displaying significant imbalances around the unmethylated and methylated copies of discordant, paralogous CpG sites. Binomial p values are shown.


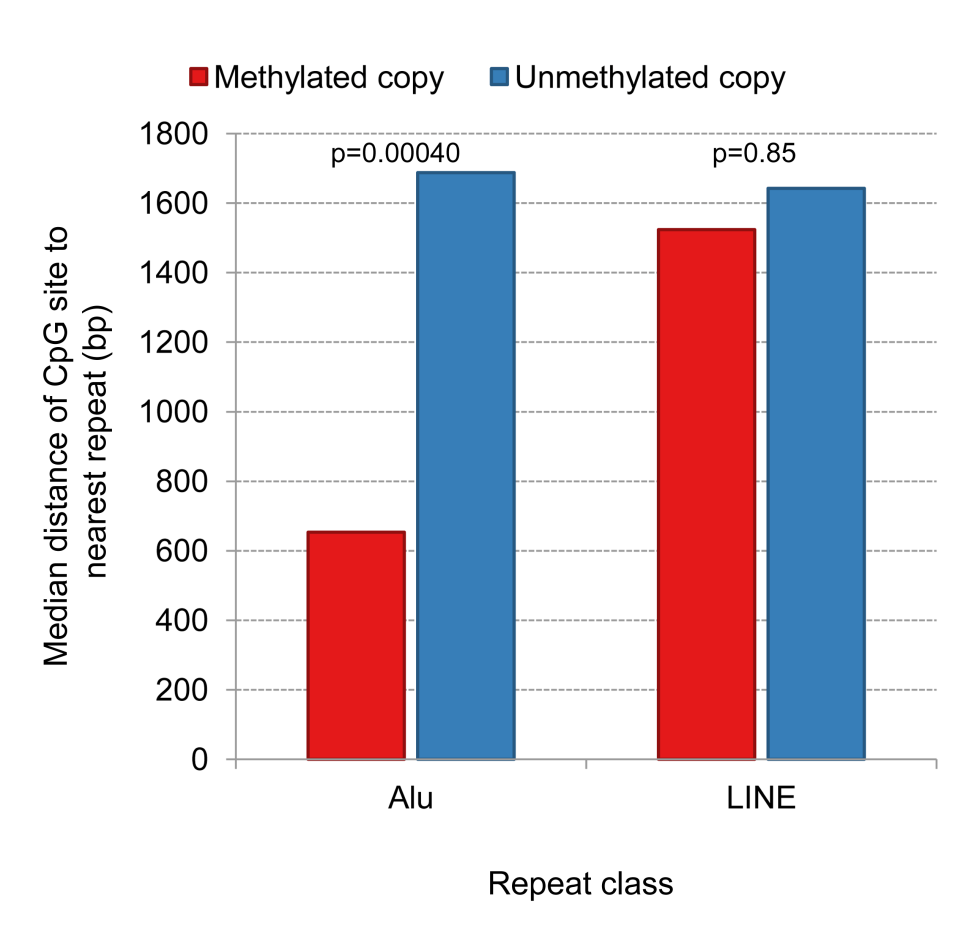


**Figure S7**: Alu elements are preferentially associated with methylated copies of discordant CpG sites in human embryonic stem cells. P values derived using a paired Mann-Whitney test.


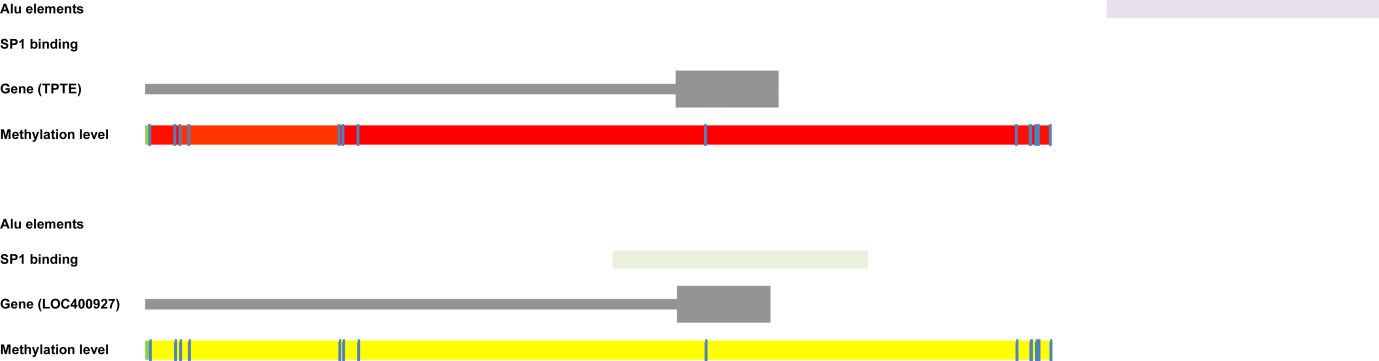


**Figure S8**: Divergence in methylation levels at the paralogous TPTE and LOC400927 loci. Paralagous CpG sites covered in this analysis are shown by vertical blue dashes. Neighbouring CpG sites are connected by a coloured bar corresponding to their lowest methylation level in the H1 cell line. Low methylation levels are shown in yellow, high methylation levels in red. All CpG sites at the LOC400927 had a methylation level of 0% methylated. All CpG sites at the TPTE locus had a methylation level greater than 90%. The location of Alu elements and SP1 binding were obtained from the UCSC genome browser. SP1 binding was confirmed to be 16 times higher at the LOC400927 locus in the analysis of the H1 SP1 ChIP-seq dataset with the expression level of LOC400927 observed to be approximately 30 times higher than TPTE (LOC400927 reads per kilobase per million reads (RPKM): 1.79; TPTE RPKM: 0.061). Exons are indicated by thick grey bars, introns by a thin grey bar.


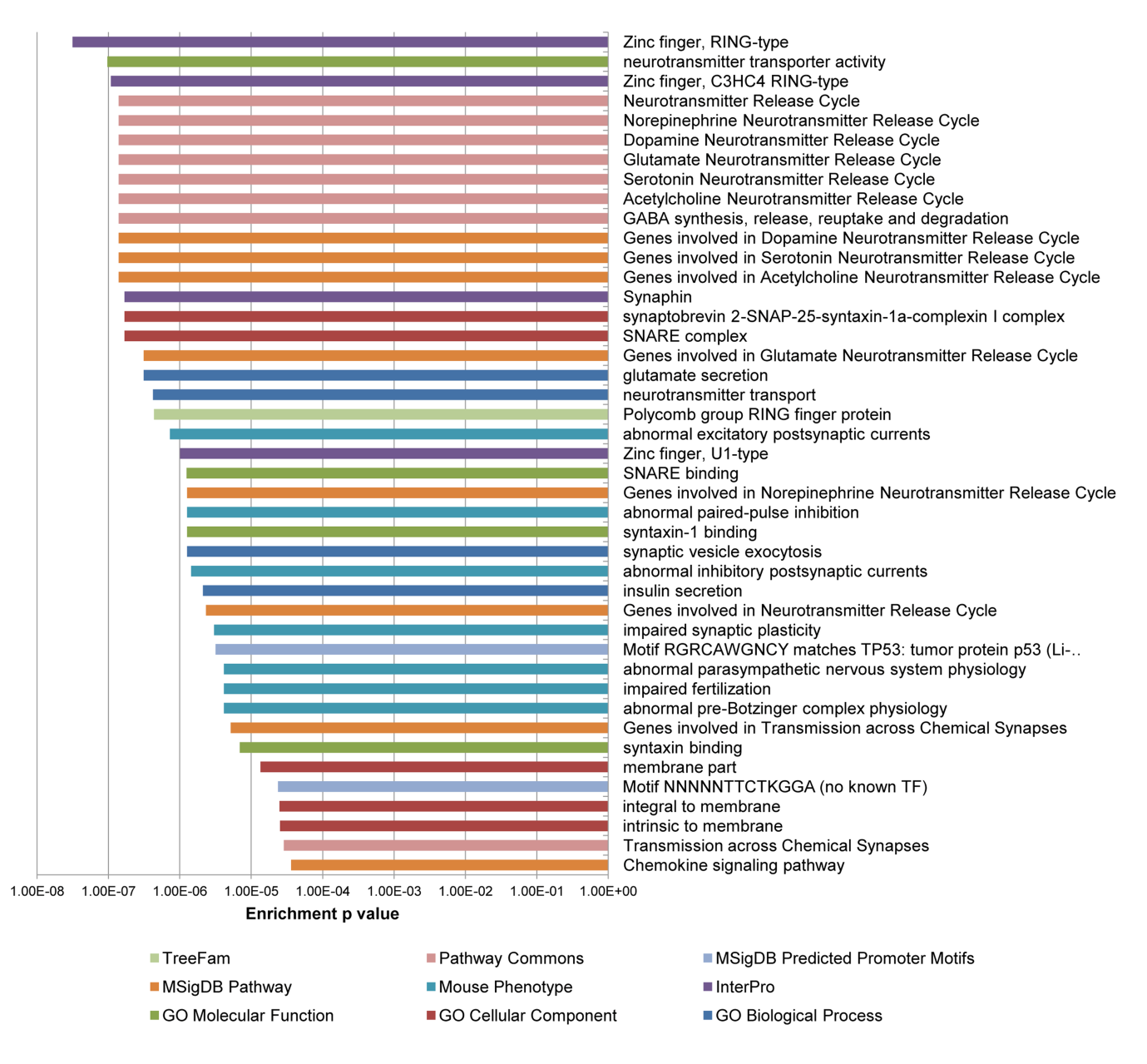


**Figure S9**: Enrichment of key neurological annotation terms at sites of increased methylation following an Alu element insertion within 2 Kb in the human brain. Uncorrected p values are shown with all displayed terms having an FDR q value < 0.01.


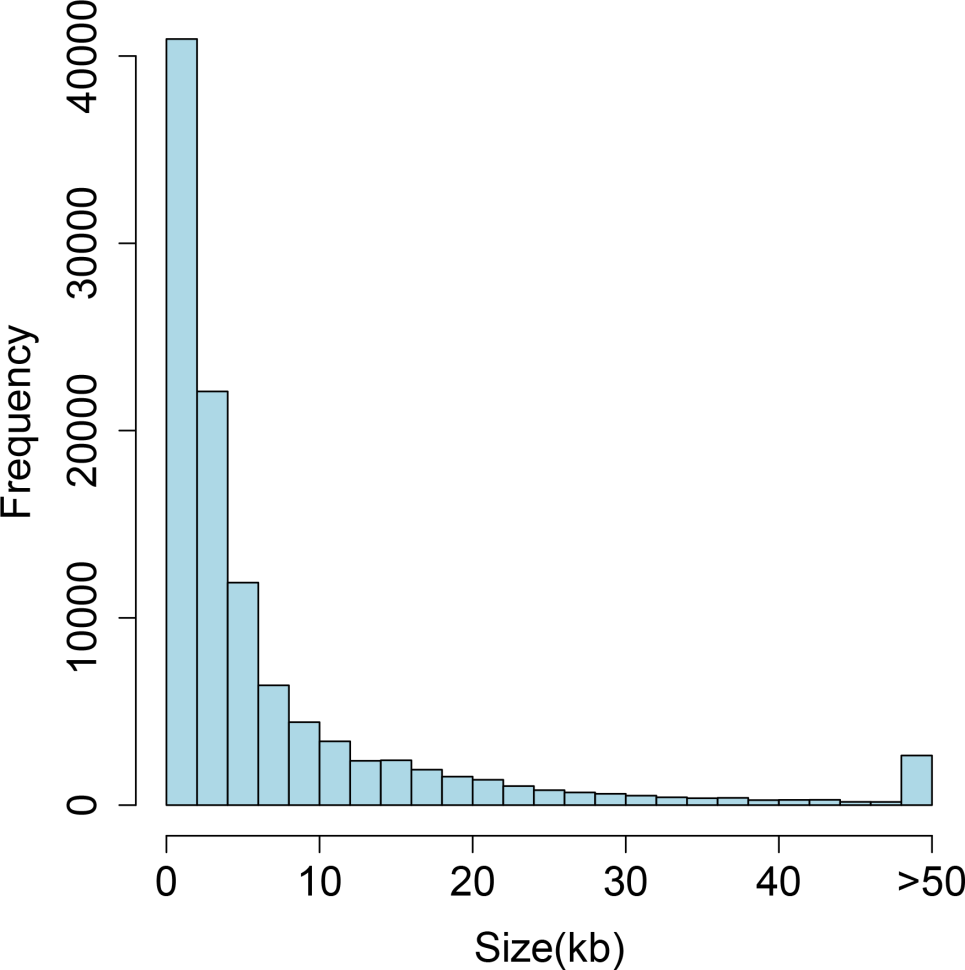


**Figure S10**: Size distribution of human segmentally duplicated regions.


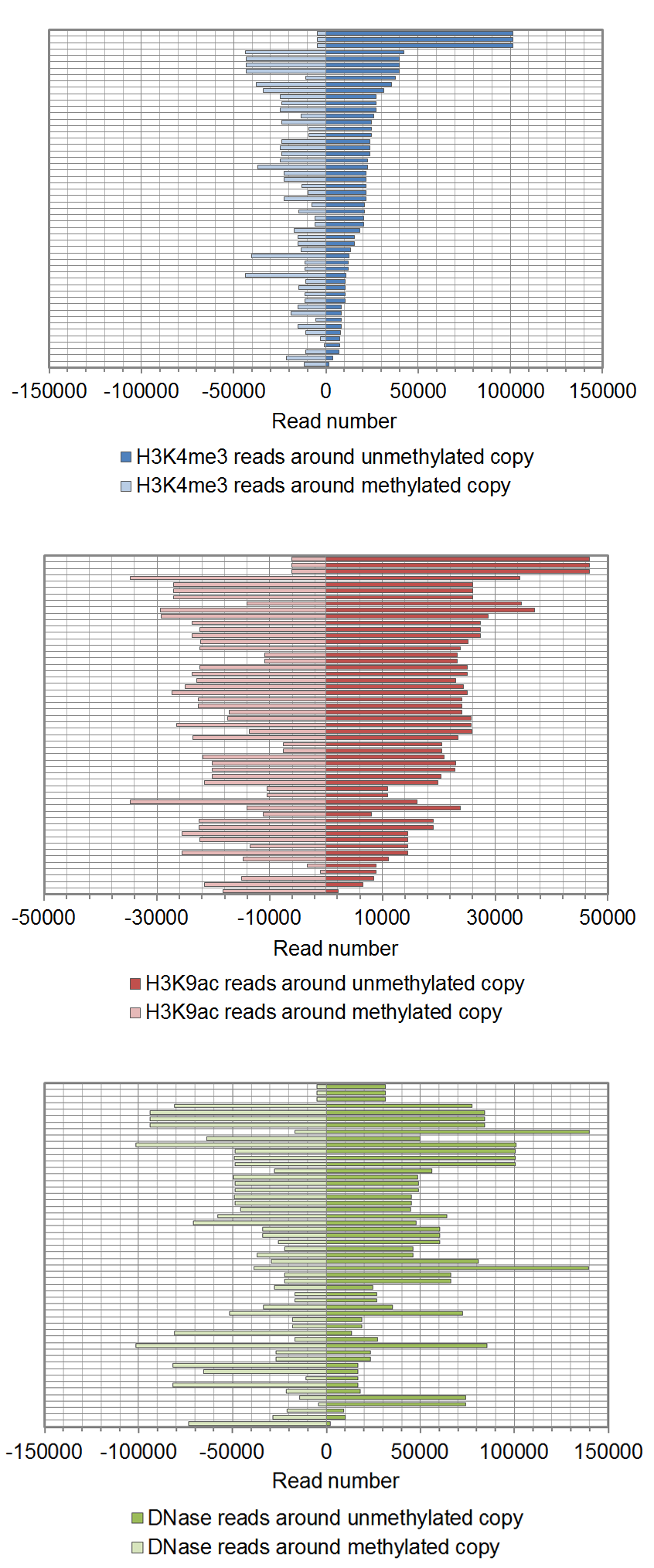


**Figure S11**: The same chromatin marks shown in Figure 5 to be divergent in their levels immediately around discordantly methylated paralogous CpG sites but with the window size increased to 500kb either side of the corresponding sites. None of the 26 chromatin marks examined were consistently enriched around the methylated or unmethylated copies of these sites within this expanded window size (minimum observed Holm adjusted binomial p value = 0.33) suggesting the divergence observed in Figure 5 is not a feature of the larger genomic region.
